# Supplementary material for: Assessment on patient outcomes of primary hip replacement: an interrupted time series analysis from ‘The National Joint Registry of England and Wales’
Source: BMJ Open. 2019 Nov 21;9(11):e031599. doi: 10.1136/bmjopen-2019-031599 (PMC6887059; doi:10.1136/bmjopen-2019-031599)
Supplement: Supplementary data [file bmjopen-2019-031599supp001.pdf]

MANUSCRIPT TITLE:

Impact of a National Enhanced Recovery Programme on Patient Outcomes of Primary Hip Replacement: an Interrupted Time Series Analysis from England.

APPENDIXES

Appendix 1. Cost methods.

Objective

We aimed to estimate the trend in National Health Service (NHS) expenditure over time, reflecting the change in length of stay (LOS) observed.

Grouper and reference cost methods

Using Hospital Episode Statistics (HES) data for the same group of patients as for LOS (i.e. excluding those with length of stay above 15 days), we generated healthcare resource use group (HRG) classifications for the index episode for each patient using the 2015/16 NHS reference costs grouper [1], which were subsequently used to estimate inpatient costs per patient using NHS reference costs from 2015/16 [2].

86% of the index episodes in the data had a LOS below the trim point for the relevant HRG. For example, the most common HRG in the data was hip replacement without complications (HN12F: "Very Major Hip Procedures for Non-Trauma with CC Score 0-1" [2]), constituting 63% of the index episodes in the data. This HRG has a trim point of 6 days, meaning that all episodes with a duration less than 6 days are assigned the same unit cost. A reduction in LOS within the trim point is therefore not reflected in the cost of the episode, despite there being a true reduction in NHS costs. In order to estimate the mean change in NHS expenditure we therefore estimated an adjusted average bed day cost.

Garriga C, *et al. BMJ Open* 2019; 9:e031599. doi: 10.1136/bmjopen-2019-031599

Estimating the adjusted average bed day cost

For each HRG we estimated the average cost per bed day (defined as any part of a day spent in hospital) by dividing the total cost of the index episodes for that HRG by the total number of bed days for that HRG. This generated a single average bed day cost per HRG.

For each patient we estimated the adjusted episode cost by multiplying their length of stay (bed days) by the average bed day cost for the HRG that they had been assigned by the NHS reference costs grouper [1]. Therefore, instead of assigning the same unit cost to all patients with the same HRG who had a LOS below the trim point, the adjusted cost differed according to a patient's LOS, even if that LOS was below the trim point for the HRG. Using this method we were able to estimate the average difference in true NHS expenditure as a result of the reduction in length of stay over time even when the LOS was below the trim point.

The 2015/16 grouper and reference costs [1,2] were used to estimate costs for all patients in all years, as there are differences in the methodologies used for HRG classification in different cost years [3]. This prevents a like-for-like comparison between years if different groupers and/or costs are used.

Costs were estimated for a total of 432,143 patients.

References for Appendix 1

1. HRG4+ 2015/16 Reference Costs Grouper. Copyright © 2015 Health and Social Care Information Centre. Grouper version: RC 15/16. Implementation version: 1516.RC.8
2. Department of Health. NHS reference costs 2015 to 2016. https://www.gov.uk/government/publications/nhs-reference-costs-2015-to-2016
3. Reference Costs 2015-16. Department of Health. https://assets.publishing.service.gov.uk/government/uploads/system/uploads/attachment_data/file/577083/Reference_Costs_2015-16.pdf.

Appendix 2. Operative procedure codes (OPCS 4.8) that we used to identify hip revision in the Hospital Episode Statistics (HES) registry.

Procedure type 1: : W39.4, "Attention to total prosthetic replacement of hip joint (Not Elsewhere Classified, NEC)"; W39.6, "Closed reduction of dislocated total prosthetic replacement of hip joint"; W78.2, "Release of contracture of hip joint"; W95.4, "Attention to hybrid prosthetic replacement of hip joint using cement NEC"; X09.1, "Hindquarter amputation"; X09.2, "Disarticulation of hip"; X09.3, "Amputation of leg above knee".

Procedure type 2: W07.2, "Excision of periarticular ectopic bone"; W18.1, "Fenestration of cortex of bone"; W18.2, "Saucerisation of bone"; W18.3, "Sequestrectomy of bone"; W18.4, "Decompression of fourage of bone"; W18.5, "Insertion of drainage system into bone"; W33.6, "Debridement of bone NEC"; W33.7, "Lavage of bone"; W33.8, "Other specified other open operations on bone"; W33.9, "Unspecified other open operations on bone"; W45.4, "Attention to total prosthetic replacement of joint NEC"; W54.4, "Attention to prosthetic interposition arthroplasty of joint NEC"; W60.3, "Conversion to arthrodesis and extra-articular bone graft NEC"; W60.8, "Other specified fusion of other joint and extra-articular bone graft"; W60.9, "Unspecified fusion of other joint and extra-articular bone graft"; W61.3, "Conversion to arthrodesis and articular bone graft NEC"; W61.8, "Other specified fusion of other joint and other articular bone graft"; W61.9, "Unspecified fusion of other joint and other articular bone graft"; W63.1, "Revision of arthrodesis and internal fixation NEC"; W63.2, "Revision of arthrodesis and external fixation NEC"; W63.8, "Other specified revisional fusion of other joint"; W63.9, "Unspecified revisional fusion of other joint"; W64.0, "Conversion from previous arthrodesis NEC"; W64.1, "Conversion to arthrodesis and internal fixation NEC"; W64.2, "Conversion to arthrodesis and external fixation NEC"; W64.8, "Other specified conversion to fusion of other joint"; W64.9, "Unspecified conversion to fusion of other joint"; W69.1, "Total synovectomy"; W69.2,

Garriga C, *et al. BMJ Open* 2019; 9:e031599. doi: 10.1136/bmjopen-2019-031599

“Subtotal synovectomy”; W69.3, “Partial synovectomy”; W69.4, “Open biopsy of synovial membrane of joint”; W69.5, “Open division of synovial plica”; W69.8, “Other specified open operations on synovial membrane of joint”; W69.9, “Unspecified open operations on synovial membrane of joint”; W71.2, “Open excision of intra-articular osteophyte”; W71.3, “Forage of joint”; W71.8, “Other specified other open operations on intra-articular structure”; W71.9, “Unspecified other open operations on intra-articular structure”; W77.1, “Repair of capsule of joint for stabilisation of joint NEC”; W77.2, “Transposition of muscle for stabilisation of joint”; W77.3, “Blocking operations on joint using prosthesis for stabilisation of joint”; W77.4, “Blocking operations on joint using bone for stabilisation of joint”; W77.5, “Periarticular osteotomy for stabilisation of joint”; W77.8, “Other specified stabilising operations on joint”; W77.9, “Unspecified stabilising operations on joint”; W78.4, “Limited release of contracture of capsule of joint”; W78.8, “Other specified release of contracture of joint”; W78.9, “Unspecified release of contracture of joint”; W80.1, “Open debridement and irrigation of joint”; W80.2, “Open debridement of joint NEC”; W80.3, “Open irrigation of joint NEC”; W80.8, “Other specified debridement and irrigation of joint”; W80.9, “Unspecified debridement and irrigation of joint”; W81.1, “Excision of lesion of joint NEC”; W81.2, “Open removal of loose body from joint”; W81.3, “Drainage of joint”; W81.4, “Incision of joint NEC”; W81.5, “Exploration of joint NEC”; W81.6, “Capsulorrhaphy of joint”; W81.7, “Insertion of therapeutic spacer into joint”; W81.8, “Other specified other open operations on joint”; W81.9, “Unspecified other open operations on joint”; W84.3, “Endoscopic division of synovial plica”; W84.4, “Endoscopic decompression of joint”; W84.6, “Endoscopic excision of synovial plica”; W84.8, “Other specified therapeutic endoscopic operations on other joint structure”; W84.9, “Unspecified therapeutic endoscopic operations on other joint structure”; W86.1, “Endoscopic removal of loose body from joint NEC”; W86.8, “Other specified therapeutic endoscopic operations on cavity of other joint”;

Garriga C, *et al. BMJ Open* 2019; 9:e031599. doi: 10.1136/bmjopen-2019-031599

W86.9, “Unspecified therapeutic endoscopic operations on cavity of other joint”; W88.1, “Diagnostic endoscopic examination of joint and biopsy of lesion of joint NEC”; W88.8, “Other specified diagnostic endoscopic examination of other joint”; W88.9, “Unspecified diagnostic endoscopic examination of other joint”; W90.1, “Aspiration of joint”; W90.2, “Arthrography”; W90.3, “Injection of therapeutic substance into joint”; W90.4, “Injection into joint NEC”; W90.8, “Other specified puncture of joint”; W90.9, “Unspecified puncture of joint”; W91.1, “Manipulation of joint using traction NEC”; W91.3, “Manipulation of prosthetic joint NEC”; W91.8, “Other specified other manipulation of joint”; W91.9, “Unspecified other manipulation of joint”; W92.1, “Biopsy of lesion of joint NEC”; W92.2, “Distension of joint”; W92.3, “Examination of joint under image intensifier”; W92.4, “Examination of joint under anaesthetic”; W92.5, “Examination of joint NEC”; W92.8, “Other specified other operations on joint”; W92.9, “Unspecified other operations on joint”; O19.8, “Other specified other therapeutic endoscopic operations on other joint structure”; O27.1, “Extra-articular ligament reconstruction for stabilisation of joint”; O27.8, “Other specified other stabilising operations on joint”; O27.9, “Unspecified other stabilising operations on joint”; S57.1, “Debridement of skin NEC”; S57.2, “Removal of slough from skin NEC”; S57.3, “Toilet of skin NEC”; S57.4, “Dressing of skin NEC”; S57.5, “Attention to dressing of skin NEC”; S57.6, “Cleansing and sterilisation of skin NEC”; S57.7, “Dressing of skin using vacuum assisted closure device NEC”; S57.8, “Other specified exploration of other skin of other site”; S57.9, “Unspecified exploration of other skin of other site”; S58.2, “Larvae debridement therapy of skin NEC”; X09.8, “Other specified amputation of leg”; X09.9, “Unspecified amputation of leg”.

Site for revision: Z84.3, “Hip joint”; Z76.1, “Head of femur”; Z75.6, “Acetabulum”; Z84.9, “Joint of pelvis or upper leg NEC”.

Garriga C, *et al. BMJ Open* 2019; 9:e031599. doi: 10.1136/bmjopen-2019-031599

Algorithm: One code from procedure type 1 or a combination of one code from procedure type 2 and site for revision were used to identify hip revision.

Appendix 3. Codes defined in the International Statistical Classification of Diseases and Related Health Problems 10th Revision (ICD-10) that we used to identify complications in the Hospital Episode Statistics (HES) registry.

Stroke: I60.X, “Subarachnoid haemorrhage”; I61.0, “Intracerebral haemorrhage in hemisphere, subcortical”; I61.1, “Intracerebral haemorrhage in hemisphere, cortical”; I61.2, “Intracerebral haemorrhage in hemisphere, unspecified”; I61.3, “Intracerebral haemorrhage in brain stem”; I61.4, “Intracerebral haemorrhage in cerebellum”; I61.5, “Intracerebral haemorrhage, intraventricular”; I61.6, “Intracerebral haemorrhage, multiple localized”; I61.8, “Other intracerebral haemorrhage”; I61.9, “Intracerebral haemorrhage, unspecified”; I63.0, “Cerebral infarction due to thrombosis of precerebral arteries”; I63.1, “Cerebral infarction due to embolism of precerebral arteries”; I63.2, “Cerebral infarction due to unspecified occlusion or stenosis of precerebral arteries”; I63.3, “Cerebral infarction due to thrombosis of cerebral arteries”; I63.4, “Cerebral infarction due to embolism of cerebral arteries”; I63.5, “Cerebral infarction due to unspecified occlusion or stenosis of cerebral arteries”; I63.6, “Cerebral infarction due to cerebral venous thrombosis, nonpyogenic”; I63.8, “Other cerebral infarction”; I63.9, “Cerebral infarction, unspecified”; and I64.X, “Stroke, not specified as haemorrhage or infarction”.

Respiratory infection: J12.X, “Viral pneumonia, not elsewhere classified: bronchopneumonia due to viruses other than influenza viruses”; J13, “Pneumonia due to *Streptococcus pneumoniae*”; J14, “Pneumonia due to *Haemophilus influenzae*”; J15.X, “Bacterial pneumonia, not elsewhere classified: bronchopneumonia due to bacteria other than *S. pneumoniae* and *H. influenzae*”; J18.0, “Bronchopneumonia, unspecified. Excluding bronchiolitis”; J18.1, “Lobar pneumonia, unspecified”; J18.2, “Hypostatic pneumonia, unspecified”; J18.8, “Other pneumonia, organism unspecified”; J18.9, “Pneumonia, unspecified”; J22, “Unspecified acute lower respiratory infection”; J44.0, “Chronic

Garriga C*, et al. BMJ Open* 2019; 9:e031599. doi: 10.1136/bmjopen-2019-031599

obstructive pulmonary disease with acute lower respiratory infection. Excluding with influenza"; J44.1, "Chronic obstructive pulmonary disease with acute exacerbation, unspecified"; J69.0, "Pneumonitis due to food and vomit. Excluding Mendelson syndrome"; J69.1, "Pneumonitis due to oils and essences"; J69.8, "Pneumonitis due to other solids and liquids. Pneumonitis due to aspiration of blood"; and J85.1, "Abscess of lung with pneumonia. Excluding with pneumonia due to specified organism".

Acute myocardial infarction: I21.0, "Acute transmural myocardial infarction of anterior wall"; I21.1, "Acute transmural myocardial infarction of inferior wall"; I21.2, "Acute transmural myocardial infarction of other sites"; I21.3, "Acute transmural myocardial infarction of unspecified site"; I21.4, "Acute subendocardial myocardial infarction"; and I21.9, "Acute myocardial infarction, unspecified".

Pulmonary embolism/deep vein thrombosis: I80.1, "Phlebitis and thrombophlebitis of superficial vessels of lower extremities"; I80.1, "Phlebitis and thrombophlebitis of femoral vein"; I80.3, "Phlebitis and thrombophlebitis of other deep vessels of lower extremities"; I26.0, "Pulmonary embolism with mention of acute cor pulmonale"; and I26.9, "Pulmonary embolism without mention of acute cor pulmonale".

Urinary tract infection: N30.0, "Acute cystitis. Excluding irradiation cystitis and trigonitis"; and N39.0, "Urinary tract infection, site not specified".

Wound disruption: T81.3, "Disruption of operation wound, not elsewhere classified".

Surgical site infection: T81.4, "Infection following a procedure, not elsewhere classified".

Fracture after implant: M96.6, "Fracture of bone following insertion of orthopaedic implant, joint prosthesis, or bone plate. Excluding complication of internal orthopaedic devices, implants or grafts".

Garriga C, *et al. BMJ Open* 2019; 9:e031599. doi: 10.1136/bmjopen-2019-031599

Complication of prosthesis: T84.0, “Mechanical complication of internal joint prosthesis”.

Neurovascular injury: T81.2, “Accidental puncture and laceration during a procedure, not elsewhere classified. Accidental perforation of: blood vessel, nerve or organ by: catheter, endoscope, instrument or probe during a procedure”.

Acute renal failure: N17.0, “Acute renal failure with tubular necrosis”; N17.1, “Acute renal failure with acute cortical necrosis”; N17.2, “Acute renal failure with medullary necrosis”; N17.8, “Other acute renal failure”; and N17.9, “Acute renal failure, unspecified”.

Garriga C, *et al. BMJ Open* 2019; 9:e031599. doi: 10.1136/bmjopen-2019-031599

Appendix 4. Operative procedure codes (OPCS 4.8) that we used to identify blood-transfusion complication in the Hospital Episode Statistics (HES) registry.

X33.2, “Intravenous blood transfusion of packed cells”; X33.3, “Intravenous blood transfusion of platelets”; X33.8, “Other specified other blood transfusion”; X33.9, “Unspecified other blood transfusion”; X33.1, “Intra-arterial blood transfusion”; X33.7, “Autologous transfusion of red blood cells”; X34.1, “Transfusion of coagulation factor”; X34.2, “Transfusion of plasma not elsewhere classified”; X34.3, “Transfusion of serum not elsewhere classified”; and X34.4, “Transfusion of blood expander”.

Garriga C, *et al. BMJ Open* 2019; 9:e031599. doi: 10.1136/bmjopen-2019-031599

Appendix 5. Codes defined in the International Statistical Classification of Diseases and Related Health Problems 10th Revision (ICD-10) that we used to identify comorbidities in the Hospital Episode Statistics (HES) registry.

Myocardial infarction: I21, “Acute myocardial infarction”; and I22, “Subsequent myocardial infarction”.

Congestive heart failure: I50.0, “Congestive heart failure”.

Peripheral vascular disease: I70, “Atherosclerosis”; I71, “Aortic aneurysm and dissection”; I72, “Other aneurysm and dissection”; and I73, “Other peripheral vascular diseases”.

Cerebrovascular disease: I60, “Subarachnoid haemorrhage”; I61, “Intracerebral haemorrhage”; I62, “Other nontraumatic intracranial haemorrhage”; I63, “Cerebral infarction”; I64, “Stroke, not specified as haemorrhage or infarction”; I65, “Occlusion and stenosis of precerebral arteries, not resulting in cerebral infarction”; I66, “Occlusion and stenosis of cerebral arteries, not resulting in cerebral infarction”; and I67, “Other cerebrovascular diseases”.

Dementia: F00, “Dementia in Alzheimer disease”; F01, “Vascular dementia”; F02, “Dementia in other diseases classified elsewhere”; and F03, “Unspecified dementia”.

Chronic obstructive pulmonary disease: J41, “Simple and mucopurulent chronic bronchitis”; J42, “Unspecified chronic bronchitis”; J43, “Emphysema”; J44, “Other chronic obstructive pulmonary disease”; J45, “Asthma”; J46, “Status asthmaticus”; and J47, “Bronchiectasis”.

Connective tissue disease: M05, “Seropositive rheumatoid arthritis”; M06, “Other rheumatoid arthritis”; M08, “Juvenile arthritis”; M15, “Polyarthrosis”; M16, “Coxarthrosis [arthrosis of hip]”; M17, “Gonarthrosis [arthrosis of knee]”; M18, “Arthrosis of first carpometacarpal joint”; M19, “Other arthrosis” ; M35, “Other systemic involvement of

Garriga C, *et al. BMJ Open* 2019; 9:e031599. doi: 10.1136/bmjopen-2019-031599

connective tissue" ; and M36, "Systemic disorders of connective tissue in diseases classified elsewhere".

<u>Peptic ulcer disease</u>: K25, "Gastric ulcer"; K26, "Duodenal ulcer"; K27, "Peptic ulcer, site unspecified"; and K28, "Gastrojejunal ulcer".

<u>Mild liver disease</u>: K70.0, "Alcoholic fatty liver"; K76.0, "Fatty (change of) liver, not elsewhere classified"; and K76.1 "Chronic passive congestion of liver".

<u>Mild diabetes (without end organ damage - include ketoacidosis and coma)</u>: E10.X, "Insulin-dependent diabetes mellitus"; E10.0, "Insulin-dependent diabetes mellitus with coma"; E10.1, "Insulin-dependent diabetes mellitus with ketoacidosis" ; E10.9, "Insulin-dependent diabetes mellitus without complications"; E11.X, "Non-insulin-dependent diabetes mellitus"; E11.0, "Non-insulin-dependent diabetes mellitus with coma"; E11.1, "Non-insulin-dependent diabetes mellitus with ketoacidosis" ; E11.9, "Non-insulin-dependent diabetes mellitus without complications"; E12.X, "Malnutrition-related diabetes mellitus"; E12.0, "Malnutrition-related diabetes mellitus with coma"; E12.1, "Malnutrition-related diabetes mellitus with ketoacidosis"; E12.9, "Malnutrition-related diabetes mellitus without complications"; E13.X, "Other specified diabetes mellitus"; E13.0, "Other specified diabetes mellitus with coma"; E13.1, "Other specified diabetes mellitus with ketoacidosis"; E13.9, "Other specified diabetes mellitus without complications"; E14.X, "Unspecified diabetes mellitus"; E14.0, "Unspecified diabetes mellitus with coma"; E14.1, "Unspecified diabetes mellitus with ketoacidosis"; and E14.9, "Unspecified diabetes mellitus without complications" .

<u>Hemiplegia</u>: G81, "Hemiplegia. Excluding congenital cerebral palsy"

Garriga C, *et al. BMJ Open* 2019; 9:e031599. doi: 10.1136/bmjopen-2019-031599

Moderate/severe renal disease: N17, “Acute renal failure”; N18, “Chronic kidney disease”; and N19, “Unspecified kidney failure”.

Severe diabetes (i.e. with organ damage): E10, “Insulin-dependent diabetes mellitus”; E11, “Non-insulin-dependent diabetes mellitus”; E12, “Malnutrition-related diabetes mellitus”; E13, “Other specified diabetes mellitus”; or E14, “Unspecified diabetes mellitus”. Complicated with: .2, “With renal complications”; .3 “With ophthalmic complications”; .4, “With neurological complications”; .5, “With peripheral circulatory complications”; .6, “With other specified complications”; .7, “With multiple complications”; or .8, “With unspecified complications”. N083, “Glomerular disorders in diabetes mellitus”.

Tumour: C00-C75, “Malignant neoplasms, stated or presumed to be primary, of specified sites, except of lymphoid, haematopoietic and related tissue”; C76, “Malignant neoplasm of other and ill-defined sites”; C80, “Malignant neoplasm, without specification of site”; C88, “Malignant immunoproliferative diseases”; C90.0, “Multiple myeloma”; C90.2, “Extramedullary plasmacytoma”; C96, “Other and unspecified malignant neoplasms of lymphoid, haematopoietic and related tissue”; C97, “Malignant neoplasms of independent (primary) multiple sites”; D00-D09, “In situ neoplasms”; D10-D36, “Benign neoplasms”; and D37-D48, “Neoplasms of uncertain or unknown behaviour”.

Leukaemia: C90.1, “Plasma cell leukaemia”; C91, “Lymphoid leukaemia”; C92, “Myeloid leukaemia”; C93, “Monocytic leukaemia”; C94, “Other leukaemias of specified cell type”; and C95 “Leukaemia of unspecified cell type”.

Lymphoma: C81, “Hodgkin lymphoma”; C82, “Follicular lymphoma”; C83, “Non-follicular lymphoma”; C84, “Mature T/NK-cell lymphomas”; and C85, "Other and unspecified types of non-Hodgkin lymphoma”.

Garriga C, *et al. BMJ Open* 2019; 9:e031599. doi: 10.1136/bmjopen-2019-031599

Moderate/severe liver disease: K70, “Alcoholic liver disease except”; K71, “Toxic liver disease”; K72, “Hepatic failure, not elsewhere classified”; K73, “Chronic hepatitis, not elsewhere classified”; K74, “Fibrosis and cirrhosis of liver”; K75, “Other inflammatory liver diseases”; and K76, “Other diseases of liver”. Excluding codes for mild liver disease: K70.0, K76.0 and K76.1.

AIDS: B20, “HIV disease resulting in infectious and parasitic diseases”; B21, “HIV disease resulting in malignant neoplasms”; B22, “HIV disease resulting in other specified diseases”; and B23 “HIV disease resulting in other conditions”.

Metastatic solid tumour: C77, “Secondary and unspecified malignant neoplasm of lymph nodes”; C78, “Secondary malignant neoplasm of respiratory and digestive organs”; and C79, “Secondary malignant neoplasm of other and unspecified sites”.

Garriga C, *et al. BMJ Open* 2019; 9:e031599. doi: 10.1136/bmjopen-2019-031599
