# Supplementary material for: Assessment on patient outcomes of primary hip replacement: an interrupted time series analysis from ‘The National Joint Registry of England and Wales’
Source: BMJ Open. 2019 Nov 21;9(11):e031599. doi: 10.1136/bmjopen-2019-031599 (PMC6887059; doi:10.1136/bmjopen-2019-031599)
Supplement: Supplementary data [file bmjopen-2019-031599supp002.pdf]

MANUSCRIPT TITLE: Assessment of a National Enhanced Recovery Programme on Patient Outcomes of Primary Hip Replacement: an Interrupted Time Series Analysis from "The National Joint Registry of England and Wales".

SUPPLEMENTARY MATERIAL

Supplementary Text S1. Patient and public involvement

During study development, we identified a person who cares for a wife with extensive arthritis and has himself recently undergone hip replacement. He considered this a worthwhile and important project, but cautioned that enhanced recovery could be perceived as a way for hospitals to discharge patients early to save money.

The study was developed in collaboration with the University of Bristol Musculoskeletal Research Unit's Patient Experience Partnership in Research (PEP-R) group, comprising patient representatives with experience of both chronic musculoskeletal conditions and joint replacement. They felt that discharge within four days should not become an NHS target, but that discharge time should instead be tailored to individual needs. They identified specific elements of enhanced recovery that they felt could have had a big impact on their recovery.

The James Lind Alliance has identified the need for involving patients in identifying outcomes that matter to them (patient-identified outcomes). We conducted a patient forum with the PEP-R group to identify outcomes from those available in the routine datasets available for this study. The top outcomes were: 1) pain and function, 2) complications (particularly hospital-acquired infection), 3) length of stay (dependent on the level of support at home), 4) revision surgery, and 5) mortality (rated low importance).

We met with the PEP-R group to report our findings of the effect of the ERAS programme in primary hip replacement on patient outcomes. The group wanted to see graphs of trends in pain, function, and other outcomes, stratified by age group and co-morbidity. They wanted the graphs to be clear, particularly the scale used when outcomes were rare as a restricted scale could be visually misleading. They also wanted the graphs to show the overall trend rather than trend before, during, and after the implementation of ERAS. They found the study finding that reduced length of stay did not adversely affect patient outcomes to be positive.

Supplementary Figure S1. Trends in cost per bed day following primary hip replacement in England, UK, 2008 – 2016, by month.

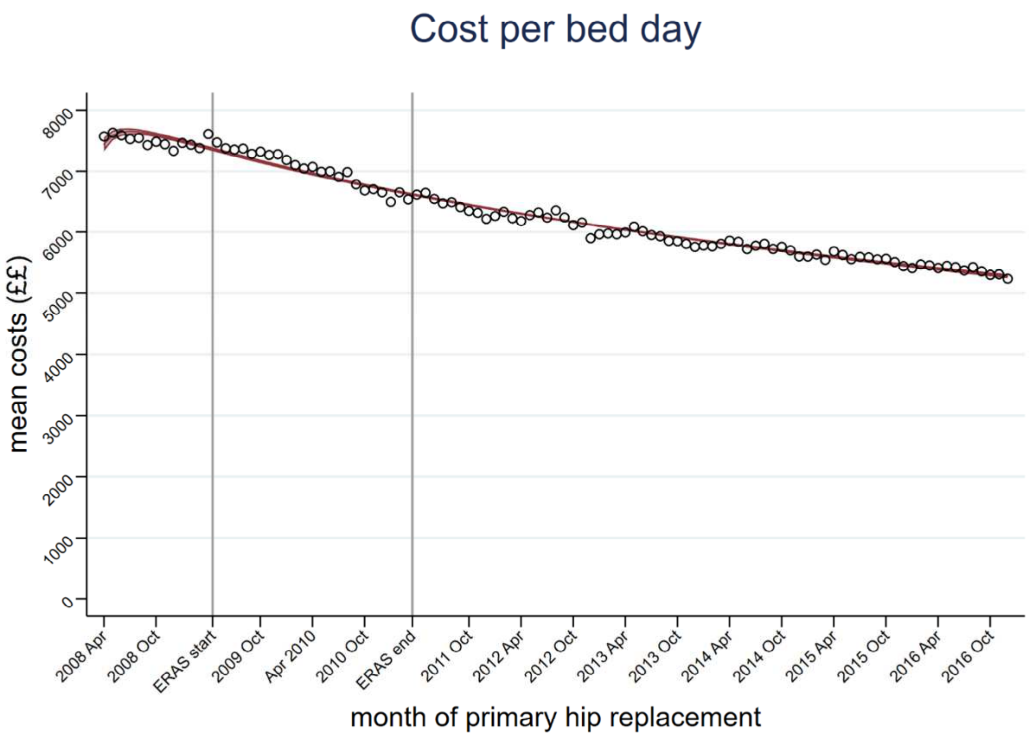

Enhanced recovery after surgery programme implemented in England from April 2009 to March 2011, ERAS.

Garriga C, *et al. BMJ Open* 2019; 9:e031599. doi: 10.1136/bmjopen-2019-031599

Supplementary Figure S2. Trends in change in Oxford hip score following primary hip replacement according to patient age categories in England, UK, 2008 – 2016, by month.

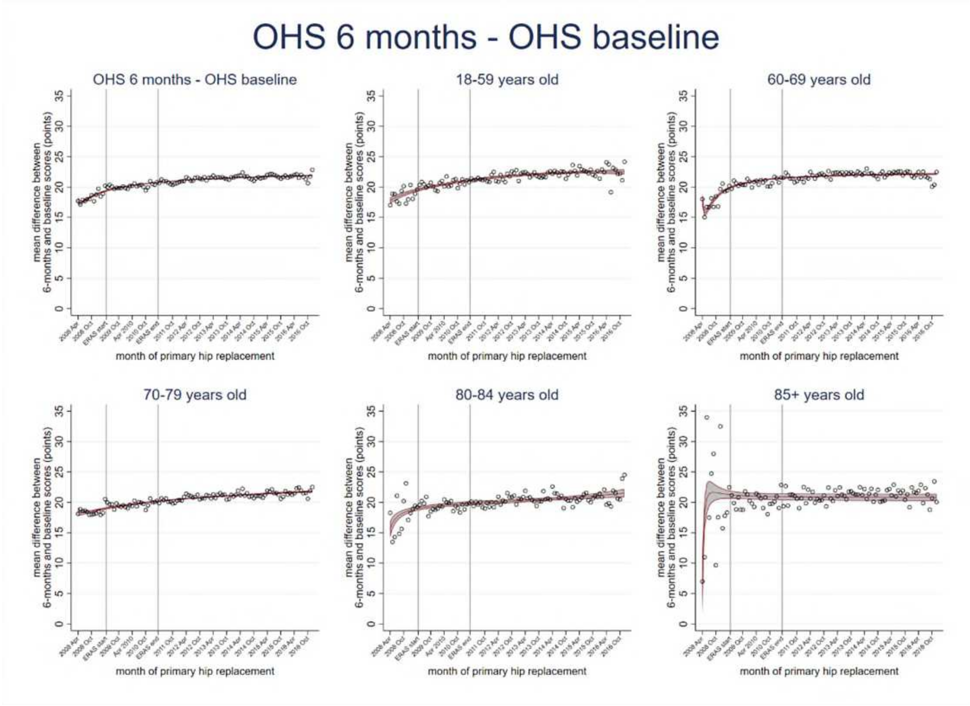

Oxford hip score, OHS; enhanced recovery after surgery programme implemented in England from April 2009 to March 2011, ERAS.

Garriga C, *et al. BMJ Open* 2019; 9:e031599. doi: 10.1136/bmjopen-2019-031599

Supplementary Figure S3. Trends in change in Oxford hip score following primary hip replacement according to whether patients do or do not present with comorbidities, in England, UK, 2008 – 2016, by month.

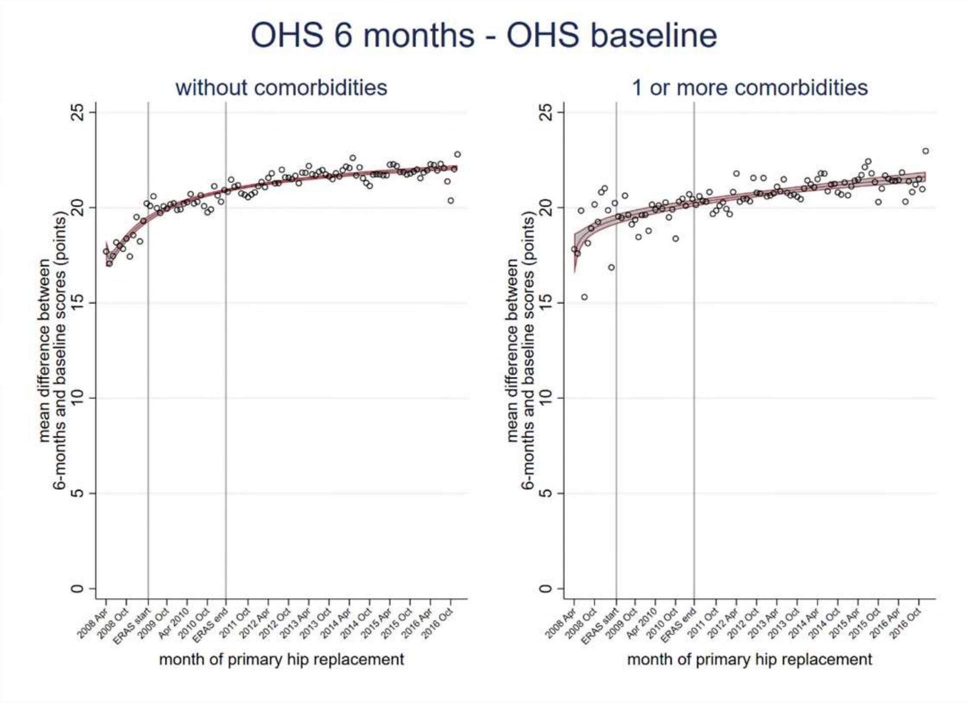

Oxford hip score, OHS; enhanced recovery after surgery programme implemented in England from April 2009 to March 2011, ERAS.

Supplementary Table S1. Temporal trends in patients underwent planned primary hip replacement from April 2008 to December 2016. Full models with Newey-West standard errors.

Parameter	Coefficient	Lower 95% CI	Upper 95% CI	*P*-value
Length of stay in hospital				
Intercept	5.674	5.655	5.693	<0.001
Monthly trend	-0.020	-0.023	-0.017	<0.001
Level change $ERAS_0$	0.176	0.120	0.232	<0.001
Trend change after $ERAS_0$	-0.013	-0.017	-0.009	<0.001
Level change $ERAS_{end}$	-0.102	-0.203	-0.001	0.049
Trend change after $ERAS_{end}$	0.019	0.015	0.022	<0.001
Change in Oxford hip score (score at 6 months – score at baseline)				
Intercept	17.063	16.896	17.230	<0.001
Monthly trend	0.158	0.130	0.186	<0.001
Level change $ERAS_0$	0.772	0.538	1.006	<0.001
Trend change after $ERAS_0$	-0.131	-0.161	-0.101	<0.001
Level change $ERAS_{end}$	0.564	0.208	0.920	0.002
Trend change after $ERAS_{end}$	-0.013	-0.025	-0.001	0.039
Complication rates by 6 months after surgery				
Intercept	4.254	4.161	4.347	<0.001
Monthly trend	-0.072	-0.088	-0.056	<0.001
Level change $ERAS_0$	-0.729	-1.531	0.072	0.074
Trend change after $ERAS_0$	0.011	-0.037	0.060	0.648

Garriga C, *et al. BMJ Open* 2019; 9:e031599. doi: 10.1136/bmjopen-2019-031599

Level change $ERAS_{end}$	0.179	-0.372	0.730	0.521
Trend change after $ERAS_{end}$	0.057	0.006	0.107	0.029
Revision rates by 5 years after surgery				
Intercept	7.755	7.385	8.124	<0.001
Monthly trend	-0.091	-0.138	-0.044	<0.001
Level change $ERAS_0$	0.322	-0.137	0.780	0.164
Trend change after $ERAS_0$	-0.023	-0.083	0.036	0.432
Level change $ERAS_{end}$	0.111	-0.233	0.455	0.518
Trend change after $ERAS_{end}$	0.112	0.066	0.157	<0.001

Confidence intervals, CI; Oxford hip score, OHS; Enhanced Recovery After Surgery, ERAS ; start point of ERAS intervention in April 2009, $ERAS_0$; end point of ERAS intervention in March 2011, $ERAS_{end}$.

Garriga C, *et al. BMJ Open* 2019; 9:e031599. doi: 10.1136/bmjopen-2019-031599

Supplementary Table S2. Temporal trends in patients underwent planned primary hip replacement according to length of stay by age group and comorbidities. April 2008 to December 2016. Parsimonious models with Newey-West standard errors.

**Parameter**	**Coefficient**	**Lower 95% CI**	**Upper 95% CI**	***P*-value**
Length of stay in hospital				
18-59 years old				
Intercept	4.789	4.747	4.831	<0.001
Monthly trend	-0.021	-0.027	-0.016	<0.001
Level change $ERAS_0$	0.189	0.067	0.310	0.003
Trend change after $ERAS_0$	-0.012	-0.019	-0.006	<0.001
Level change $ERAS_{end}$	—	—	—	—
Trend change after $ERAS_{end}$	0.021	0.014	0.027	<0.001
60-69 years old				
Intercept	5.126	5.063	5.188	<0.001
Monthly trend	-0.015	-0.024	-0.006	0.001
Level change $ERAS_0$	0.129	0.047	0.211	0.002
Trend change after $ERAS_0$	-0.017	-0.025	-0.009	<0.001
Level change $ERAS_{end}$	-0.142	-0.238	-0.046	0.004
Trend change after $ERAS_{end}$	0.018	0.015	0.021	<0.001
70-79 years old				
Intercept	5.875	5.858	5.892	<0.001
Monthly trend	-0.015	-0.017	-0.012	<0.001

Garriga C, *et al. BMJ Open* 2019; 9:e031599. doi: 10.1136/bmjopen-2019-031599

Level change $ERAS_0$	0.189	0.133	0.244	<0.001
Trend change after $ERAS_0$	-0.026	-0.030	-0.022	<0.001
Level change $ERAS_{end}$	—	—	—	—
Trend change after $ERAS_{end}$	0.023	0.019	0.028	<0.001
80-84 years old				
Intercept	6.896	6.848	6.943	<0.001
Monthly trend	—	—	—	—
Level change $ERAS_0$	—	—	—	—
Trend change after $ERAS_0$	-0.033	-0.036	-0.030	<0.001
Level change $ERAS_{end}$	-0.179	-0.246	-0.112	<0.001
Trend change after $ERAS_{end}$	0.016	0.012	0.019	<0.001
≥85 years old				
Intercept	7.855	7.801	7.908	<0.001
Monthly trend	—	—	—	—
Level change $ERAS_0$	-0.126	-0.201	-0.051	0.001
Trend change after $ERAS_0$	-0.032	-0.036	-0.027	<0.001
Level change $ERAS_{end}$	—	—	—	—
Trend change after $ERAS_{end}$	0.014	0.008	0.020	<0.001
Without comorbidities				
Intercept	5.554	5.532	5.576	<0.001
Monthly trend	-0.024	-0.027	-0.021	<0.001
Level change $ERAS_0$	0.239	0.168	0.309	<0.001
Trend change after $ERAS_0$	-0.016	-0.022	-0.010	<0.001
Level change $ERAS_{end}$	—	—	—	—

Trend change after $ERAS_{end}$	0.023	0.018	0.029	<0.001
1+ comorbidities				
Intercept	6.218	6.186	6.250	<0.001
Monthly trend	─	─	─	─
Level change $ERAS_0$	─	─	─	─
Trend change after $ERAS_0$	-0.032	-0.036	-0.029	<0.001
Level change $ERAS_{end}$	-0.129	-0.240	-0.018	0.024
Trend change after $ERAS_{end}$	0.018	0.014	0.021	<0.001

Confidence intervals, CI; Enhanced Recovery After Surgery, ERAS ; start point of the ERAS intervention in April 2009, $ERAS_0$; end point of the ERAS intervention in March 2011, $ERAS_{end}$; ─, $P \geq 0.05$.

Garriga C, *et al. BMJ Open* 2019; 9:e031599. doi: 10.1136/bmjopen-2019-031599

Supplementary Table S3. Temporal trends in patients underwent planned primary hip replacement according to length of stay by age group and comorbidities. April 2008 to December 2016. Full models with Newey-West standard errors.

**Parameter**	**Coefficient**	**Lower 95% CI**	**Upper 95% CI**	***P*-value**
Length of stay in hospital				
18-59 years old				
Intercept	4.789	4.747	4.831	<0.001
Monthly trend	-0.021	-0.027	-0.016	<0.001
Level change $ERAS_0$	0.152	0.049	0.255	0.004
Trend change after $ERAS_0$	-0.008	-0.015	-0.001	0.028
Level change $ERAS_{end}$	-0.095	-0.225	0.035	0.149
Trend change after $ERAS_{end}$	0.017	0.011	0.022	<0.001
60-69 years old				
Intercept	5.126	5.063	5.188	<0.001
Monthly trend	-0.015	-0.024	-0.006	0.001
Level change $ERAS_0$	0.129	0.047	0.211	0.002
Trend change after $ERAS_0$	-0.017	-0.025	-0.009	<0.001
Level change $ERAS_{end}$	-0.142	-0.238	-0.046	0.004
Trend change after $ERAS_{end}$	0.018	0.015	0.021	<0.001
70-79 years old				
Intercept	5.875	5.858	5.892	<0.001
Monthly trend	-0.015	-0.017	-0.012	<0.001
Level change $ERAS_0$	0.159	0.125	0.193	<0.001

Garriga C, *et al. BMJ Open* 2019; 9:e031599. doi: 10.1136/bmjopen-2019-031599

Trend change after $ERAS_0$	-0.022	-0.026	-0.019	<0.001
Level change $ERAS_{end}$	-0.076	-0.161	0.008	0.077
Trend change after $ERAS_{end}$	0.020	0.018	0.023	<0.001
80-84 years old				
Intercept	7.016	6.931	7.102	<0.001
Monthly trend	-0.017	-0.029	-0.005	0.005
Level change $ERAS_0$	0.079	-0.019	0.177	0.113
Trend change after $ERAS_0$	-0.016	-0.028	-0.003	0.015
Level change $ERAS_{end}$	-0.181	-0.250	-0.112	<0.001
Trend change after $ERAS_{end}$	0.015	0.011	0.020	<0.001
≥85 years old				
Intercept	7.902	7.743	8.062	<0.001
Monthly trend	-0.007	-0.030	0.016	0.562
Level change $ERAS_0$	-0.070	-0.240	0.100	0.415
Trend change after $ERAS_0$	-0.027	-0.050	-0.004	0.020
Level change $ERAS_{end}$	0.056	-0.083	0.195	0.425
Trend change after $ERAS_{end}$	0.016	0.010	0.022	<0.001
Without comorbidities				
Intercept	5.554	5.532	5.576	<0.001
Monthly trend	-0.024	-0.027	-0.021	<0.001
Level change $ERAS_0$	0.201	0.155	0.247	<0.001
Trend change after $ERAS_0$	-0.012	-0.017	-0.007	<0.001
Level change $ERAS_{end}$	-0.096	-0.202	0.009	0.074
Trend change after $ERAS_{end}$	0.020	0.017	0.022	<0.001

1+ comorbidities

Intercept	6.220	6.134	6.305	<0.001
Monthly trend	-0.001	-0.011	0.010	0.902
Level change $ERAS_0$	0.012	-0.088	0.112	0.813
Trend change after $ERAS_0$	-0.032	-0.042	-0.022	<0.001
Level change $ERAS_{end}$	-0.126	-0.245	-0.007	0.038
Trend change after $ERAS_{end}$	0.018	0.013	0.023	<0.001

Confidence intervals, CI; Enhanced Recovery After Surgery, ERAS ; start point of ERAS intervention in April 2009, $ERAS_0$; end point of ERAS intervention in March 2011, $ERAS_{end}$.

Supplementary Table S4. Distribution of primary hip replacements and percentage of missing values per potential modifier of OHS. England (April 2008 to December 2016)

	**n**	**%**	**% missing values**		
			**OHS at baseline**	**OHS at 6 months**	**OHS change**
Total	438,921	100	38.6	47.9	48.3
Period			*P*<0.001	*P*<0.001	*P*<0.001
Before ERAS (April 2008 - March 2009)	38,362	8.7	87.4	89.6	89.7
During ERAS (April 2009 - March 2011)	86,416	19.7	33.2	41.6	41.9
After ERAS (April 2011 - December 2016)	314,143	71.6	34.1	44.6	45.0
Age at surgery (years)			*P*<0.001	*P*<0.001	*P*<0.001
18-59	78,383	17.9	36.9	52.7	52.9
60-69	13 1,441	30.0	36.1	44.4	44.7
70-79	157.241	35.8	39.0	46.2	46.7
80-84	47,919	10.9	42.5	50.8	51.3
85 or more	23,937	5.5	47.3	56.9	57.5
Comorbidities			*P*<0.001	*P*<0.001	*P*<0.001
None	325,750	74.2	38.3	47.1	47.5
1 or more	113,171	25.8	39.5	50.3	50.7

Oxford hip score, OHS; Enhanced Recovery After Surgery, ERAS . *P* values are calculated from 2-sided Pearson's statistic. OHS change is the difference between OHS at 6 months and OHS at baseline.

Garriga C, *et al. BMJ Open* 2019; 9:e031599. doi: 10.1136/bmjopen-2019-031599

Supplementary Table S5. Distribution of potential modifiers for primary hip replacements with complete and incomplete data for change in OHS (6 month - baseline scores). England (April 2008 to December 2016)

	**Incomplete cases**		**Complete cases**		***P* value**
	**n**	**%**	**n**	**%**	
Period					*P*<0.001
Before ERAS (April 2008 - March 2009)	34,405	16.2	3,957	1.7	
During ERAS (April 2009 - March 2011)	36,242	17.1	50,174	22.1	
After ERAS (April 2011 - December 2016)	141,478	66.7	172,665	76.1	
Age at surgery (years)					*P*<0.001
18-59	41,497	19.6	36,886	16.3	
60-69	58,812	27.7	72,629	32.0	
70-79	73,480	34.6	83,761	36.9	
80-84	24,584	11.6	23,335	10.3	
85 or more	13,752	6.5	10,185	4.5	
Comorbidities					*P*<0.001
None	154,718	72.9	171032.0	75.4	
1 or more	57,407	27.1	55764.0	24.6	

Oxford hip score, OHS; Enhanced Recovery After Surgery, ERAS . *P* values are calculated from 2-sided Pearson's statistic. Incomplete cases defined by a missing value in OHS at baseline or OHS at 6 months.

Supplementary Table S6. Temporal trends in patients underwent planned primary hip replacement according to OHS change by age group and comorbidities. April 2008 to December 2016. Parsimonious models with Newey-West standard errors.

**Parameter**	**Coefficient**	**Lower 95% CI**	**Upper 95% CI**	***P*-value**
Change in Oxford hip score (score at 6 months – score at baseline)				
18-59 years old				
Intercept	18.096	17.853	18.340	<0.001
Monthly trend	0.052	0.034	0.070	<0.001
Level change $ERAS_0$	1.039	0.617	1.461	<0.001
Trend change after $ERAS_0$	—	—	—	—
Level change $ERAS_{end}$	0.334	0.014	0.655	0.041
Trend change after $ERAS_{end}$	-0.031	-0.052	-0.011	0.003
60-69 years old				
Intercept	15.185	14.473	15.897	<0.001
Monthly trend	0.395	0.320	0.471	<0.001
Level change $ERAS_0$				
Trend change after $ERAS_0$	-0.344	-0.433	-0.254	<0.001
Level change $ERAS_{end}$	0.639	0.076	1.203	0.027
Trend change after $ERAS_{end}$	-0.048	-0.074	-0.021	0.001
70-79 years old				
Intercept	18.645	18.474	18.816	<0.001
Monthly trend	-0.047	-0.068	-0.026	<0.001

Garriga C, *et al. BMJ Open* 2019; 9:e031599. doi: 10.1136/bmjopen-2019-031599

Level change $ERAS_0$	1.269	1.109	1.429	<0.001
Trend change after $ERAS_0$	0.068	0.046	0.089	<0.001
Level change $ERAS_{end}$	0.494	0.158	0.829	0.004
Trend change after $ERAS_{end}$	—	—	—	—
80-84 years old				
Intercept	15.235	14.237	16.232	<0.001
Monthly trend	0.358	0.262	0.454	<0.001
Level change $ERAS_0$	—	—	—	—
Trend change after $ERAS_0$	-0.370	-0.480	-0.260	<0.001
Level change $ERAS_{end}$	0.548	0.028	1.068	0.039
Trend change after $ERAS_{end}$	0.034	0.007	0.061	0.016
≥85 years old				
Intercept	19.946	19.233	20.658	<0.001
Monthly trend	—	—	—	—
Level change $ERAS_0$	—	—	—	—
Trend change after $ERAS_0$	—	—	—	—
Level change $ERAS_{end}$	1.249	0.504	1.993	0.001
Trend change after $ERAS_{end}$	—	—	—	—
Without comorbidities				
Intercept	17.106	16.939	17.273	<0.001
Monthly trend	0.133	0.105	0.160	<0.001
Level change $ERAS_0$	1.306	1.088	1.525	<0.001
Trend change after $ERAS_0$	-0.118	-0.147	-0.090	<0.001
Level change $ERAS_{end}$	0.795	0.352	1.237	0.001

Trend change after $ERAS_{end}$	—	—	—	—
1+ comorbidities				
Intercept	16.642	16.003	17.282	<0.001
Monthly trend	0.334	0.260	0.408	<0.001
Level change $ERAS_0$	-1.677	-2.132	-1.223	<0.001
Trend change after $ERAS_0$	-0.279	-0.357	-0.202	<0.001
Level change $ERAS_{end}$	—	—	—	—
Trend change after $ERAS_{end}$	-0.034	-0.056	-0.013	0.002

Oxford hip score, OHS; confidence intervals, CI; Enhanced Recovery After Surgery, ERAS ; start point of ERAS intervention in April 2009, $ERAS_0$; end point of ERAS intervention in March 2011, $ERAS_{end}$; —, $P \geq 0.05$.

Garriga C, *et al. BMJ Open* 2019; 9:e031599. doi: 10.1136/bmjopen-2019-031599

Supplementary Table S7. Temporal trends in patients underwent planned primary hip replacement according to OHS change by age group and comorbidities. April 2008 to December 2016. Full models with Newey-West standard errors.

**Parameter**	**Coefficient**	**Lower 95% CI**	**Upper 95% CI**	***P*-value**
Change in Oxford hip score (score at 6 months – score at baseline)				
18-59 years old				
Intercept	17.781	17.368	18.193	<0.001
Monthly trend	0.097	0.040	0.154	0.001
Level change $ERAS_0$	0.867	0.439	1.295	<0.001
Trend change after $ERAS_0$	-0.049	-0.110	0.011	0.110
Level change $ERAS_{end}$	0.384	0.070	0.698	0.017
Trend change after $ERAS_{end}$	-0.027	-0.045	-0.010	0.003
60-69 years old				
Intercept	15.451	14.644	16.257	<0.001
Monthly trend	0.342	0.235	0.450	<0.001
Level change $ERAS_0$	0.578	-0.102	1.259	0.095
Trend change after $ERAS_0$	-0.303	-0.406	-0.200	<0.001
Level change $ERAS_{end}$	0.737	0.173	1.300	0.011
Trend change after $ERAS_{end}$	-0.035	-0.057	-0.013	0.002
70-79 years old				
Intercept	18.645	18.473	18.817	<0.001
Monthly trend	-0.047	-0.068	-0.026	<0.001
Level change $ERAS_0$	1.264	0.977	1.552	<0.001

Garriga C, *et al. BMJ Open* 2019; 9:e031599. doi: 10.1136/bmjopen-2019-031599

Trend change after $ERAS_0$	0.068	0.040	0.096	<0.001
Level change $ERAS_{end}$	0.490	0.106	0.874	0.013
Trend change after $ERAS_{end}$	0.000	-0.018	0.017	0.967
80-84 years old				
Intercept	15.207	14.066	16.349	<0.001
Monthly trend	0.364	0.217	0.511	<0.001
Level change $ERAS_0$	-0.060	-0.965	0.844	0.895
Trend change after $ERAS_0$	-0.374	-0.522	-0.226	<0.001
Level change $ERAS_{end}$	0.537	0.015	1.060	0.044
Trend change after $ERAS_{end}$	0.033	0.008	0.057	0.011
≥85 years old				
Intercept	16.187	9.205	23.168	<0.001
Monthly trend	0.487	-0.395	1.368	0.276
Level change $ERAS_0$	-1.708	-5.891	2.474	0.420
Trend change after $ERAS_0$	-0.503	-1.386	0.380	0.261
Level change $ERAS_{end}$	1.014	0.495	1.532	<0.001
Trend change after $ERAS_{end}$	0.024	-0.005	0.053	0.099
Without comorbidities				
Intercept	17.106	16.938	17.274	<0.001
Monthly trend	0.133	0.105	0.160	<0.001
Level change $ERAS_0$	1.187	0.967	1.406	<0.001
Trend change after $ERAS_0$	-0.109	-0.137	-0.080	<0.001
Level change $ERAS_{end}$	0.698	0.313	1.083	0.001
Trend change after $ERAS_{end}$	-0.010	-0.021	0.001	0.083
1+ comorbidities				

Garriga C, *et al. BMJ Open* 2019; 9:e031599. doi: 10.1136/bmjopen-2019-031599

Intercept	16.642	16.000	17.285	<0.001
Monthly trend	0.334	0.260	0.408	<0.001
Level change $ERAS_0$	-1.640	-2.136	-1.144	<0.001
Trend change after $ERAS_0$	-0.284	-0.362	-0.206	<0.001
Level change $ERAS_{end}$	0.097	-0.279	0.472	0.610
Trend change after $ERAS_{end}$	-0.030	-0.054	-0.006	0.013

Oxford hip score, OHS; confidence intervals, CI; Enhanced Recovery After Surgery, ERAS; start point of ERAS intervention in April 2009, $ERAS_0$; end point of ERAS intervention in March 2011, $ERAS_{end}$.

Garriga C, *et al. BMJ Open* 2019; 9:e031599. doi: 10.1136/bmjopen-2019-031599
