# Supplementary material for: Assessment on patient outcomes of primary hip replacement: an interrupted time series analysis from ‘The National Joint Registry of England and Wales’
Source: BMJ Open. 2019 Nov 21;9(11):e031599. doi: 10.1136/bmjopen-2019-031599 (PMC6887059; doi:10.1136/bmjopen-2019-031599)
Supplement: Author's manuscript [file bmjopen-2019-031599.draft_revisions.pdf]

BMJ Open is committed to open peer review. As part of this commitment we make the peer review history of every article we publish publicly available.

When an article is published we post the peer reviewers' comments and the authors' responses online. We also post the versions of the paper that were used during peer review. These are the versions that the peer review comments apply to.

The versions of the paper that follow are the versions that were submitted during the peer review process. They are not the versions of record or the final published versions. They should not be cited or distributed as the published version of this manuscript.

BMJ Open is an open access journal and the full, final, typeset and author-corrected version of record of the manuscript is available on our site with no access controls, subscription charges or pay-per-view fees (http://bmjopen.bmj.com).

If you have any questions on BMJ Open's open peer review process please email info.bmjopen@bmj.com

BMJ Open

# Assessment on Patient Outcomes of Primary Hip Replacement: an Interrupted Time Series Analysis from "The National Joint Registry of England and Wales"

| | |
|---|---|
| Journal: | *BMJ Open* |
| Manuscript ID | bmjopen-2019-031599 |
| Article Type: | Research |
| Date Submitted by the Author: | 15-May-2019 |
| Complete List of Authors: | Garriga, Cesar; University of Oxford, Nuffield Department of Orthopaedics, Rheumatology and Musculoskeletal Sciences (NDORMS); University of Oxford, Centre for Statistics in Medicine, Nuffield Department of Orthopaedics, Rheumatology and Musculoskeletal Sciences<br>Murphy, Jacqueline; University of Oxford, Nuffield Department of Orthopaedics, Rheumatology and Musculoskeletal Sciences (NDORMS); University of Oxford, Nuffield Department of Population Health<br>Leal, Jose; University of Oxford, Nuffield Department of Population Health<br>Arden, Nigel; University of Oxford, Nuffield Department of Orthopaedics, Rheumatology and Musculoskeletal Sciences (NDORMS); University of Southampton, MRC Lifecourse Epidemiology Unit<br>Price, Andrew; University of Oxford, Nuffield Department of Orthopaedics, Rheumatology and Musculoskeletal Sciences<br>Prieto-Alhambra, Daniel; University of Oxford Nuffield Department of Orthopaedics Rheumatology and Musculoskeletal Sciences; University of Oxford, Centre for Statistics in Medicine, Nuffield Department of Orthopaedics, Rheumatology and Musculoskeletal Sciences<br>Carr, Andrew; University of Oxford, Nuffield Department of Orthopaedics, Rheumatology and Musculoskeletal Sciences (NDORMS); University of Oxford, Centre for Statistics in Medicine, Nuffield Department of Orthopaedics, Rheumatology and Musculoskeletal Sciences<br>Rangan, Amar; University of Oxford, Nuffield Department of Orthopaedics, Rheumatology and Musculoskeletal Sciences (NDORMS); University of York, Department of Health Sciences<br>Cooper, Cyrus; University of Oxford, Nuffield Department of Orthopaedics, Rheumatology and Musculoskeletal Sciences (NDORMS); University of Southampton, MRC Lifecourse Epidemiology Unit<br>Peat, George; Keele University, Primary Care & Health Sciences<br>Fitzpatrick, Ray; University of Oxford, Nuffield Department of Population Health<br>Barker, Karen L.; University of Oxford, Nuffield Department of Orthopaedics, Rheumatology and Musculoskeletal Sciences (NDORMS); Oxford University Hospitals NHS Foundation Trust Nuffield Orthopaedic Centre<br>Judge, Andrew; University of Oxford, Nuffield Department of Orthopaedics, Rheumatology and Musculoskeletal Sciences (NDORMS); |

|  | University of Bristol, Musculoskeletal Research Unit, Translational Health Sciences, Bristol Medical School |
|---|---|
| Keywords: | Hip < ORTHOPAEDIC & TRAUMA SURGERY, EPIDEMIOLOGY, HEALTH ECONOMICS, HEALTH SERVICES ADMINISTRATION & MANAGEMENT |
|  |  |

SCHOLARONE™
Manuscripts

**Title:** Assessment on Patient Outcomes of Primary Hip Replacement: an Interrupted Time Series Analysis from "The National Joint Registry of England and Wales"

**Author names:** Cesar Garriga[1,2], Postdoctoral researcher (PhD, cesar.garriga-fuentes@ndorms.ox.ac.uk), Jacqueline Murphy[1,3], Researcher (MMath, jacqueline.murphy@qmul.ac.uk), Jose Leal[3], University Research Lecturer (DPhil, jose.leal@dph.ox.ac.uk), Nigel Arden[1,4], Professor in Rheumatic Diseases (Prof, nigel.arden@ndorms.ox.ac.uk), Andrew Price[1], Professor of Orthopaedic Surgery (Prof, andrew.price@ndorms.ox.ac.uk), Daniel Prieto-Alhambra[1,2,4], Professor of Pharmaco- and Device Epidemiology (Prof, daniel.prietoalhambra@ndorms.ox.ac.uk), Andrew Carr[1,2], Nuffield Professor of Orthopaedic Surgery (Prof, andrew.carr@ndorms.ox.ac.uk), Amar Rangan[1,5], Professor of Orthopaedic Surgery (Prof, Amar.Rangan@york.ac.uk), Cyrus Cooper[1,4], Professor of Rheumatology (Prof, cc@mrc.soton.ac.uk), George Peat[6], Professor of Clinical Epidemiology (Prof, g.m.peat@keele.ac.uk), Raymond Fitzpatrick[3], Professor of Public Health and Primary Care (Prof, raymond.fitzpatrick@nuffield.ox.ac.uk), Karen Barker[1,7], Professor of Physiotherapy (Prof, karen.barker@ouh.nhs.uk), Andy Judge[1,2,4,8], Professor of Translational Statistics (Prof, andrew.judge@ndorms.ox.ac.uk)

[1] Nuffield Department of Orthopaedics, Rheumatology and Musculoskeletal Sciences, Nuffield Orthopaedic Centre, University of Oxford, Windmill Road, Oxford, OX3 7LD, UK.

[2] Centre for Statistics in Medicine, Nuffield Department of Orthopaedics, Rheumatology and Musculoskeletal Sciences, Nuffield Orthopaedic Centre, University of Oxford, Windmill Road, Oxford, OX3 7LD, UK.

[3] Nuffield Department of Population Health, University of Oxford, Richard Doll Building, Old Road Campus, Oxford, OX3 7LF, UK.

For peer review only

[4] MRC Lifecourse Epidemiology Unit, University of Southampton, Southampton General Hospital, Southampton, SO16 6YD, UK.

[5] Department of Health Sciences, University of York, Seebohm Rowntree Building, Heslington, York YO10 5DD

[6] Primary Care & Health Sciences, Keele University, Keele, Staffordshire ST5 5BG, UK.

[7] Physiotherapy Research Unit, Nuffield Orthopaedic Centre, Windmill Rd, Headington, Oxford, OX3 7HE, UK.

[8] Musculoskeletal Research Unit, Translational Health Sciences, Bristol Medical School, University of Bristol, Learning and Research Building, Level 1, Southmead Hospital, Bristol, BS10 5NB, UK.

**Corresponding Author**: Cesar Garriga; Botnar Research Centre; Nuffield Department of Orthopaedics, Rheumatology and Musculoskeletal Sciences; University of Oxford; Windmill Road, Headington; Oxford, OX3 7LD, UK; Tel. +441865 737859; F +44 (0)1865 227 966.

Email: cesar.garriga-fuentes@ndorms.ox.ac.uk.

For peer review only

## STRUCTURED ABSTRACT

**Objectives**: Effects of the UK Department of Health's national Enhanced Recovery After Surgery (ERAS) Programme on outcomes after primary hip replacement.

**Design:** Natural experimental study using interrupted time-series to assess the changes in trends before, during, and after ERAS implementation (April 2009 to March 2011).

**Setting:** Surgeries in the UK National Joint Registry were linked with Hospital Episode Statistics containing inpatient episodes from NHS trusts in England and patient-reported outcome measures.

**Participants**: Patients aged ≥18 years from 2008 to 2016.

**Main outcome measures:** Regression coefficients of monthly means of length of hospital stay, bed day cost, change in Oxford hip scores (OHS) 6 months post-surgery, complications 6 months post-surgery, and revision rates 5 years post-surgery.

**Results:** 438,921 primary hip replacements were identified. Hospital stays shortened from 5.6 days in April 2008 to 3.6 in December 2016. There were also improvements in bed day costs (£7573 in April 2008 to £5239 in December 2016), positive change in self-reported OHS from baseline to 6 months post-surgery (17.7 points in April 2008 to 22.9 points in December 2016), complication rates (4.1 % in April 2008 to 1.7 % March 2016), and 5-year revision rates (5.9 per 1000 implant-years (95% CI: 4.8 to 7.2) in April 2008 to 2.9 (95% CI:

For peer review only



For peer review only - http://bmjopen.bmj.com/site/about/guidelines.xhtml

2.2 to 3.9) in December 2011). The positive trends in all outcomes started before ERAS was implemented and continued during and after the programme**.**

**Conclusions:** Patient outcomes after hip replacement have improved over the last decade. A national ERAS programme maintained this improvement but did not alter the existing rate of change.

For peer review only

**Strengths and limitations of this study**

- Our study design is a "natural experiment" which controlled for unobservable sources of confounding. This approach for evaluating complex interventions allows for strong causal inferences without randomised controlled experiments.
- Routinely collected data provided actual-practice information on trends in length of stay, patient reported outcome measures of hip pain and function, complications, and revision surgeries following primary hip replacement procedures.
- A limitation is the variation in interpretation and adoption across centres because what constitutes enhanced recovery after surgery (ERAS) was not clearly established after the expected identification of best practices in the first year of the ERAS programme.



For peer review only - http://bmjopen.bmj.com/site/about/guidelines.xhtml

## INTRODUCTION

Osteoarthritis represents a significant, and growing, population health burden worldwide(1). In the United Kingdom (UK), over one million adults aged over 45 years consult general practice for osteoarthritis each year(2). Osteoarthritis accounts for £3.2 billion in lost productivity in the UK(3), with total direct and indirect costs equivalent to 0.25-0.50% of the gross domestic product(4, 5). Almost 88,000 primary hip replacement operations were undertaken during 2016 in the National Health Service (NHS), over 90% of which were for osteoarthritis. This number continues to increase(6).

Between April 2009 and March 2011, the UK Department of Health implemented an Enhanced Recovery After Surgery (ERAS) Partnership Programme(7) to improve recovery in major planned colorectal, musculoskeletal, gynaecological, and urological surgical pathways. The first year of the programme focused on learning best practice from pioneer units of ERAS practice in the NHS. It collected information about principles of enhanced recovery, clinical elements of the patient pathway, metrics, and success factors. It established a website to share information and resources, generated a financial and equality impact evaluation, published an implementation guide, and developed an online reporting tool to support implementation. A lead for enhanced recovery was named in each local health authority to prepare for the spread of the ERAS programme across the NHS during the implementation phase in the programme’s second year.

The ERAS in musculoskeletal care focused on planned hip and knee replacement surgeries. ERAS is a complex intervention(8, 9) to improve several areas of care across a patient’s pathway through surgery. Pre-operatively, ERAS aims for the patient to be in the best possible condition for surgery. Peri-operatively, ERAS aims to give the patient the best



For peer review only - http://bmjopen.bmj.com/site/about/guidelines.xhtml

possible management during and after surgery(10). Post-operatively, ERAS aims to give the patient the best possible rehabilitation. Patients are given information before and after surgery, such as changes to make around the home, strengthening exercises, and changes to nutrition. ERAS aims to enable earlier return home from hospital with tailored discharge, when suitable for the patient.

Despite their widespread implementation, there is limited evidence about the effectiveness of ERAS programmes in hip and knee replacement surgery(11). We aimed to determine whether implementing ERAS in hip replacement has led to improved patient outcomes and shorter hospital stays.

## METHODS

### Data source

We used data from the UK National Joint Registry (NJR), which contains data on hip replacement surgeries from all English and Welsh hospitals. It includes 2 million patients since 2003 and covers 95% and 91% of primary hip replacements and revisions, respectively(12).

### Data linkages

Primary operations were linked with Hospital Episode Statistics (HES) data, which contain records of all inpatient episodes undertaken in NHS trusts in England (125 million each year). Planned hip replacements were linked to patient-reported outcome measures (PROMs). Patients funded by the NHS in England are asked to complete questionnaires before and 6 months after surgery to evaluate their perceived improvement in health. We retrieved a cohort

For peer review only

of patients undergoing planned hip replacement in England between April 2008 and December 2016. Mortality data were matched to the Office for National Statistics database.

**Outcome measures**

We evaluated trends in hospital stay length for patients undergoing primary hip replacement. Length of stay was calculated as the number of days between the hospital admission and discharge dates. We used the same set of patients to estimate the inpatient cost for the index episode using NHS reference costs from 2015/16(13). We estimated the mean cost per bed day based on each patient's healthcare resource use and length of hospital stay. Appendix 1 further explains the cost methods.

We also assessed the absolute change in the Oxford hip score (OHS), a PROM. Patients complete the same questionnaire about their hip pain and function before and 6 months after surgery(14). Each question is scored between 0 (worst symptoms) and 4 (least symptoms). The scores from the 12 questions are summed to give a total score between 0 (worst) and 48 (best). We calculated the difference between the total scores 6 months post-surgery and at baseline to obtain a measure of change associated with surgery. Higher positive values for OHS change represent greater self-reported improvement in pain and function.

We calculated the 6 months post-surgery complication proportions. We defined post-operative complications as one or more events happening up to 6 months after primary hip replacement: stroke (excluding transient ischaemic attack), respiratory infection, acute myocardial infarction, pulmonary embolism/deep vein thrombosis, urinary tract infection, wound disruption, surgical site infection, fracture after implant, complication of prosthesis, neurovascular injury, acute renal failure, or blood transfusion. We identified these

For peer review only

complications in HES data using diagnosis codes from the "International Statistical Classification of Diseases and Related Health Problems 10th Revision" (ICD-10) (Appendix 2), except for blood transfusion, for which we used the "Classification of Interventions and Procedures version 4" (OPCS-4) codes (Appendix 3).

We evaluated the rate of revision surgery up to 5 years after primary hip replacement. We included revisions declared to the NJR registry by surgeons(15) and reported to HES using codes from Appendix 4. We specified our analysis time in years, reporting the rate as the number of revisions per 1000 implant-years.

**Intervention**

The ERAS programme for hip replacement surgery was implemented nationally between April 2009 and March 2011(16).

**Potential modifiers**

We evaluated whether trends in hospital stay length and OHS change after surgery differed by age at the time of primary hip replacement (18-59, 60-69, 70-79, 80-84, or ≥85 years) and presence of co-morbidities according to the Charlson classification(17) (no comorbidities versus one or more comorbidities): myocardial infarction, congestive heart failure, peripheral vascular disease, cerebrovascular disease, dementia, chronic obstructive pulmonary disease, connective tissue disease, peptic ulcer disease, mild liver disease, mild diabetes, hemiplegia, moderate/severe renal disease, severe diabetes (i.e., with organ damage), tumour, leukaemia, lymphoma, moderate/severe liver disease, AIDS, and metastatic solid tumour. The ICD-10 codes used are listed in Appendix 5.



For peer review only - http://bmjopen.bmj.com/site/about/guidelines.xhtml

**Participants and inclusion criteria**

We included patients receiving planned hip replacement surgery (Fig. 1) between 1 April 2008 and 31 December 2016. We excluded patients without a concordant date of replacement between NJR and HES registries.

When analysing length of stay, we also excluded patients staying more than 15 days in hospital and patients with a missing hospital stay length or a hospital discharge date before their hospital admission date. When analysing change in OHS, we also excluded patients missing baseline and/or 6-month follow-up OHS scores. When analysing complications, we also excluded patients with complications before the surgery date and those with surgery after June 2016 to guarantee all patients had at least 6 months of follow-up. When analysing 5-year revision rates, we excluded patients who received surgery after 2011 to ensure all patients had at least 5 years of follow-up.

**Missing data**

We excluded patients with missing length of hospital stay or missing OHS data from the respective analyses. We used Pearson's $\chi^2$ statistic to compare missingness in the OHS measure before, during, and after ERAS implementation (April 2008 - March 2009, April 2009 - March 2011, and April 2011 - December 2016), across patient age (18-59, 60-69, 70-79, 80-84, and ≥85 years), and in the presence and absence of co-morbidities. We also compared the distribution of patients with and without OHS data by study period, patient age, and presence of co-morbidities.

**Study design and statistical analysis**



For peer review only - http://bmjopen.bmj.com/site/about/guidelines.xhtml

We used a natural experimental study design(18). We evaluated the effect of ERAS on trends before, during, and after its implementation (19, 20). Trusts could choose when to start implementing the programme within the implementation period (April 2009 to March 2011), and we assumed that they only implemented the programme within this period. We described the trends by calculating monthly outcomes as means (length of stay, bed costs, change in OHS), proportions (complications), or rates (revision), and their 95% confidence intervals (CI). We estimated a fractional polynomial over the study period and plotted the resulting curve with the CI of the mean.

We used an interrupted time series approach to estimate changes in outcomes during and immediately after the intervention period, while controlling for baseline levels and trends. We modelled aggregated data points of each outcome of interest by month using segmented linear regression(20):

$$Y_t = \beta_0 + (\beta_1 * \text{time}_t) + (\beta_2 * \text{ERAS}_0) + (\beta_3 * \text{time after ERAS}_0) + (\beta_4 * \text{ERAS}_{end}) + (\beta_5 * \text{time after ERAS}_{end}) + e_t.$$

Yt is the mean of each outcome for patients undergoing primary hip replacements – mean days in hospital for length of stay, mean OHS change for the PROM analysis, mean proportion of complications for 6-month complications outcome, and mean rate of revisions of primary hip replacements for the 5-year revision outcome – in month t. “Time” is a continuous variable representing the number of months from the start of the observation period (April 2008) until time t. $\beta_0$ estimates the baseline level of the outcome at the beginning of the time series (April 2008). $\beta_1$ estimates the trend before ERAS was implemented in April 2009. $\beta_2$ is the change in level immediately after the intervention



For peer review only - http://bmjopen.bmj.com/site/about/guidelines.xhtml

($ERAS_0$ = April 2009). $\beta_3$ estimates the change in the trend in the monthly mean (number or rate, depending on outcome) after ERAS started (i.e., ERAS implementation trend). $\beta_4$ is the change in level immediately after the end of the intervention ($ERAS_{end}$ = March 2011). $\beta_5$ estimates the change in the trend in the mean monthly number or rate (depending on outcome) after ERAS ended.

We excluded non-significant terms using a backward approach to maximise statistical power, producing a parsimonious model with meaningful selected variables. In preliminary analysis we checked for autocorrelation with the previous month, two months…twelve months using Durbin's alternative test(21). As autocorrelation invalidated the interpretation of the model, we estimated the linear regression models with Newey-West standard errors(22). Parsimonious models were generated using the variables previously selected in the backward regression. We also report the full models.

All analyses were conducted using Stata version 13.1 statistical software (StataCorp, College Station, Texas). We followed the STROBE (Strengthening the Reporting of Observational Studies in Epidemiology) guideline in reporting this study(23).

**Patient and public involvement**

The James Lind Alliance has identified the need for involving patients in identifying outcomes that matter to them (patient-identified outcomes). We conducted a patient forum with the PEP-R group to identify outcomes from those available in the routine datasets available for this study. The top outcomes were: 1) pain and function, 2) complications (particularly hospital-acquired infection), 3) length of stay (dependent on the level of support

For peer review only



For peer review only - http://bmjopen.bmj.com/site/about/guidelines.xhtml

at home), 4) revision surgery, and 5) mortality (rated low importance) (further detail is provided in Supplementary Text S1).

## RESULTS

We identified 438,921 planned primary hip replacements between April 2008 and December 2016 (Fig. 1). Sixty percent of patients were women and the average age was 69 years (SD ±11 years). The mean body mass index (BMI) at primary surgery of 28.9 kg/m$^2$ (SD ±5.2 kg/m$^2$) fell into the overweight category (24), although BMI was missing for 28% of patients. Most patients (84%) had a physical status(25) of "mild" or "fit".

### Length of stay

Hospital stays shortened from 5.6 days (95% CI: 5.5 to 5.7) in April 2008 to 3.6 (95% CI: 3.6 to 3.7) in December 2016 (Fig. 2A). As shown in Table 1, they were already shortening significantly by -0.020% every month (95% CI: -0.023% to -0.017%) before ERAS (full models in Supplementary Table S1). Hospital stay shortened more quickly (-0.033%) during the implementation period (April 2009 to March 2011), then declined at a slower rate (-0.002%) once ERAS ended (April 2011 to December 2016).

Although older patients had longer hospital stays, all age groups shared the shortening stay trend (Fig. 3, Supplementary Tables S2 and S3). For example, those aged 18-59 years decreased from 4.7 days (95% CI: 4.6 to 4.9) in April 2008 to 3.0 days (95% CI: 2.9 to 3.1) in December 2016 and those aged ≥85 years decreased from 8.1 days (95% CI: 7.7 to 8.6) in April 2008 to 5.3 days (95% CI: 4.9 to 5.6) in December 2016. Patients with and without pre-existing co-morbidity also had shortening hospital stays (Fig. 4).



For peer review only - http://bmjopen.bmj.com/site/about/guidelines.xhtml

We estimated cost data for 432,143 patients. The mean inpatient bed day cost over time showed a similar trend to that observed for hospital stay length. The overall mean cost of the index hospital episode decreased from £7573 (95% CI: £7477 to £7668) in April 2008 to £5239 (95% CI: £5171 to £5306) in December 2016 (Supplementary Fig. S1).

**OHS change**

We excluded 48% of patients with missing OHS information from the change in OHS analysis (Fig. 1). We found more missing OHS data before the intervention (89.7%) than during (41.9%) or after (45.0%) the intervention (Supplementary Table S4). Supplementary Table S5 shows more patients without OHS change data than with this data in the period prior to ERAS (16.2% and 1.7%, respectively).

Self-reported OHS scores improved over the study period, with an increase in OHS 6 months after surgery of 17.7 points (95% CI: 16.4 to 19.0) in April 2008, to 22.9 points (95% CI: 21.8 to 23.9) in December 2016 (Fig. 2B). This trend was also seen in patients with and without co-morbidities and in all age groups, except in those aged ≥85 years, whose change in OHS remained stable over the study period (Supplementary Figures S2 and S3, Supplementary Tables S6 and S7).

According to the interrupted time-series model (Table 1), OHS change increased significantly by 0.158% (95% CI: 0.130% to 0.186%) every month before the intervention (see Supplementary Table S1 for the full model). During ERAS implementation (April 2009 to March 2011), the increase continued but slowed to 0.027%. Change in OHS then became stable after ERAS implementation ended (April 2011 to December 2016).



For peer review only - http://bmjopen.bmj.com/site/about/guidelines.xhtml

### Complications at 6 months

6,232 (1.6%) patients with a primary hip replacement between April 2008 and March 2016 had one or more complication in the 6 months after surgery. The proportion of complications at 6 months decreased from 4.1% (95% CI: 3.4 to 4.7) to 1.7% (95% CI: 1.3 to 2.1) over the study period (Fig. 2C). According to the interrupted time-series model, complications at 6 months decreased by -0.078% (95% CI: -0.096% to -0.071%) every month before the intervention (Table 1). During the intervention, the trend reversed and complications increased by 0.078%. The proportion of complications stabilised after the intervention ended.

### Five-year revision rates

According to the NJR registry, 3,392 (2.1%) patients with a primary hip replacement between April 2008 and December 2011 had a hip revision in the following 5 years. We found 840 more 5-year revisions using HES, giving a total of 4,232 (2.6%). Rates of 5-year hip revision decreased from 7.6 per 1000 implant-years (95% CI: 6.4 to 9.2) at risk in April 2008 to 3.8 (95% CI: 3.0 to 4.9) in December 2011 (Fig. 2D).

According to a parsimonious model of 5-year hip-revision rates (Table 1), there was a significant downward trend of -0.098 per 1000 implant-years (95% CI: -0.108 to -0.087) before the implementation of the intervention (April 2009 to March 2011) (see Supplementary Table S1 for the full model). The trend reversed after the intervention ended (April 2011 to December 2016) to increase 0.091 per 1000 implant-years (95% CI: 0.052 to 0.129).

## DISCUSSION



For peer review only - http://bmjopen.bmj.com/site/about/guidelines.xhtml

We have shown that primary hip replacement outcomes have been improving since 2008, with decreasing length of hospital stay, estimated average inpatient bed day costs, complications, and 5-year revision risk, and improving patient-reported pain and function. These positive trends were seen across all age groups and in those with and without co-morbidity, and began before the NHS ERAS programme was implemented. Hospital stays have shortened without adversely affecting patient outcomes.

We hypothesised that the national ERAS intervention would improve primary hip replacement outcomes by changing trends during and after its implementation. Our hypothesis was not confirmed, as ERAS did not influence existing trends. However, we collected only one year of data before the intervention was implemented (April 2008 to March 2009), in comparison with two years of data during implementation (April 2009 to March 2011) and five years of data post-intervention (April 2011 to December 2016). It is unclear how the outcomes varied before April 2008.

Although our study design controlled for unobservable sources of confounding, we stratified by age and presence/absence of comorbidities at surgery to detect patterns in outcome variation. This approach for evaluating complex interventions allows for strong causal inferences without randomised controlled experiments (9, 19).

For this "natural experiment," we assumed that ERAS was implemented homogenously across all England NHS trusts in the 2-year implementation period. This assumption is unlikely to be true. The shortening hospital stays and improved outcomes seen before April 2009 may reflect some trusts implementing ERAS elements before the national programme began. Shortening hospital stays before ERAS may also reflect attempts to improve the cost-



For peer review only - http://bmjopen.bmj.com/site/about/guidelines.xhtml

effectiveness of hip replacement surgery, which is an important expenditure for the NHS (26-28).

The hospitals implemented ERAS on different dates, and some had not implemented ERAS by March 2011(16). The Department of Health surveyed trusts on their use of ERAS near the end of the implementation period (February 2011) and reported full implementation by 81 consultant teams, partial implementation by about 20 teams, and plans to implement ERAS by about 30 teams. As the Department of Health did not clearly describe ERAS after best practices were identified in the first year of the programme(29), there is also variation in the teams' interpretation and adoption of the programme. The Department of Health guideline did not indicate how long ERAS should be implemented for and its later report did not measure actual implementation periods in each hospital(16, 29). Considering the complexity of the intervention and stakeholders involved, implementation length is likely to have varied between hospitals. Our quasi-experimental approach smooths the potential dissimilarities.

For peer review only

Existing systematic reviews and randomised clinical trials have found that ERAS programmes for planned colorectal, joint replacement, and other surgeries reduced hospital stays when compared with conventional care(11). Many kinds of ERAS programmes for hip replacement have been investigated: physical therapy the same day of the surgery in the recovery room(30); preoperative patient education, postoperative multimodal analgesia with periarticular injections, early physiotherapy and rehabilitation, and discharge home with an outpatient rehabilitation programme(31, 32); patient and staff education on "enhanced recovery" principles, pre-admission medication, perioperative urinary catheterisation, low-



For peer review only - http://bmjopen.bmj.com/site/about/guidelines.xhtml

dose spinal anaesthesia, and aiming for same-day mobilisation(33); perioperative information, pain relief, nausea control, nutrition, mobilisation, and elimination(34); preoperative patient seminar, treatment of pain (spinal anaesthesia) and early mobilisation, standardised programme in the operating theatre (tranexamic acid and no drains), 1-2 hours of a multimodal fast-track rehabilitation regime, daily physiotherapy within the first 24 hours, and a multimodal oral opioid-sparing analgesia(35); or a perioperative analgesic blocking the peripheral nerve(36). However, these studies involved small sample sizes (170, 57, 1256, 630, 28, 98, 15)(30-36), were limited to one hospital or trust, and only compared their intervention with traditional management. They cannot be generalised to the wider population. We investigated whether the ERAS for hip replacement was successfully implemented by comparing with a pre-ERAS period, as was done in other studies(31-33), but for the first time, by also comparing with the post-intervention period. We also included all of the hospitals in one country.

Shorter hospital stays were reflected in lower estimated average inpatient bed day costs. Most surgery episodes in the dataset had a hospital stay shorter than the trim point for the cost of the relevant healthcare resource use group. A drop in hospital stay length within the trim point would not be reflected by a change in the estimated average episode costs, if we assign the same unit cost to all patients in the same healthcare resource use group who had a length of stay shorter than the trim point. We therefore estimated the true reduction in NHS expenditure by estimating a cost per bed day reflecting each patient's hospital stay length.

OHS change scores increased across the study period: the difference between pain and function at baseline and 6 months post-surgery was greater at the end of 2016 than in 2008, indicating less pain and better function after surgery. A review of ERAS in total hip



For peer review only - http://bmjopen.bmj.com/site/about/guidelines.xhtml

replacement showed that better improvement in pain and function scores could be related to making patients active participants in their recovery and managing patient expectations(27). A Cochrane review on preoperative education for hip or knee replacement do not find benefits over usual care, but did find a non-significant reduction in pain and better function associated with perioperative education(37).

Complications 6 months after surgery were decreasing before ERAS was implemented, remained steady during the ERAS period, and increased after the intervention ended. Discharging patients too soon after surgery could increase complications. A meta-analysis of ERAS programmes for colorectal surgery did not find evidence of increased surgical site infections or anastomotic leakage, classified as surgical complications (relative risk = 0.76, 95 % CI 0.54–1.08), and found that cardiovascular, pulmonary, and infectious medical complications decreased (relative risk = 0.40, 95 % CI 0.27–0.61).(38) Patients with diabetes undergoing hip and knee replacement under ERAS protocols had a lower additional risk for complications otherwise associated with operating on patients with diabetes(39).

The 5-year revision surgery rates dropped over the study period, a desirable finding as the revision procedure is more complicated than the initial procedure(40). Revision rates may have declined due to the UK National Institute for Health and Care Excellence (NICE) recommendation to only use implants with a 10-year revision rate of 5% or lower, to avoid low-quality prostheses(26).

The promising improvements in primary hip replacement outcomes reported here were achieved amidst increasingly strained NHS funding and hospital budgets. NHS funding growth in our study period was slower than historical trends(41). There are fewer hospital



For peer review only - http://bmjopen.bmj.com/site/about/guidelines.xhtml

beds available for hip replacement today than at the beginning of the study period, and wards have been closed. For example, there was an average daily 10,015 occupied beds open overnight for trauma and orthopaedics in England between April and June 2010, dropping to 8,770 between October and December 2016(42). Conversely, the number of primary hip replacements in England increased from 67,128 in 2008 to 87,733 in 2016(6). It has been estimated that 97,516 total hip replacements will take place in 2035(43). Meeting this demand with existing or lower capacity will require efficiencies.

An important issue is the high variation in services and practices across English hospitals. The Getting It Right First Time (GIRFT) programme aims to reduce discrepancies in activity volume, implant choice, and guideline follow-up between hospitals(44). Despite national improvements in hip replacement outcomes, GIRFT reports substantial variation in outcomes between hospital trusts. In 2016, the mean length of hospital stay after primary hip replacement varied between 2.5 and 11.6 days, and OHS change varied between 12.0 and 23.5 points. Although the national picture has improved for patients, there is still work remaining to understand and reduce unwarranted outcome variations between individual hospitals.

**Conclusion**

Our study shows that outcomes after planned hip replacement are better today than 10 years ago. Hospital stays shortened substantially from 2008 to 2016 for all age groups and in people with and without co-morbidity, without adversely affecting patient outcomes. Patient-reported pain and function have improved, revision rates are in decline, and complication rates remain stable. The introduction of a national ERAS programme maintained this improvement but did not alter the rate of change already underway.



For peer review only - http://bmjopen.bmj.com/site/about/guidelines.xhtml

For peer review only

**Acknowledgements**

We would like to thank the patients and staff of all the hospitals in England and Wales who have contributed data to the National Joint Registry (NJR); and the Healthcare Quality Improvement Partnership (HQIP), the NJR Steering Committee and staff at the NJR Centre for facilitating this work. The authors have conformed to the NJR's standard protocol for data access and publication.

**Contributors**

All authors contributed to study design, data analysis, interpretation of results and writing the manuscript. All authors had full access to all statistical reports and tables in the study. CG had full access to all of the study data and takes responsibility for the integrity of the data and the accuracy of the data analysis. All authors contributed to the interpretation of results and critical revision of the manuscript and approved the final manuscript. AJ is the guarantor. The corresponding author attests that all listed authors meet authorship criteria and that no others meeting the criteria have been omitted.

**Competing interests**

All authors have completed the Unified Competing Interest form at www.icmje.org/coi_disclosure.pdf and declare: AJ has received consultancy fees from Anthera Pharmaceuticals, Inc., Servier, the UK Renal Registry, and the Oxford Craniofacial Unit, and has received a research grant from Roche. AP reports personal fees from Zimmer Biomet, outside the submitted work. AR reports grants from DePuy Ltd outside the submitted work. CC received personal fees from: Alliance for Better Bone Health, Amgen, Eli Lilly, GSK, Medtronic, Merck, Novartis, Pfizer, Roche, Servier, Takeda, and UCB outside the submitted work. NKA has received honorariums from Novartis, Alliance for Better Health,

For peer review only

and Lilly; held advisory board positions (which involved receipt of fees) at Merck, Merck Sharp and Dohme, Roche, Novartis, Smith and Nephew, Q-MED, Nicox, Servier, GlaxoSmithKline, Schering-Plough, Pfizer, and Rottapharm; and received consortium research grants from Alliance for Better Bone Health, Amgen, Novartis, Merck Sharp and Dohme, Servier, Eli Lilly, and GlaxoSmithKline; he has no other relationships or activities that could appear to have influenced the submitted work. All other authors declare no conflicts of interest.

**Funding**

This project was funded by the NIHR Health Services and Delivery Research programme (project number 14/46/02). Andrew Judge is supported by the NIHR Biomedical Research Centre at the University Hospitals Bristol NHS Foundation Trust and the University of Bristol. The views expressed in this publication are those of the authors and do not necessarily reflect those of the NHS, the National Institute for Health Research or the Department of Health and Social Care.

**Data sharing**

Access to data is available from the National Joint Registry for England and Wales, Northern Ireland and the Isle of Man, but restrictions apply to the availability of these data, which were used under license for the current study, and so are not publicly available. Data access applications can be made to the National Joint Registry Research Committee. Access to linked HES and PROMs data is available through data applications to NHS Digital.

**REFERENCES**

For peer review only



For peer review only - http://bmjopen.bmj.com/site/about/guidelines.xhtml

1. Global, regional, and national incidence, prevalence, and years lived with disability for 328 diseases and injuries for 195 countries, 1990–2016: a systematic analysis for the Global Burden of Disease Study 2016. The Lancet. 2017;390(10100):1211-59.

2. Jordan KP, Joud A, Bergknut C, Croft P, Edwards JJ, Peat G, et al. International comparisons of the consultation prevalence of musculoskeletal conditions using population-based healthcare data from England and Sweden. Annals of the rheumatic diseases. 2014;73(1):212-8.

3. Chen A, Gupte C, Akhtar K, Smith P, Cobb J. The Global Economic Cost of Osteoarthritis: How the UK Compares. Arthritis. 2012;2012:698709.

4. Hiligsmann M, Cooper C, Arden N, Boers M, Branco JC, Luisa Brandi M, et al. Health economics in the field of osteoarthritis: an expert's consensus paper from the European Society for Clinical and Economic Aspects of Osteoporosis and Osteoarthritis (ESCEO). Seminars in arthritis and rheumatism. 2013;43(3):303-13.

5. Puig-Junoy J, Ruiz Zamora A. Socio-economic costs of osteoarthritis: a systematic review of cost-of-illness studies. Seminars in arthritis and rheumatism. 2015;44(5):531-41.

6. National Joint Registry. 14th Annual Report of the UK NJR. London: National Joint Registry for England, Wales and Northern Ireland, 2017.; 2017.

7. NHS_Improving_Quality. Enhanced recovery care pathways: a better journey for patients seven days a week and a better deal for the NHS: NHS England; 2013.

8. Campbell M, Fitzpatrick R, Haines A, Kinmonth AL, Sandercock P, Spiegelhalter D, et al. Framework for design and evaluation of complex interventions to improve health. BMJ (Clinical research ed). 2000;321(7262):694-6.

9. Craig P, Dieppe P, Macintyre S, Michie S, Nazareth I, Petticrew M. Developing and evaluating complex interventions: the new Medical Research Council guidance. BMJ (Clinical research ed). 2008;337:a1655.



For peer review only - http://bmjopen.bmj.com/site/about/guidelines.xhtml

10. Wilmore DW, Kehlet H. Management of patients in fast track surgery. BMJ (Clinical research ed). 2001;322(7284):473-6.

11. Paton F, Chambers D, Wilson P, Eastwood A, Craig D, Fox D, et al. Effectiveness and implementation of enhanced recovery after surgery programmes: a rapid evidence synthesis. BMJ open. 2014;4(7):e005015.

12. NJR-Editorial-Board. 14th Annual Report: National Joint Registry for England, Wales, Northern Ireland and the Isle of Man. http://www.njrreports.org.uk/Portals/0/PDFdownloads/NJR%2014th%20Annual%20Report%202017.pdf 2017.

13. Deparment_of_Health. Reference Costs 2015-16; 2016.

14. A Guide to PROMs Methodology. 4 ed: Health and Social Care Information Centre. http://content.digital.nhs.uk/media/1537/A-Guide-to-PROMs-Methodology/pdf/PROMs_Guide_V12.pdf; 2017. p. 42 pp.

15. National-Joint-Registry. Data collection forms - revision procedure for hip. http://www.njrcentre.org.uk/njrcentre/Portals/0/Documents/England/Data%20collection%20forms/MDSv6.0_H2_V004.pdf. 2014 [cited 04/12/2017]; Available from: http://www.njrcentre.org.uk/njrcentre/Healthcareproviders/Collectingdata/Datacollectionforms/tabid/103/Default.aspx

16. Deparment_of_Health. Enhanced recovery Partnership Project Report - March 2011: Department of health; 2011.

17. Charlson ME, Pompei P, Ales KL, MacKenzie CR. A new method of classifying prognostic comorbidity in longitudinal studies: development and validation. Journal of chronic diseases. 1987;40(5):373-83.



For peer review only - http://bmjopen.bmj.com/site/about/guidelines.xhtml

18. Craig P, Cooper C, Gunnell D, Haw S, Lawson K, Macintyre S, et al. Using natural experiments to evaluate population health interventions: new Medical Research Council guidance. Journal of Epidemiology and Community Health. 2012;66(12):1182-6.

19. Kontopantelis E, Doran T, Springate DA, Buchan I, Reeves D. Regression based quasi-experimental approach when randomisation is not an option: interrupted time series analysis. BMJ (Clinical research ed). 2015;350:h2750.

20. Wagner AK, Soumerai SB, Zhang F, Ross-Degnan D. Segmented regression analysis of interrupted time series studies in medication use research. Journal of clinical pharmacy and therapeutics. 2002;27(4):299-309.

21. Durbin J. Testing for Serial Correlation in Least-Squares Regression When Some of the Regressors are Lagged Dependent Variables. Econometrica. 1970;38(3):410-21.

22. Newey WK, West KD. A Simple, Positive Semi-Definite, Heteroskedasticity and Autocorrelation Consistent Covariance Matrix. Econometrica. 1987;55(3):703-8.

23. Vandenbroucke JP, Elm E, Altman DG, et al. Strengthening the reporting of observational studies in epidemiology (strobe): Explanation and elaboration. Annals of internal medicine. 2007;147(8):W-163-W-94.

24. World-Health-Organization. Obesity: preventing and managing the global epidemic. Geneva, Switzerland; 2000.

25. Lubin MF, Dodson TF, Winawer NH. Medical management of the surgical patient: a textbook of perioperative medicine: Cambridge University Press; 2013.

26. NICE-guidelines. Total hip replacement and resurfacing arthroplasty for end-stage arthritis of the hip. Technology appraisal guidance [TA304]; 2014.

27. Ibrahim MS, Twaij H, Giebaly DE, Nizam I, Haddad FS. Enhanced recovery in total hip replacement. A clinical review. 2013;95-B(12):1587-94.

For peer review only

28. Burn E, Edwards CJ, Murray DW, Silman A, Cooper C, Arden NK, et al. Trends and determinants of length of stay and hospital reimbursement following knee and hip replacement: evidence from linked primary care and NHS hospital records from 1997 to 2014. BMJ open. 2018;8(1):e019146.

29. Enhanced-Recovery-Partnership-Programme. Delivering enhanced recovery – Helping patients to get better sooner after surgery. In: Health Do, editor.; 2010.

30. Tayrose G, Newman D, Slover J, Jaffe F, Hunter T, Bosco J, 3rd. Rapid mobilization decreases length-of-stay in joint replacement patients. Bulletin of the Hospital for Joint Disease (2013). 2013;71(3):222-6.

31. Raphael M, Jaeger M, van Vlymen J. Easily adoptable total joint arthroplasty program allows discharge home in two days. Canadian journal of anaesthesia = Journal canadien d'anesthesie. 2011;58(10):902-10.

32. Khan SK, Malviya A, Muller SD, Carluke I, Partington PF, Emmerson KP, et al. Reduced short-term complications and mortality following Enhanced Recovery primary hip and knee arthroplasty: results from 6,000 consecutive procedures. Acta Orthopaedica. 2014;85(1):26-31.

33. Malviya A, Martin K, Harper I, Muller SD, Emmerson KP, Partington PF, et al. Enhanced recovery program for hip and knee replacement reduces death rate. Acta Orthopaedica. 2011;82(5):577-81.

34. Larsen K, Sorensen OG, Hansen TB, Thomsen PB, Soballe K. Accelerated perioperative care and rehabilitation intervention for hip and knee replacement is effective: a randomized clinical trial involving 87 patients with 3 months of follow-up. Acta Orthop. 2008;79(2):149-59.

35. Husted H, Lunn TH, Troelsen A, Gaarn-Larsen L, Kristensen BB, Kehlet H. Why still in hospital after fast-track hip and knee arthroplasty? Acta Orthopaedica. 2011;82(6):679-84.

For peer review only

36. Hebl JR, Dilger JA, Byer DE, Kopp SL, Stevens SR, Pagnano MW, et al. A pre-emptive multimodal pathway featuring peripheral nerve block improves perioperative outcomes after major orthopedic surgery. Regional anesthesia and pain medicine. 2008;33(6):510-7.

37. McDonald S, Page MJ, Beringer K, Wasiak J, Sprowson A. Preoperative education for hip or knee replacement. Cochrane Database of Systematic Reviews. 2014(5).

38. Greco M, Capretti G, Beretta L, Gemma M, Pecorelli N, Braga M. Enhanced Recovery Program in Colorectal Surgery: A Meta-analysis of Randomized Controlled Trials. World Journal of Surgery. 2014;38(6):1531-41.

39. Jorgensen CC, Madsbad S, Kehlet H. Postoperative morbidity and mortality in type-2 diabetics after fast-track primary total hip and knee arthroplasty. Anesthesia and analgesia. 2015;120(1):230-8.

40. Kandala N-B, Connock M, Pulikottil-Jacob R, Sutcliffe P, Crowther MJ, Grove A, et al. Setting benchmark revision rates for total hip replacement: analysis of registry evidence. BMJ : British Medical Journal. 2015;350.

41. Luchinskaya DS, P. Stoye, G. UK health and social care spending. 2017.

42. NHS_England. Bed Availability and Occupancy. 2018 [cited 05/04/2018]; The KH03 is a quarterly collection from all NHS organisations that operate beds, open overnight or day only. It collects the total number of available bed days and the total number of occupied bed days by consultant main specialty. Data for this collection is available back to 2000-01.]. Available from: https://www.england.nhs.uk/statistics/statistical-work-areas/bed-availability-and-occupancy/

43. Culliford D, Maskell J, Judge A, Cooper C, Prieto-Alhambra D, Arden NK. Future projections of total hip and knee arthroplasty in the UK: results from the UK Clinical Practice Research Datalink. Osteoarthritis and Cartilage. 2015;23(4):594-600.



For peer review only - http://bmjopen.bmj.com/site/about/guidelines.xhtml

44. Briggs T. A national review of adult elective orthopaedic services in England.: British orthopaedic association; 2015.

For peer review only



For peer review only - http://bmjopen.bmj.com/site/about/guidelines.xhtml

## FIGURE LEGENDS

**Fig. 1. Flow diagram showing selection of patients for inclusion in this study (blue shows inclusion and orange shows exclusion).**

**Fig. 2. Effect of the enhanced recovery after surgery (ERAS) programme implemented April 2009 to March 2011 on trends in outcomes following primary hip replacement in England, UK, 2008 – 2016, by month.** (A) Length of hospital stay, (B) change in self-reported pain and function, measured using the Oxford hip score (OHS) at baseline and 6 months after the surgery, (C) any complication in the 6 months after surgery, and (D) hip revision in the 5 years after surgery.

**Fig. 3. Trends in length of hospital stay following primary hip replacement according to patient age categories in England, UK, 2008 – 2016, by month.**
Enhanced recovery after surgery programme implemented in England from April 2009 to March 2011, ERAS.

**Fig. 4. Trends in length of hospital stay following primary hip replacement according to whether patients do or do not present with comorbidities, in England, UK, 2008 – 2016, by month.**

Enhanced recovery after surgery programme implemented in England from April 2009 to March 2011, ERAS.

For peer review only

**Table 1.** Temporal trends in patients undergoing planned primary hip replacement from April 2008 to December 2016. Parsimonious models with Newey-West standard errors.

| **Parameter** | **Coefficient** | **Lower 95% CI** | **Upper 95% CI** | ***P*-value** |
|---|---|---|---|---|
| *Length of stay in hospital* | | | | |
| Intercept | 5.674 | 5.655 | 5.693 | <0.001 |
| Monthly trend | -0.020 | -0.023 | -0.017 | <0.001 |
| Level change $ERAS_0$ | 0.176 | 0.120 | 0.232 | <0.001 |
| Trend change after $ERAS_0$ | -0.013 | -0.017 | -0.009 | <0.001 |
| Level change $ERAS_{end}$ | -0.102 | -0.203 | -0.001 | 0.049 |
| Trend change after $ERAS_{end}$ | 0.019 | 0.015 | 0.022 | <0.001 |
| *Change in Oxford hip score (score at 6 months – score at baseline)* | | | | |
| Intercept | 17.063 | 16.896 | 17.230 | <0.001 |
| Monthly trend | 0.158 | 0.130 | 0.186 | <0.001 |
| Level change $ERAS_0$ | 0.772 | 0.538 | 1.006 | <0.001 |
| Trend change after $ERAS_0$ | -0.131 | -0.161 | -0.101 | <0.001 |
| Level change $ERAS_{end}$ | 0.564 | 0.208 | 0.920 | 0.002 |
| Trend change after $ERAS_{end}$ | -0.013 | -0.025 | -0.001 | 0.039 |
| *Complication by 6 months after surgery* | | | | |
| Intercept | 4.044 | 3.465 | 4.624 | <0.001 |
| Monthly trend | -0.078 | -0.096 | -0.061 | <0.001 |
| Level change $ERAS_0$ | – | – | – | – |
| Trend change after $ERAS_0$ | – | – | – | – |
| Level change $ERAS_{end}$ | – | – | – | – |
| Trend change after $ERAS_{end}$ | 0.078 | 0.056 | 0.100 | <0.001 |



For peer review only

For peer review only - http://bmjopen.bmj.com/site/about/guidelines.xhtml

| | | | | |
|---|---|---|---|---|
| *Revision rates by 5 years after surgery* | | | | |
| Intercept | 7.901 | 7.653 | 8.149 | <0.001 |
| Monthly trend | -0.098 | -0.108 | -0.087 | <0.001 |
| Level change $ERAS_0$ | — | — | — | — |
| Trend change after $ERAS_0$ | — | — | — | — |
| Level change $ERAS_{end}$ | — | — | — | — |
| Trend change after $ERAS_{end}$ | 0.091 | 0.052 | 0.129 | <0.001 |

Confidence intervals, CI; Enhanced Recovery After Surgery, ERAS; start point of ERAS intervention in April 2009, $ERAS_0$; end point of ERAS intervention in March 2011, $ERAS_{end}$; —, $P \geq 0.05$.

For peer review only

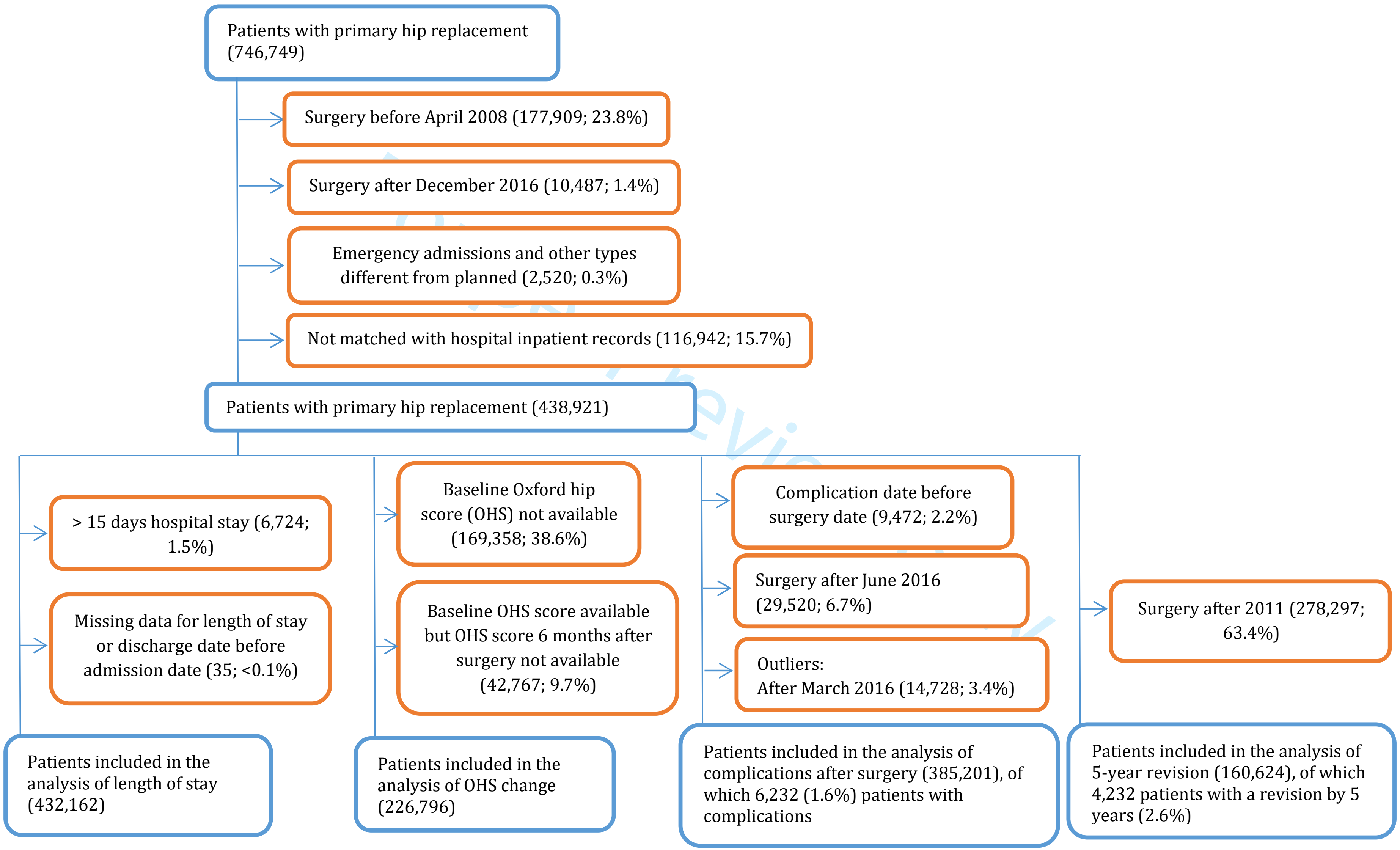
Patients with primary hip replacement (746,749)
Surgery before April 2008 (177,909; 23.8%)
Surgery after December 2016 (10,487; 1.4%)
Emergency admissions and other types different from planned (2,520; 0.3%)
Not matched with hospital inpatient records (116,942; 15.7%)
Patients with primary hip replacement (438,921)
> 15 days hospital stay (6,724; 1.5%)
Missing data for length of stay or discharge date before admission date (35; <0.1%)
Baseline Oxford hip score (OHS) not available (169,358; 38.6%)
Baseline OHS score available but OHS score 6 months after surgery not available (42,767; 9.7%)
Complication date before surgery date (9,472; 2.2%)
Surgery after June 2016 (29,520; 6.7%)
Outliers:
After March 2016 (14,728; 3.4%)
Surgery after 2011 (278,297; 63.4%)
Patients included in the analysis of length of stay (432,162)
Patients included in the analysis of OHS change (226,796)
Patients included in the analysis of complications after surgery (385,201), of which 6,232 (1.6%) patients with complications
Patients included in the analysis of 5-year revision (160,624), of which 4,232 patients with a revision by 5 years (2.6%)

For peer review only

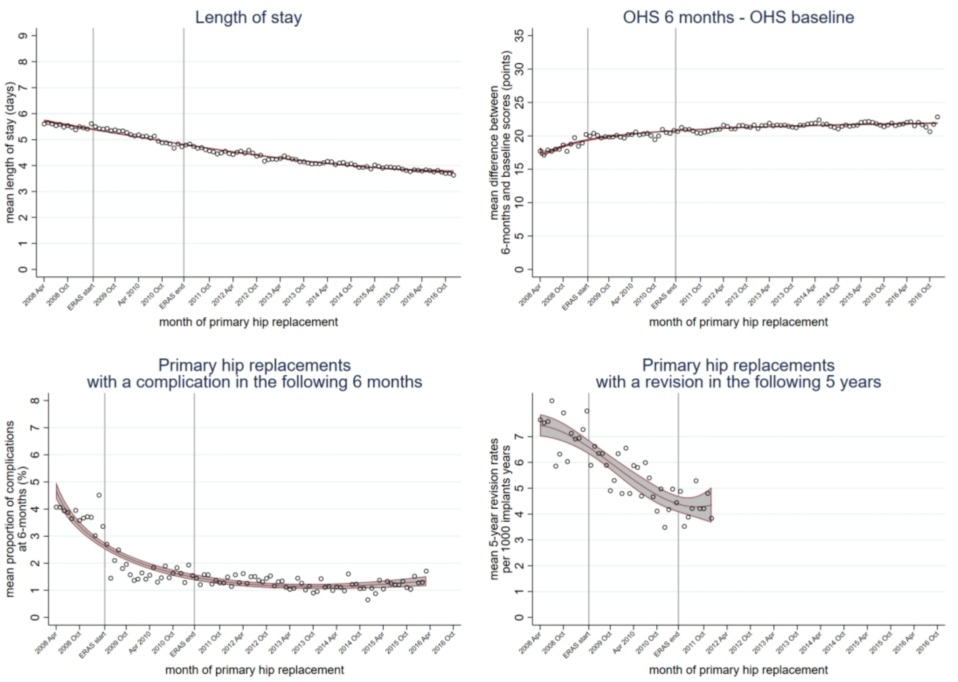


Fig. 2. Effect of the enhanced recovery after surgery (ERAS) programme implemented April 2009 to March 2011 on trends in outcomes following primary hip replacement in England, UK, 2008 – 2016, by month. / (A) Length of hospital stay, (B) change in self-reported pain and function, measured using the Oxford hip score (OHS) at baseline and 6 months after the surgery, (C) any complication in the 6 months after surgery, and (D) hip revision in the 5 years after surgery.

705x513mm (72 x 72 DPI)

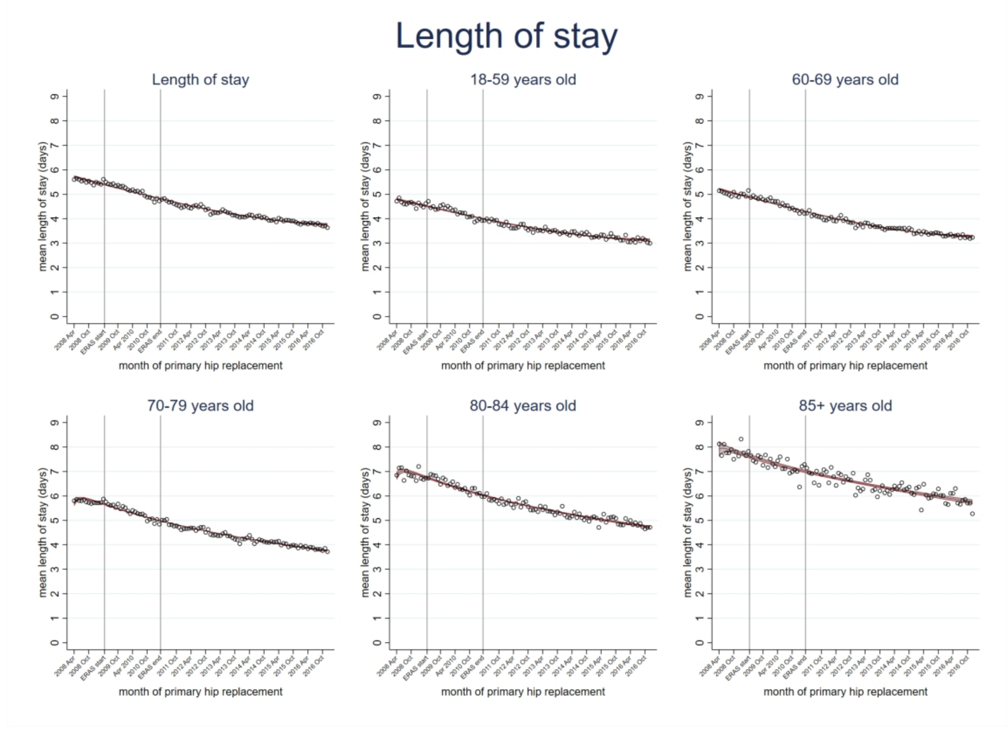


Fig. 3. Trends in length of hospital stay following primary hip replacement according to patient age categories in England, UK, 2008 – 2016, by month. /
Enhanced recovery after surgery programme implemented in England from April 2009 to March 2011, ERAS.

705x513mm (72 x 72 DPI)

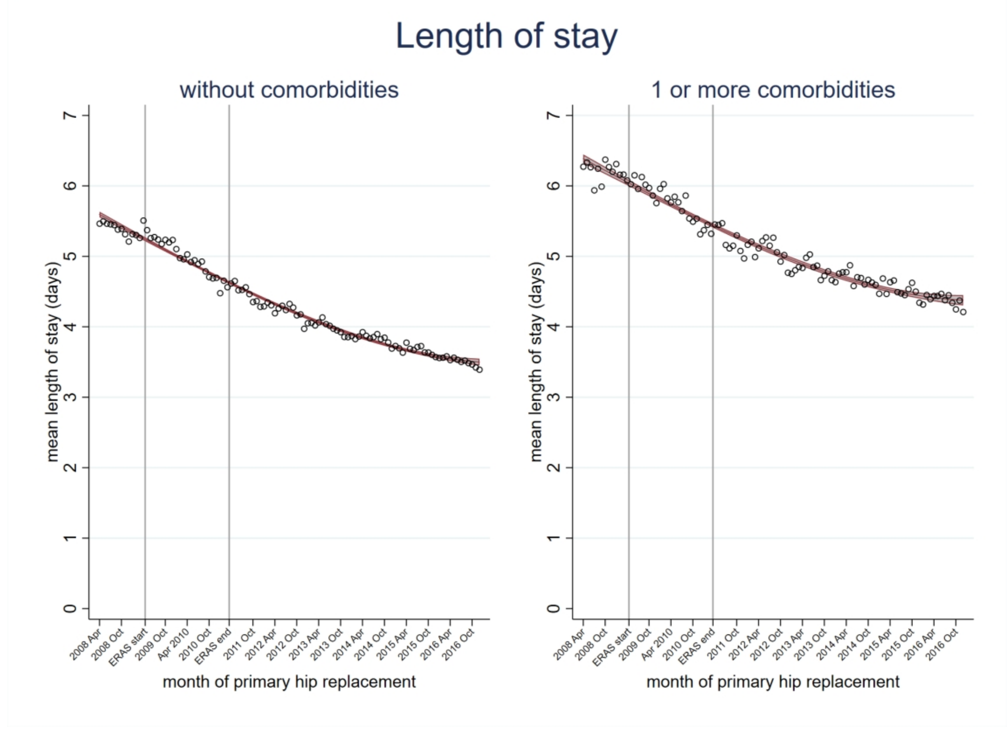


Fig. 4. Trends in length of hospital stay following primary hip replacement according to whether patients do or do not present with comorbidities, in England, UK, 2008 – 2016, by month. /
Enhanced recovery after surgery programme implemented in England from April 2009 to March 2011, ERAS.

705x513mm (72 x 72 DPI)

**MANUSCRIPT TITLE:** Assessment of a National Enhanced Recovery Programme on Patient Outcomes of Primary Hip Replacement: an Interrupted Time Series Analysis from "The National Joint Registry of England and Wales".

## SUPPLEMENTARY MATERIAL

### Supplementary Text S1. Patient and public involvement

During study development, we identified a person who cares for a wife with extensive arthritis and has himself recently undergone hip replacement. He considered this a worthwhile and important project, but cautioned that enhanced recovery could be perceived as a way for hospitals to discharge patients early to save money.

The study was developed in collaboration with the University of Bristol Musculoskeletal Research Unit's Patient Experience Partnership in Research (PEP-R) group, comprising patient representatives with experience of both chronic musculoskeletal conditions and joint replacement. They felt that discharge within four days should not become an NHS target, but that discharge time should instead be tailored to individual needs. They identified specific elements of enhanced recovery that they felt could have had a big impact on their recovery.

The James Lind Alliance has identified the need for involving patients in identifying outcomes that matter to them (patient-identified outcomes). We conducted a patient forum with the PEP-R group to identify outcomes from those available in the routine datasets available for this study. The top outcomes were: 1) pain and function, 2) complications (particularly hospital-acquired infection), 3) length of stay (dependent on the level of support at home), 4) revision surgery, and 5) mortality (rated low importance).



For peer review only - http://bmjopen.bmj.com/site/about/guidelines.xhtml

We met with the PEP-R group to report our findings of the effect of the ERAS programme in primary hip replacement on patient outcomes. The group wanted to see graphs of trends in pain, function, and other outcomes, stratified by age group and co-morbidity. They wanted the graphs to be clear, particularly the scale used when outcomes were rare as a restricted scale could be visually misleading. They also wanted the graphs to show the overall trend rather than trend before, during, and after the implementation of ERAS. They found the study finding that reduced length of stay did not adversely affect patient outcomes to be positive.



For peer review only - http://bmjopen.bmj.com/site/about/guidelines.xhtml

**Supplementary Figure S1. Trends in cost per bed day following primary hip replacement in England, UK, 2008 – 2016, by month.**

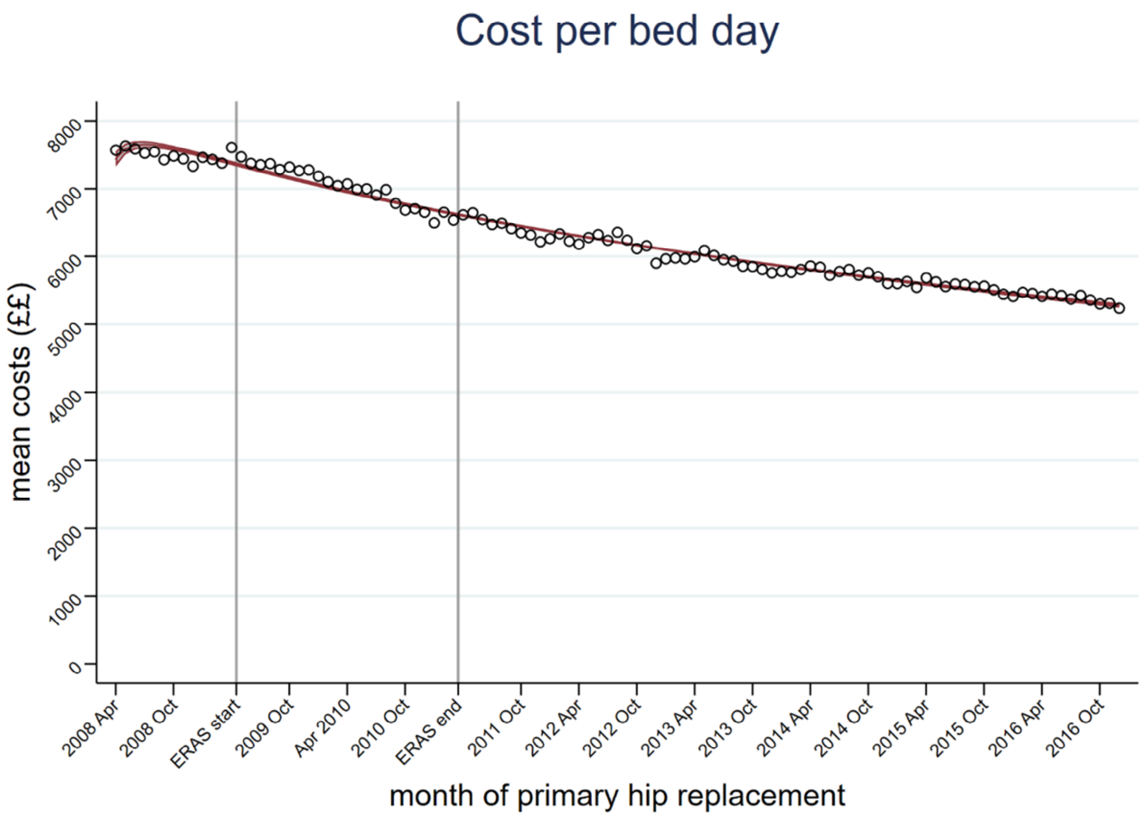


Enhanced recovery after surgery programme implemented in England from April 2009 to March 2011, ERAS.



For peer review only - http://bmjopen.bmj.com/site/about/guidelines.xhtml

**Supplementary Figure S2. Trends in change in Oxford hip score following primary hip replacement according to patient age categories in England, UK, 2008 – 2016, by month.**

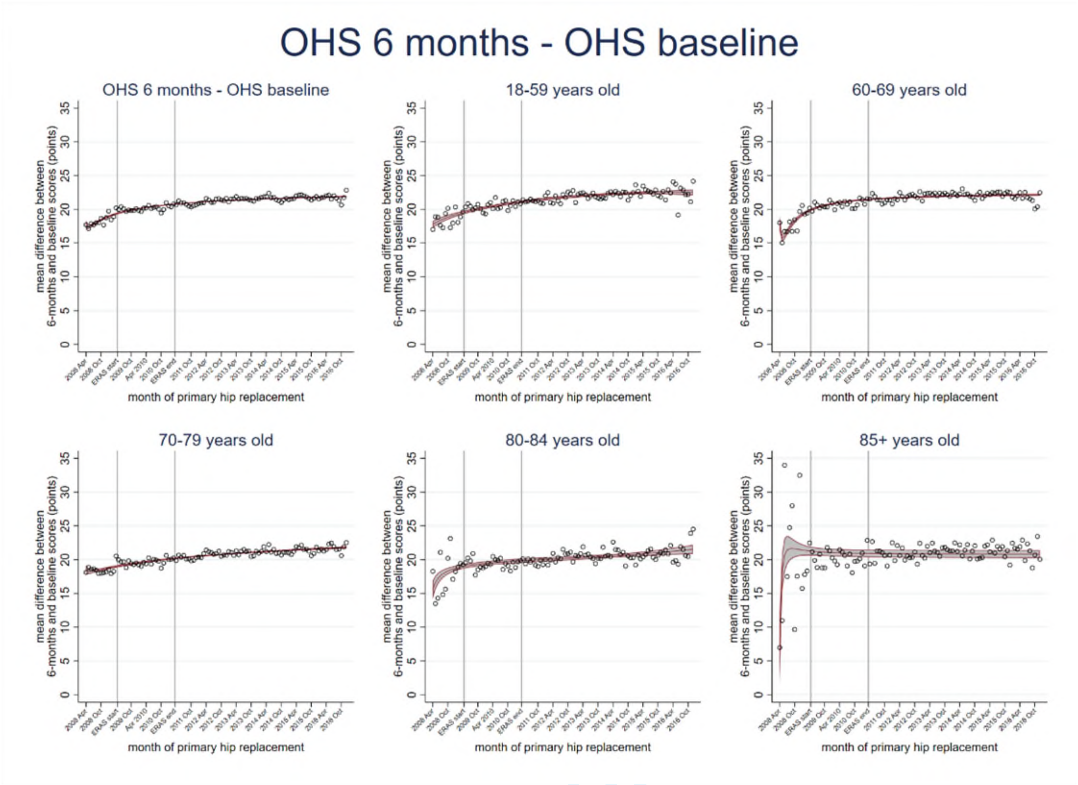


Oxford hip score, OHS; enhanced recovery after surgery programme implemented in England from April 2009 to March 2011, ERAS.



For peer review only - http://bmjopen.bmj.com/site/about/guidelines.xhtml

**Supplementary Figure S3. Trends in change in Oxford hip score following primary hip replacement according to whether patients do or do not present with comorbidities, in England, UK, 2008 – 2016, by month.**

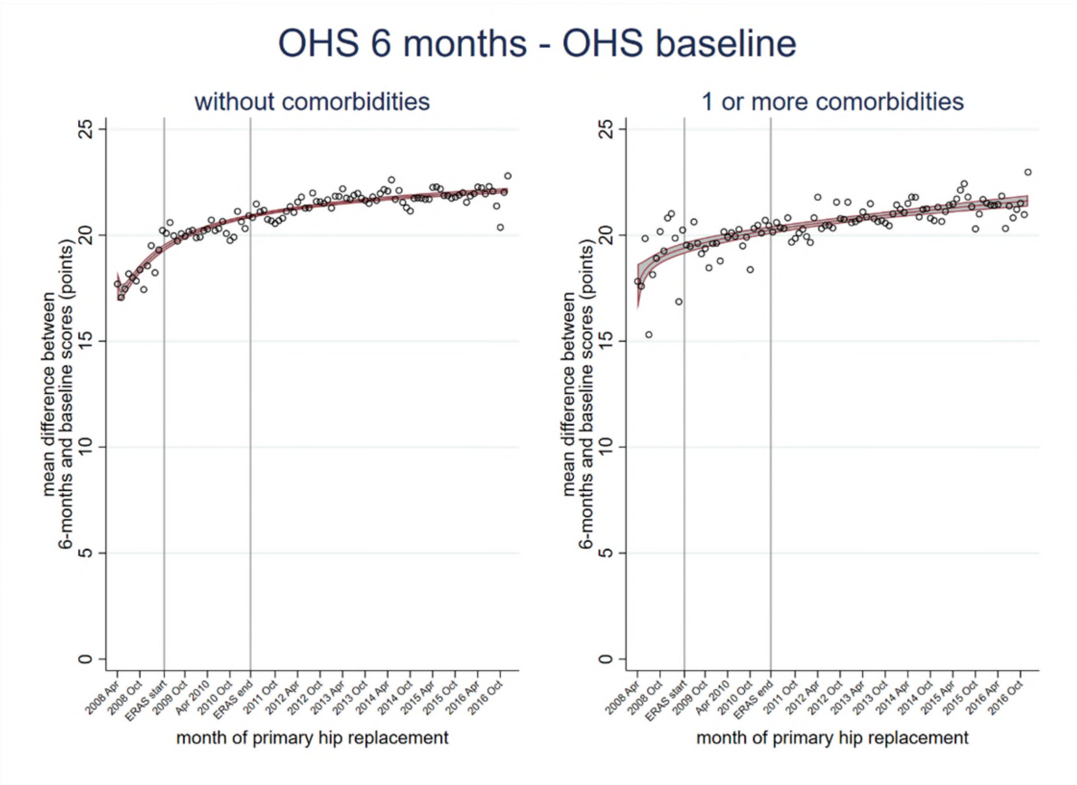


Oxford hip score, OHS; enhanced recovery after surgery programme implemented in England from April 2009 to March 2011, ERAS.



For peer review only - http://bmjopen.bmj.com/site/about/guidelines.xhtml

**Supplementary Table S1.** Temporal trends in patients underwent planned primary hip replacement from April 2008 to December 2016. Full models with Newey-West standard errors.

| **Parameter** | **Coefficient** | **Lower 95% CI** | **Upper 95% CI** | ***P*-value** |
|---|---|---|---|---|
| *Length of stay in hospital* | | | | |
| Intercept | 5.674 | 5.655 | 5.693 | <0.001 |
| Monthly trend | -0.020 | -0.023 | -0.017 | <0.001 |
| Level change $ERAS_0$ | 0.176 | 0.120 | 0.232 | <0.001 |
| Trend change after $ERAS_0$ | -0.013 | -0.017 | -0.009 | <0.001 |
| Level change $ERAS_{end}$ | -0.102 | -0.203 | -0.001 | 0.049 |
| Trend change after $ERAS_{end}$ | 0.019 | 0.015 | 0.022 | <0.001 |
| *Change in Oxford hip score (score at 6 months – score at baseline)* | | | | |
| Intercept | 17.063 | 16.896 | 17.230 | <0.001 |
| Monthly trend | 0.158 | 0.130 | 0.186 | <0.001 |
| Level change $ERAS_0$ | 0.772 | 0.538 | 1.006 | <0.001 |
| Trend change after $ERAS_0$ | -0.131 | -0.161 | -0.101 | <0.001 |
| Level change $ERAS_{end}$ | 0.564 | 0.208 | 0.920 | 0.002 |
| Trend change after $ERAS_{end}$ | -0.013 | -0.025 | -0.001 | 0.039 |
| *Complication rates by 6 months after surgery* | | | | |
| Intercept | 4.254 | 4.161 | 4.347 | <0.001 |
| Monthly trend | -0.072 | -0.088 | -0.056 | <0.001 |
| Level change $ERAS_0$ | -0.729 | -1.531 | 0.072 | 0.074 |
| Trend change after $ERAS_0$ | 0.011 | -0.037 | 0.060 | 0.648 |

For peer review only



For peer review only - http://bmjopen.bmj.com/site/about/guidelines.xhtml

| | | | | |
|---|---|---|---|---|
| Level change $ERAS_{end}$ | 0.179 | -0.372 | 0.730 | 0.521 |
| Trend change after $ERAS_{end}$ | 0.057 | 0.006 | 0.107 | 0.029 |
| *Revision rates by 5 years after surgery* | | | | |
| Intercept | 7.755 | 7.385 | 8.124 | <0.001 |
| Monthly trend | -0.091 | -0.138 | -0.044 | <0.001 |
| Level change $ERAS_0$ | 0.322 | -0.137 | 0.780 | 0.164 |
| Trend change after $ERAS_0$ | -0.023 | -0.083 | 0.036 | 0.432 |
| Level change $ERAS_{end}$ | 0.111 | -0.233 | 0.455 | 0.518 |
| Trend change after $ERAS_{end}$ | 0.112 | 0.066 | 0.157 | <0.001 |

Confidence intervals, CI; Oxford hip score, OHS; Enhanced Recovery After Surgery, ERAS ; start point of ERAS intervention in April 2009, $ERAS_0$; end point of ERAS intervention in March 2011, $ERAS_{end}$.

For peer review only

**Supplementary Table S2.** Temporal trends in patients underwent planned primary hip replacement according to length of stay by age group and comorbidities. April 2008 to December 2016. Parsimonious models with Newey-West standard errors.

| **Parameter** | **Coefficient** | **Lower 95% CI** | **Upper 95% CI** | ***P*-value** |
|---|---|---|---|---|
| *Length of stay in hospital* | | | | |
| *18-59 years old* | | | | |
| Intercept | 4.789 | 4.747 | 4.831 | <0.001 |
| Monthly trend | -0.021 | -0.027 | -0.016 | <0.001 |
| Level change $ERAS_0$ | 0.189 | 0.067 | 0.310 | 0.003 |
| Trend change after $ERAS_0$ | -0.012 | -0.019 | -0.006 | <0.001 |
| Level change $ERAS_{end}$ | – | – | – | – |
| Trend change after $ERAS_{end}$ | 0.021 | 0.014 | 0.027 | <0.001 |
| *60-69 years old* | | | | |
| Intercept | 5.126 | 5.063 | 5.188 | <0.001 |
| Monthly trend | -0.015 | -0.024 | -0.006 | 0.001 |
| Level change $ERAS_0$ | 0.129 | 0.047 | 0.211 | 0.002 |
| Trend change after $ERAS_0$ | -0.017 | -0.025 | -0.009 | <0.001 |
| Level change $ERAS_{end}$ | -0.142 | -0.238 | -0.046 | 0.004 |
| Trend change after $ERAS_{end}$ | 0.018 | 0.015 | 0.021 | <0.001 |
| *70-79 years old* | | | | |
| Intercept | 5.875 | 5.858 | 5.892 | <0.001 |
| Monthly trend | -0.015 | -0.017 | -0.012 | <0.001 |


For peer review only - http://bmjopen.bmj.com/site/about/guidelines.xhtml

| | | | | |
|---|---|---|---|---|
| Level change $ERAS_0$ | 0.189 | 0.133 | 0.244 | <0.001 |
| Trend change after $ERAS_0$ | -0.026 | -0.030 | -0.022 | <0.001 |
| Level change $ERAS_{end}$ | — | — | — | — |
| Trend change after $ERAS_{end}$ | 0.023 | 0.019 | 0.028 | <0.001 |
| *80-84 years old* | | | | |
| Intercept | 6.896 | 6.848 | 6.943 | <0.001 |
| Monthly trend | — | — | — | — |
| Level change $ERAS_0$ | — | — | — | — |
| Trend change after $ERAS_0$ | -0.033 | -0.036 | -0.030 | <0.001 |
| Level change $ERAS_{end}$ | -0.179 | -0.246 | -0.112 | <0.001 |
| Trend change after $ERAS_{end}$ | 0.016 | 0.012 | 0.019 | <0.001 |
| *≥85 years old* | | | | |
| Intercept | 7.855 | 7.801 | 7.908 | <0.001 |
| Monthly trend | — | — | — | — |
| Level change $ERAS_0$ | -0.126 | -0.201 | -0.051 | 0.001 |
| Trend change after $ERAS_0$ | -0.032 | -0.036 | -0.027 | <0.001 |
| Level change $ERAS_{end}$ | — | — | — | — |
| Trend change after $ERAS_{end}$ | 0.014 | 0.008 | 0.020 | <0.001 |
| *Without comorbidities* | | | | |
| Intercept | 5.554 | 5.532 | 5.576 | <0.001 |
| Monthly trend | -0.024 | -0.027 | -0.021 | <0.001 |
| Level change $ERAS_0$ | 0.239 | 0.168 | 0.309 | <0.001 |
| Trend change after $ERAS_0$ | -0.016 | -0.022 | -0.010 | <0.001 |
| Level change $ERAS_{end}$ | — | — | — | — |



For peer review only - http://bmjopen.bmj.com/site/about/guidelines.xhtml

| | | | | |
|---|---|---|---|---|
| Trend change after $ERAS_{end}$ | 0.023 | 0.018 | 0.029 | <0.001 |
| *1+ comorbidities* | | | | |
| Intercept | 6.218 | 6.186 | 6.250 | <0.001 |
| Monthly trend | — | — | — | — |
| Level change $ERAS_0$ | — | — | — | — |
| Trend change after $ERAS_0$ | -0.032 | -0.036 | -0.029 | <0.001 |
| Level change $ERAS_{end}$ | -0.129 | -0.240 | -0.018 | 0.024 |
| Trend change after $ERAS_{end}$ | 0.018 | 0.014 | 0.021 | <0.001 |

Confidence intervals, CI; Enhanced Recovery After Surgery, ERAS ; start point of the ERAS intervention in April 2009, $ERAS_0$; end point of the ERAS intervention in March 2011, $ERAS_{end}$; —, $P \geq 0.05$.

For peer review only



For peer review only - http://bmjopen.bmj.com/site/about/guidelines.xhtml

**Supplementary Table S3.** Temporal trends in patients underwent planned primary hip replacement according to length of stay by age group and comorbidities. April 2008 to December 2016. Full models with Newey-West standard errors.

| **Parameter** | **Coefficient** | **Lower 95% CI** | **Upper 95% CI** | ***P*-value** |
|---|---|---|---|---|
| *Length of stay in hospital* | | | | |
| *18-59 years old* | | | | |
| Intercept | 4.789 | 4.747 | 4.831 | <0.001 |
| Monthly trend | -0.021 | -0.027 | -0.016 | <0.001 |
| Level change $ERAS_0$ | 0.152 | 0.049 | 0.255 | 0.004 |
| Trend change after $ERAS_0$ | -0.008 | -0.015 | -0.001 | 0.028 |
| Level change $ERAS_{end}$ | -0.095 | -0.225 | 0.035 | 0.149 |
| Trend change after $ERAS_{end}$ | 0.017 | 0.011 | 0.022 | <0.001 |
| *60-69 years old* | | | | |
| Intercept | 5.126 | 5.063 | 5.188 | <0.001 |
| Monthly trend | -0.015 | -0.024 | -0.006 | 0.001 |
| Level change $ERAS_0$ | 0.129 | 0.047 | 0.211 | 0.002 |
| Trend change after $ERAS_0$ | -0.017 | -0.025 | -0.009 | <0.001 |
| Level change $ERAS_{end}$ | -0.142 | -0.238 | -0.046 | 0.004 |
| Trend change after $ERAS_{end}$ | 0.018 | 0.015 | 0.021 | <0.001 |
| *70-79 years old* | | | | |
| Intercept | 5.875 | 5.858 | 5.892 | <0.001 |
| Monthly trend | -0.015 | -0.017 | -0.012 | <0.001 |
| Level change $ERAS_0$ | 0.159 | 0.125 | 0.193 | <0.001 |



For peer review only - http://bmjopen.bmj.com/site/about/guidelines.xhtml

| | | | | |
|---|---|---|---|---|
| Trend change after $ERAS_0$ | -0.022 | -0.026 | -0.019 | <0.001 |
| Level change $ERAS_{end}$ | -0.076 | -0.161 | 0.008 | 0.077 |
| Trend change after $ERAS_{end}$ | 0.020 | 0.018 | 0.023 | <0.001 |
| *80-84 years old* | | | | |
| Intercept | 7.016 | 6.931 | 7.102 | <0.001 |
| Monthly trend | -0.017 | -0.029 | -0.005 | 0.005 |
| Level change $ERAS_0$ | 0.079 | -0.019 | 0.177 | 0.113 |
| Trend change after $ERAS_0$ | -0.016 | -0.028 | -0.003 | 0.015 |
| Level change $ERAS_{end}$ | -0.181 | -0.250 | -0.112 | <0.001 |
| Trend change after $ERAS_{end}$ | 0.015 | 0.011 | 0.020 | <0.001 |
| *≥85 years old* | | | | |
| Intercept | 7.902 | 7.743 | 8.062 | <0.001 |
| Monthly trend | -0.007 | -0.030 | 0.016 | 0.562 |
| Level change $ERAS_0$ | -0.070 | -0.240 | 0.100 | 0.415 |
| Trend change after $ERAS_0$ | -0.027 | -0.050 | -0.004 | 0.020 |
| Level change $ERAS_{end}$ | 0.056 | -0.083 | 0.195 | 0.425 |
| Trend change after $ERAS_{end}$ | 0.016 | 0.010 | 0.022 | <0.001 |
| *Without comorbidities* | | | | |
| Intercept | 5.554 | 5.532 | 5.576 | <0.001 |
| Monthly trend | -0.024 | -0.027 | -0.021 | <0.001 |
| Level change $ERAS_0$ | 0.201 | 0.155 | 0.247 | <0.001 |
| Trend change after $ERAS_0$ | -0.012 | -0.017 | -0.007 | <0.001 |
| Level change $ERAS_{end}$ | -0.096 | -0.202 | 0.009 | 0.074 |
| Trend change after $ERAS_{end}$ | 0.020 | 0.017 | 0.022 | <0.001 |

For peer review only

*1+ comorbidities*

| | | | | |
|---|---|---|---|---|
| Intercept | 6.220 | 6.134 | 6.305 | <0.001 |
| Monthly trend | -0.001 | -0.011 | 0.010 | 0.902 |
| Level change $ERAS_0$ | 0.012 | -0.088 | 0.112 | 0.813 |
| Trend change after $ERAS_0$ | -0.032 | -0.042 | -0.022 | <0.001 |
| Level change $ERAS_{end}$ | -0.126 | -0.245 | -0.007 | 0.038 |
| Trend change after $ERAS_{end}$ | 0.018 | 0.013 | 0.023 | <0.001 |

Confidence intervals, CI; Enhanced Recovery After Surgery, ERAS ; start point of ERAS intervention in April 2009, $ERAS_0$; end point of ERAS intervention in March 2011, $ERAS_{end}$.



For peer review only - http://bmjopen.bmj.com/site/about/guidelines.xhtml

**Supplementary Table S4.** Distribution of primary hip replacements and percentage of missing values per potential modifier of OHS. England (April 2008 to December 2016)

| | **n** | **%** | **% missing values** | | |
|---|---|---|---|---|---|
| | | | **OHS at baseline** | **OHS at 6 months** | **OHS change** |
| *Total* | 438,921 | 100 | 38.6 | 47.9 | 48.3 |
| *Period* | | | *P*<0.001 | *P*<0.001 | *P*<0.001 |
| Before ERAS (April 2008 - March 2009) | 38,362 | 8.7 | 87.4 | 89.6 | 89.7 |
| During ERAS (April 2009 - March 2011) | 86,416 | 19.7 | 33.2 | 41.6 | 41.9 |
| After ERAS (April 2011 - December 2016) | 314,143 | 71.6 | 34.1 | 44.6 | 45.0 |
| *Age at surgery (years)* | | | *P*<0.001 | *P*<0.001 | *P*<0.001 |
| 18-59 | 78,383 | 17.9 | 36.9 | 52.7 | 52.9 |
| 60-69 | 13 1,441 | 30.0 | 36.1 | 44.4 | 44.7 |
| 70-79 | 157.241 | 35.8 | 39.0 | 46.2 | 46.7 |
| 80-84 | 47,919 | 10.9 | 42.5 | 50.8 | 51.3 |
| 85 or more | 23,937 | 5.5 | 47.3 | 56.9 | 57.5 |
| *Comorbidities* | | | *P*<0.001 | *P*<0.001 | *P*<0.001 |
| None | 325,750 | 74.2 | 38.3 | 47.1 | 47.5 |
| 1 or more | 113,171 | 25.8 | 39.5 | 50.3 | 50.7 |

Oxford hip score, OHS; Enhanced Recovery After Surgery, ERAS . *P* values are calculated from 2-sided Pearson's statistic. OHS change is the difference between OHS at 6 months and OHS at baseline.



For peer review only - http://bmjopen.bmj.com/site/about/guidelines.xhtml

**Supplementary Table S5.** Distribution of potential modifiers for primary hip replacements with complete and incomplete data for change in OHS (6 month - baseline scores). England (April 2008 to December 2016)

| | Incomplete cases | | Complete cases | | *P* value |
|---|---|---|---|---|---|
| | **n** | **%** | **n** | **%** | |
| *Period* | | | | | *P*<0.001 |
| Before ERAS (April 2008 - March 2009) | 34,405 | 16.2 | 3,957 | 1.7 | |
| During ERAS (April 2009 - March 2011) | 36,242 | 17.1 | 50,174 | 22.1 | |
| After ERAS (April 2011 - December 2016) | 141,478 | 66.7 | 172,665 | 76.1 | |
| *Age at surgery (years)* | | | | | *P*<0.001 |
| 18-59 | 41,497 | 19.6 | 36,886 | 16.3 | |
| 60-69 | 58,812 | 27.7 | 72,629 | 32.0 | |
| 70-79 | 73,480 | 34.6 | 83,761 | 36.9 | |
| 80-84 | 24,584 | 11.6 | 23,335 | 10.3 | |
| 85 or more | 13,752 | 6.5 | 10,185 | 4.5 | |
| *Comorbidities* | | | | | *P*<0.001 |
| None | 154,718 | 72.9 | 171032.0 | 75.4 | |
| 1 or more | 57,407 | 27.1 | 55764.0 | 24.6 | |



For peer review only - http://bmjopen.bmj.com/site/about/guidelines.xhtml

Oxford hip score, OHS; Enhanced Recovery After Surgery, ERAS . *P* values are calculated from 2-sided Pearson's statistic. Incomplete cases defined by a missing value in OHS at baseline or OHS at 6 months.

For peer review only

**Supplementary Table S6.** Temporal trends in patients underwent planned primary hip replacement according to OHS change by age group and comorbidities. April 2008 to December 2016. Parsimonious models with Newey-West standard errors.

| Parameter | Coefficient | Lower 95% CI | Upper 95% CI | *P*-value |
|---|---|---|---|---|
| *Change in Oxford hip score (score at 6 months – score at baseline)* | | | | |
| *18-59 years old* | | | | |
| Intercept | 18.096 | 17.853 | 18.340 | <0.001 |
| Monthly trend | 0.052 | 0.034 | 0.070 | <0.001 |
| Level change $ERAS_0$ | 1.039 | 0.617 | 1.461 | <0.001 |
| Trend change after $ERAS_0$ | – | – | – | – |
| Level change $ERAS_{end}$ | 0.334 | 0.014 | 0.655 | 0.041 |
| Trend change after $ERAS_{end}$ | -0.031 | -0.052 | -0.011 | 0.003 |
| *60-69 years old* | | | | |
| Intercept | 15.185 | 14.473 | 15.897 | <0.001 |
| Monthly trend | 0.395 | 0.320 | 0.471 | <0.001 |
| Level change $ERAS_0$ | | | | |
| Trend change after $ERAS_0$ | -0.344 | -0.433 | -0.254 | <0.001 |
| Level change $ERAS_{end}$ | 0.639 | 0.076 | 1.203 | 0.027 |
| Trend change after $ERAS_{end}$ | -0.048 | -0.074 | -0.021 | 0.001 |
| *70-79 years old* | | | | |
| Intercept | 18.645 | 18.474 | 18.816 | <0.001 |
| Monthly trend | -0.047 | -0.068 | -0.026 | <0.001 |



For peer review only - http://bmjopen.bmj.com/site/about/guidelines.xhtml

| | | | | |
|---|---|---|---|---|
| Level change $ERAS_0$ | 1.269 | 1.109 | 1.429 | <0.001 |
| Trend change after $ERAS_0$ | 0.068 | 0.046 | 0.089 | <0.001 |
| Level change $ERAS_{end}$ | 0.494 | 0.158 | 0.829 | 0.004 |
| Trend change after $ERAS_{end}$ | — | — | — | — |
| *80-84 years old* | | | | |
| Intercept | 15.235 | 14.237 | 16.232 | <0.001 |
| Monthly trend | 0.358 | 0.262 | 0.454 | <0.001 |
| Level change $ERAS_0$ | — | — | — | — |
| Trend change after $ERAS_0$ | -0.370 | -0.480 | -0.260 | <0.001 |
| Level change $ERAS_{end}$ | 0.548 | 0.028 | 1.068 | 0.039 |
| Trend change after $ERAS_{end}$ | 0.034 | 0.007 | 0.061 | 0.016 |
| *≥85 years old* | | | | |
| Intercept | 19.946 | 19.233 | 20.658 | <0.001 |
| Monthly trend | — | — | — | — |
| Level change $ERAS_0$ | — | — | — | — |
| Trend change after $ERAS_0$ | — | — | — | — |
| Level change $ERAS_{end}$ | 1.249 | 0.504 | 1.993 | 0.001 |
| Trend change after $ERAS_{end}$ | — | — | — | — |
| *Without comorbidities* | | | | |
| Intercept | 17.106 | 16.939 | 17.273 | <0.001 |
| Monthly trend | 0.133 | 0.105 | 0.160 | <0.001 |
| Level change $ERAS_0$ | 1.306 | 1.088 | 1.525 | <0.001 |
| Trend change after $ERAS_0$ | -0.118 | -0.147 | -0.090 | <0.001 |
| Level change $ERAS_{end}$ | 0.795 | 0.352 | 1.237 | 0.001 |



For peer review only - http://bmjopen.bmj.com/site/about/guidelines.xhtml

| | | | | |
|---|---|---|---|---|
| Trend change after $ERAS_{end}$ | — | — | — | — |
| *1+ comorbidities* | | | | |
| Intercept | 16.642 | 16.003 | 17.282 | <0.001 |
| Monthly trend | 0.334 | 0.260 | 0.408 | <0.001 |
| Level change $ERAS_0$ | -1.677 | -2.132 | -1.223 | <0.001 |
| Trend change after $ERAS_0$ | -0.279 | -0.357 | -0.202 | <0.001 |
| Level change $ERAS_{end}$ | — | — | — | — |
| Trend change after $ERAS_{end}$ | -0.034 | -0.056 | -0.013 | 0.002 |

Oxford hip score, OHS; confidence intervals, CI; Enhanced Recovery After Surgery, ERAS ; start point of ERAS intervention in April 2009, $ERAS_0$; end point of ERAS intervention in March 2011, $ERAS_{end}$; —, $P \geq 0.05$.

For peer review only



For peer review only - http://bmjopen.bmj.com/site/about/guidelines.xhtml

**Supplementary Table S7.** Temporal trends in patients underwent planned primary hip replacement according to OHS change by age group and comorbidities. April 2008 to December 2016. Full models with Newey-West standard errors.

| Parameter | Coefficient | Lower 95% CI | Upper 95% CI | *P*-value |
|---|---|---|---|---|
| *Change in Oxford hip score (score at 6 months – score at baseline)* | | | | |
| *18-59 years old* | | | | |
| Intercept | 17.781 | 17.368 | 18.193 | <0.001 |
| Monthly trend | 0.097 | 0.040 | 0.154 | 0.001 |
| Level change $ERAS_0$ | 0.867 | 0.439 | 1.295 | <0.001 |
| Trend change after $ERAS_0$ | -0.049 | -0.110 | 0.011 | 0.110 |
| Level change $ERAS_{end}$ | 0.384 | 0.070 | 0.698 | 0.017 |
| Trend change after $ERAS_{end}$ | -0.027 | -0.045 | -0.010 | 0.003 |
| *60-69 years old* | | | | |
| Intercept | 15.451 | 14.644 | 16.257 | <0.001 |
| Monthly trend | 0.342 | 0.235 | 0.450 | <0.001 |
| Level change $ERAS_0$ | 0.578 | -0.102 | 1.259 | 0.095 |
| Trend change after $ERAS_0$ | -0.303 | -0.406 | -0.200 | <0.001 |
| Level change $ERAS_{end}$ | 0.737 | 0.173 | 1.300 | 0.011 |
| Trend change after $ERAS_{end}$ | -0.035 | -0.057 | -0.013 | 0.002 |
| *70-79 years old* | | | | |
| Intercept | 18.645 | 18.473 | 18.817 | <0.001 |
| Monthly trend | -0.047 | -0.068 | -0.026 | <0.001 |
| Level change $ERAS_0$ | 1.264 | 0.977 | 1.552 | <0.001 |



For peer review only - http://bmjopen.bmj.com/site/about/guidelines.xhtml

For peer review only

| | | | | |
|---|---|---|---|---|
| Trend change after $ERAS_0$ | 0.068 | 0.040 | 0.096 | <0.001 |
| Level change $ERAS_{end}$ | 0.490 | 0.106 | 0.874 | 0.013 |
| Trend change after $ERAS_{end}$ | 0.000 | -0.018 | 0.017 | 0.967 |
| *80-84 years old* | | | | |
| Intercept | 15.207 | 14.066 | 16.349 | <0.001 |
| Monthly trend | 0.364 | 0.217 | 0.511 | <0.001 |
| Level change $ERAS_0$ | -0.060 | -0.965 | 0.844 | 0.895 |
| Trend change after $ERAS_0$ | -0.374 | -0.522 | -0.226 | <0.001 |
| Level change $ERAS_{end}$ | 0.537 | 0.015 | 1.060 | 0.044 |
| Trend change after $ERAS_{end}$ | 0.033 | 0.008 | 0.057 | 0.011 |
| *≥85 years old* | | | | |
| Intercept | 16.187 | 9.205 | 23.168 | <0.001 |
| Monthly trend | 0.487 | -0.395 | 1.368 | 0.276 |
| Level change $ERAS_0$ | -1.708 | -5.891 | 2.474 | 0.420 |
| Trend change after $ERAS_0$ | -0.503 | -1.386 | 0.380 | 0.261 |
| Level change $ERAS_{end}$ | 1.014 | 0.495 | 1.532 | <0.001 |
| Trend change after $ERAS_{end}$ | 0.024 | -0.005 | 0.053 | 0.099 |
| *Without comorbidities* | | | | |
| Intercept | 17.106 | 16.938 | 17.274 | <0.001 |
| Monthly trend | 0.133 | 0.105 | 0.160 | <0.001 |
| Level change $ERAS_0$ | 1.187 | 0.967 | 1.406 | <0.001 |
| Trend change after $ERAS_0$ | -0.109 | -0.137 | -0.080 | <0.001 |
| Level change $ERAS_{end}$ | 0.698 | 0.313 | 1.083 | 0.001 |
| Trend change after $ERAS_{end}$ | -0.010 | -0.021 | 0.001 | 0.083 |
| *1+ comorbidities* | | | | |

For peer review only

| | | | | |
|---|---|---|---|---|
| Intercept | 16.642 | 16.000 | 17.285 | <0.001 |
| Monthly trend | 0.334 | 0.260 | 0.408 | <0.001 |
| Level change ERAS$_0$ | -1.640 | -2.136 | -1.144 | <0.001 |
| Trend change after ERAS$_0$ | -0.284 | -0.362 | -0.206 | <0.001 |
| Level change ERAS$_{end}$ | 0.097 | -0.279 | 0.472 | 0.610 |
| Trend change after ERAS$_{end}$ | -0.030 | -0.054 | -0.006 | 0.013 |

Oxford hip score, OHS; confidence intervals, CI; Enhanced Recovery After Surgery, ERAS; start point of ERAS intervention in April 2009, ERAS$_0$; end point of ERAS intervention in March 2011, ERAS$_{end}$.

For peer review only

**MANUSCRIPT TITLE:**

Impact of a National Enhanced Recovery Programme on Patient Outcomes of Primary Hip Replacement: an Interrupted Time Series Analysis from England.

## APPENDIXES

**Appendix 1.** Cost methods.

Objective

We aimed to estimate the trend in National Health Service (NHS) expenditure over time, reflecting the change in length of stay (LOS) observed.

Grouper and reference cost methods

Using Hospital Episode Statistics (HES) data for the same group of patients as for LOS (i.e. excluding those with length of stay above 15 days), we generated healthcare resource use group (HRG) classifications for the index episode for each patient using the 2015/16 NHS reference costs grouper [1], which were subsequently used to estimate inpatient costs per patient using NHS reference costs from 2015/16 [2].

86% of the index episodes in the data had a LOS below the trim point for the relevant HRG. For example, the most common HRG in the data was hip replacement without complications (HN12F: "Very Major Hip Procedures for Non-Trauma with CC Score 0-1" [2]), constituting 63% of the index episodes in the data. This HRG has a trim point of 6 days, meaning that all episodes with a duration less than 6 days are assigned the same unit cost. A reduction in LOS within the trim point is therefore not reflected in the cost of the episode, despite there being a true reduction in NHS costs. In order to estimate the mean change in NHS expenditure we therefore estimated an adjusted average bed day cost.



For peer review only - http://bmjopen.bmj.com/site/about/guidelines.xhtml

Estimating the adjusted average bed day cost

For each HRG we estimated the average cost per bed day (defined as any part of a day spent in hospital) by dividing the total cost of the index episodes for that HRG by the total number of bed days for that HRG. This generated a single average bed day cost per HRG.

For each patient we estimated the adjusted episode cost by multiplying their length of stay (bed days) by the average bed day cost for the HRG that they had been assigned by the NHS reference costs grouper [1]. Therefore, instead of assigning the same unit cost to all patients with the same HRG who had a LOS below the trim point, the adjusted cost differed according to a patient's LOS, even if that LOS was below the trim point for the HRG. Using this method we were able to estimate the average difference in true NHS expenditure as a result of the reduction in length of stay over time even when the LOS was below the trim point.

The 2015/16 grouper and reference costs [1,2] were used to estimate costs for all patients in all years, as there are differences in the methodologies used for HRG classification in different cost years [3]. This prevents a like-for-like comparison between years if different groupers and/or costs are used.

Costs were estimated for a total of 432,143 patients.

References for Appendix 1

1. HRG4+ 2015/16 Reference Costs Grouper. Copyright © 2015 Health and Social Care Information Centre. Grouper version: RC 15/16. Implementation version: 1516.RC.8
2. Department of Health. NHS reference costs 2015 to 2016. https://www.gov.uk/government/publications/nhs-reference-costs-2015-to-2016
3. Reference Costs 2015-16. Department of Health. https://assets.publishing.service.gov.uk/government/uploads/system/uploads/attachment_data/file/577083/Reference_Costs_2015-16.pdf.



For peer review only - http://bmjopen.bmj.com/site/about/guidelines.xhtml

**Appendix 2.** Operative procedure codes (OPCS 4.8) that we used to identify hip revision in the Hospital Episode Statistics (HES) registry.

Procedure type 1: : W39.4, “Attention to total prosthetic replacement of hip joint (Not Elsewhere Classified, NEC)”; W39.6, “Closed reduction of dislocated total prosthetic replacement of hip joint”; W78.2, “Release of contracture of hip joint”; W95.4, “Attention to hybrid prosthetic replacement of hip joint using cement NEC”; X09.1, “Hindquarter amputation”; X09.2, “Disarticulation of hip”; X09.3, “Amputation of leg above knee”.

Procedure type 2: W07.2, “Excision of periarticular ectopic bone”; W18.1, “Fenestration of cortex of bone”; W18.2, “Saucerisation of bone”; W18.3, “Sequestrectomy of bone”; W18.4, “Decompression of fourage of bone”; W18.5, “Insertion of drainage system into bone”; W33.6, “Debridement of bone NEC”; W33.7, “Lavage of bone”; W33.8, “Other specified other open operations on bone”; W33.9, “Unspecified other open operations on bone”; W45.4, “Attention to total prosthetic replacement of joint NEC”; W54.4, “Attention to prosthetic interposition arthroplasty of joint NEC”; W60.3, “Conversion to arthrodesis and extra-articular bone graft NEC”; W60.8, “Other specified fusion of other joint and extra-articular bone graft”; W60.9, “Unspecified fusion of other joint and extra-articular bone graft”; W61.3, “Conversion to arthrodesis and articular bone graft NEC”; W61.8, “Other specified fusion of other joint and other articular bone graft”; W61.9, “Unspecified fusion of other joint and other articular bone graft”; W63.1, “Revision of arthrodesis and internal fixation NEC”; W63.2, “Revision of arthrodesis and external fixation NEC”; W63.8, “Other specified revisional fusion of other joint”; W63.9, “Unspecified revisional fusion of other joint”; W64.0, “Conversion from previous arthrodesis NEC”; W64.1, “Conversion to arthrodesis and internal fixation NEC”; W64.2, “Conversion to arthrodesis and external fixation NEC”; W64.8, “Other specified conversion to fusion of other joint”; W64.9, “Unspecified conversion to fusion of other joint”; W69.1, “Total synovectomy”; W69.2,



For peer review only - http://bmjopen.bmj.com/site/about/guidelines.xhtml

"Subtotal synovectomy"; W69.3, "Partial synovectomy"; W69.4, "Open biopsy of synovial membrane of joint"; W69.5, "Open division of synovial plica"; W69.8, "Other specified open operations on synovial membrane of joint"; W69.9, "Unspecified open operations on synovial membrane of joint"; W71.2, "Open excision of intra-articular osteophyte"; W71.3, "Forage of joint"; W71.8, "Other specified other open operations on intra-articular structure"; W71.9, "Unspecified other open operations on intra-articular structure"; W77.1, "Repair of capsule of joint for stabilisation of joint NEC"; W77.2, "Transposition of muscle for stabilisation of joint"; W77.3, "Blocking operations on joint using prosthesis for stabilisation of joint"; W77.4, "Blocking operations on joint using bone for stabilisation of joint"; W77.5, "Periarticular osteotomy for stabilisation of joint"; W77.8, "Other specified stabilising operations on joint"; W77.9, "Unspecified stabilising operations on joint"; W78.4, "Limited release of contracture of capsule of joint"; W78.8, "Other specified release of contracture of joint"; W78.9, "Unspecified release of contracture of joint"; W80.1, "Open debridement and irrigation of joint"; W80.2, "Open debridement of joint NEC"; W80.3, "Open irrigation of joint NEC"; W80.8, "Other specified debridement and irrigation of joint"; W80.9, "Unspecified debridement and irrigation of joint"; W81.1, "Excision of lesion of joint NEC"; W81.2, "Open removal of loose body from joint"; W81.3, "Drainage of joint"; W81.4, "Incision of joint NEC"; W81.5, "Exploration of joint NEC"; W81.6, "Capsulorrhaphy of joint"; W81.7, "Insertion of therapeutic spacer into joint"; W81.8, "Other specified other open operations on joint"; W81.9, "Unspecified other open operations on joint"; W84.3, "Endoscopic division of synovial plica"; W84.4, "Endoscopic decompression of joint"; W84.6, "Endoscopic excision of synovial plica"; W84.8, "Other specified therapeutic endoscopic operations on other joint structure"; W84.9, "Unspecified therapeutic endoscopic operations on other joint structure"; W86.1, "Endoscopic removal of loose body from joint NEC"; W86.8, "Other specified therapeutic endoscopic operations on cavity of other joint";



For peer review only - http://bmjopen.bmj.com/site/about/guidelines.xhtml

W86.9, “Unspecified therapeutic endoscopic operations on cavity of other joint”; W88.1, “Diagnostic endoscopic examination of joint and biopsy of lesion of joint NEC”; W88.8, “Other specified diagnostic endoscopic examination of other joint”; W88.9, “Unspecified diagnostic endoscopic examination of other joint”; W90.1, “Aspiration of joint”; W90.2, “Arthrography”; W90.3, “Injection of therapeutic substance into joint”; W90.4, “Injection into joint NEC”; W90.8, “Other specified puncture of joint”; W90.9, “Unspecified puncture of joint”; W91.1, “Manipulation of joint using traction NEC”; W91.3, “Manipulation of prosthetic joint NEC”; W91.8, “Other specified other manipulation of joint”; W91.9, “Unspecified other manipulation of joint”; W92.1, “Biopsy of lesion of joint NEC”; W92.2, “Distension of joint”; W92.3, “Examination of joint under image intensifier”; W92.4, “Examination of joint under anaesthetic”; W92.5, “Examination of joint NEC”; W92.8, “Other specified other operations on joint”; W92.9, “Unspecified other operations on joint”; O19.8, “Other specified other therapeutic endoscopic operations on other joint structure”; O27.1, “Extra-articular ligament reconstruction for stabilisation of joint”; O27.8, “Other specified other stabilising operations on joint”; O27.9, “Unspecified other stabilising operations on joint”; S57.1, “Debridement of skin NEC”; S57.2, “Removal of slough from skin NEC”; S57.3, “Toilet of skin NEC”; S57.4, “Dressing of skin NEC”; S57.5, “Attention to dressing of skin NEC”; S57.6, “Cleansing and sterilisation of skin NEC”; S57.7, “Dressing of skin using vacuum assisted closure device NEC”; S57.8, “Other specified exploration of other skin of other site”; S57.9, “Unspecified exploration of other skin of other site”; S58.2, “Larvae debridement therapy of skin NEC”; X09.8, “Other specified amputation of leg”; X09.9, “Unspecified amputation of leg”.

<u>Site for revision</u>: Z84.3, “Hip joint”; Z76.1, “Head of femur”; Z75.6, “Acetabulum”; Z84.9, “Joint of pelvis or upper leg NEC”.



For peer review only - http://bmjopen.bmj.com/site/about/guidelines.xhtml

Algorithm: One code from procedure type 1 or a combination of one code from procedure type 2 and site for revision were used to identify hip revision.

For peer review only



For peer review only - http://bmjopen.bmj.com/site/about/guidelines.xhtml

**Appendix 3.** Codes defined in the International Statistical Classification of Diseases and Related Health Problems 10th Revision (ICD-10) that we used to identify complications in the Hospital Episode Statistics (HES) registry.

<u>Stroke</u>: I60.X, “Subarachnoid haemorrhage”; I61.0, “Intracerebral haemorrhage in hemisphere, subcortical”; I61.1, “Intracerebral haemorrhage in hemisphere, cortical”; I61.2, “Intracerebral haemorrhage in hemisphere, unspecified”; I61.3, “Intracerebral haemorrhage in brain stem”; I61.4, “Intracerebral haemorrhage in cerebellum”; I61.5, “Intracerebral haemorrhage, intraventricular”; I61.6, “Intracerebral haemorrhage, multiple localized”; I61.8, “Other intracerebral haemorrhage”; I61.9, “Intracerebral haemorrhage, unspecified”; I63.0, “Cerebral infarction due to thrombosis of precerebral arteries”; I63.1, “Cerebral infarction due to embolism of precerebral arteries”; I63.2, “Cerebral infarction due to unspecified occlusion or stenosis of precerebral arteries”; I63.3, “Cerebral infarction due to thrombosis of cerebral arteries”; I63.4, “Cerebral infarction due to embolism of cerebral arteries”; I63.5, “Cerebral infarction due to unspecified occlusion or stenosis of cerebral arteries”; I63.6, “Cerebral infarction due to cerebral venous thrombosis, nonpyogenic”; I63.8, “Other cerebral infarction”; I63.9, “Cerebral infarction, unspecified”; and I64.X, “Stroke, not specified as haemorrhage or infarction”.

<u>Respiratory infection</u>: J12.X, “Viral pneumonia, not elsewhere classified: bronchopneumonia due to viruses other than influenza viruses”; J13, “Pneumonia due to *Streptococcus pneumoniae*”; J14, “Pneumonia due to *Haemophilus influenzae*”; J15.X, “Bacterial pneumonia, not elsewhere classified: bronchopneumonia due to bacteria other than *S. pneumoniae* and *H. influenzae*”; J18.0, “Bronchopneumonia, unspecified. Excluding bronchiolitis”; J18.1, “Lobar pneumonia, unspecified”; J18.2, “Hypostatic pneumonia, unspecified”; J18.8, “Other pneumonia, organism unspecified”; J18.9, “Pneumonia, unspecified”; J22, “Unspecified acute lower respiratory infection”; J44.0, “Chronic



For peer review only - http://bmjopen.bmj.com/site/about/guidelines.xhtml

obstructive pulmonary disease with acute lower respiratory infection. Excluding with influenza"; J44.1, "Chronic obstructive pulmonary disease with acute exacerbation, unspecified"; J69.0, "Pneumonitis due to food and vomit. Excluding Mendelson syndrome"; J69.1, "Pneumonitis due to oils and essences"; J69.8, "Pneumonitis due to other solids and liquids. Pneumonitis due to aspiration of blood"; and J85.1, "Abscess of lung with pneumonia. Excluding with pneumonia due to specified organism".

Acute myocardial infarction: I21.0, "Acute transmural myocardial infarction of anterior wall"; I21.1, "Acute transmural myocardial infarction of inferior wall"; I21.2, "Acute transmural myocardial infarction of other sites"; I21.3, "Acute transmural myocardial infarction of unspecified site"; I21.4, "Acute subendocardial myocardial infarction"; and I21.9, "Acute myocardial infarction, unspecified".

Pulmonary embolism/deep vein thrombosis: I80.1, "Phlebitis and thrombophlebitis of superficial vessels of lower extremities"; I80.1, "Phlebitis and thrombophlebitis of femoral vein"; I80.3, "Phlebitis and thrombophlebitis of other deep vessels of lower extremities"; I26.0, "Pulmonary embolism with mention of acute cor pulmonale"; and I26.9, "Pulmonary embolism without mention of acute cor pulmonale".

Urinary tract infection: N30.0, "Acute cystitis. Excluding irradiation cystitis and trigonitis"; and N39.0, "Urinary tract infection, site not specified".

Wound disruption: T81.3, "Disruption of operation wound, not elsewhere classified".

Surgical site infection: T81.4, "Infection following a procedure, not elsewhere classified".

Fracture after implant: M96.6, "Fracture of bone following insertion of orthopaedic implant, joint prosthesis, or bone plate. Excluding complication of internal orthopaedic devices, implants or grafts".



For peer review only - http://bmjopen.bmj.com/site/about/guidelines.xhtml

Complication of prosthesis: T84.0, “Mechanical complication of internal joint prosthesis”.

Neurovascular injury: T81.2, “Accidental puncture and laceration during a procedure, not elsewhere classified. Accidental perforation of: blood vessel, nerve or organ by: catheter, endoscope, instrument or probe during a procedure”.

Acute renal failure: N17.0, “Acute renal failure with tubular necrosis”; N17.1, “Acute renal failure with acute cortical necrosis”; N17.2, “Acute renal failure with medullary necrosis”; N17.8, “Other acute renal failure”; and N17.9, “Acute renal failure, unspecified”.

For peer review only



For peer review only - http://bmjopen.bmj.com/site/about/guidelines.xhtml

**Appendix 4.** Operative procedure codes (OPCS 4.8) that we used to identify blood-transfusion complication in the Hospital Episode Statistics (HES) registry.

X33.2, "Intravenous blood transfusion of packed cells"; X33.3, "Intravenous blood transfusion of platelets"; X33.8, "Other specified other blood transfusion"; X33.9, "Unspecified other blood transfusion"; X33.1, "Intra-arterial blood transfusion"; X33.7, "Autologous transfusion of red blood cells"; X34.1, "Transfusion of coagulation factor"; X34.2, "Transfusion of plasma not elsewhere classified"; X34.3, "Transfusion of serum not elsewhere classified"; and X34.4, "Transfusion of blood expander".

For peer review only



For peer review only - http://bmjopen.bmj.com/site/about/guidelines.xhtml

**Appendix 5.** Codes defined in the International Statistical Classification of Diseases and Related Health Problems 10th Revision (ICD-10) that we used to identify comorbidities in the Hospital Episode Statistics (HES) registry.

Myocardial infarction: I21, “Acute myocardial infarction”; and I22, “Subsequent myocardial infarction”.

Congestive heart failure: I50.0, “Congestive heart failure”.

Peripheral vascular disease: I70, “Atherosclerosis”; I71, “Aortic aneurysm and dissection”; I72, “Other aneurysm and dissection”; and I73, “Other peripheral vascular diseases”.

Cerebrovascular disease: I60, “Subarachnoid haemorrhage”; I61, “Intracerebral haemorrhage”; I62, “Other nontraumatic intracranial haemorrhage”; I63, “Cerebral infarction”; I64, “Stroke, not specified as haemorrhage or infarction”; I65, “Occlusion and stenosis of precerebral arteries, not resulting in cerebral infarction”; I66, “Occlusion and stenosis of cerebral arteries, not resulting in cerebral infarction”; and I67, “Other cerebrovascular diseases”.

Dementia: F00, “Dementia in Alzheimer disease”; F01, “Vascular dementia”; F02, “Dementia in other diseases classified elsewhere”; and F03, “Unspecified dementia”.

Chronic obstructive pulmonary disease: J41, “Simple and mucopurulent chronic bronchitis”; J42, “Unspecified chronic bronchitis”; J43, “Emphysema”; J44, “Other chronic obstructive pulmonary disease”; J45, “Asthma”; J46, “Status asthmaticus”; and J47, “Bronchiectasis”.

Connective tissue disease: M05, “Seropositive rheumatoid arthritis”; M06, “Other rheumatoid arthritis”; M08, “Juvenile arthritis”; M15, “Polyarthrosis”; M16, “Coxarthrosis [arthrosis of hip]”; M17, “Gonarthrosis [arthrosis of knee]”; M18, “Arthrosis of first carpometacarpal joint”; M19, “Other arthrosis” ; M35, “Other systemic involvement of



For peer review only - http://bmjopen.bmj.com/site/about/guidelines.xhtml

connective tissue" ; and M36, "Systemic disorders of connective tissue in diseases classified elsewhere".

Peptic ulcer disease: K25, "Gastric ulcer"; K26, "Duodenal ulcer"; K27, "Peptic ulcer, site unspecified"; and K28, "Gastrojejunal ulcer".

Mild liver disease: K70.0, "Alcoholic fatty liver"; K76.0, "Fatty (change of) liver, not elsewhere classified"; and K76.1 "Chronic passive congestion of liver".

Mild diabetes (without end organ damage - include ketoacidosis and coma): E10.X, "Insulin-dependent diabetes mellitus"; E10.0, "Insulin-dependent diabetes mellitus with coma"; E10.1, "Insulin-dependent diabetes mellitus with ketoacidosis" ; E10.9, "Insulin-dependent diabetes mellitus without complications"; E11.X, "Non-insulin-dependent diabetes mellitus"; E11.0, "Non-insulin-dependent diabetes mellitus with coma"; E11.1, "Non-insulin-dependent diabetes mellitus with ketoacidosis" ; E11.9, "Non-insulin-dependent diabetes mellitus without complications"; E12.X, "Malnutrition-related diabetes mellitus"; E12.0, "Malnutrition-related diabetes mellitus with coma"; E12.1, "Malnutrition-related diabetes mellitus with ketoacidosis"; E12.9, "Malnutrition-related diabetes mellitus without complications"; E13.X, "Other specified diabetes mellitus"; E13.0, "Other specified diabetes mellitus with coma"; E13.1, "Other specified diabetes mellitus with ketoacidosis"; E13.9, "Other specified diabetes mellitus without complications"; E14.X, "Unspecified diabetes mellitus"; E14.0, "Unspecified diabetes mellitus with coma"; E14.1, "Unspecified diabetes mellitus with ketoacidosis"; and E14.9, "Unspecified diabetes mellitus without complications" .

Hemiplegia: G81, "Hemiplegia. Excluding congenital cerebral palsy"



For peer review only - http://bmjopen.bmj.com/site/about/guidelines.xhtml

Moderate/severe renal disease: N17, “Acute renal failure”; N18, “Chronic kidney disease”; and N19, “Unspecified kidney failure”.

Severe diabetes (i.e. with organ damage): E10, “Insulin-dependent diabetes mellitus”; E11, “Non-insulin-dependent diabetes mellitus”; E12, “Malnutrition-related diabetes mellitus”; E13, “Other specified diabetes mellitus”; or E14, “Unspecified diabetes mellitus”. Complicated with: .2, “With renal complications”; .3 “With ophthalmic complications”; .4, “With neurological complications”; .5, “With peripheral circulatory complications”; .6, “With other specified complications”; .7, “With multiple complications”; or .8, “With unspecified complications”. N083, “Glomerular disorders in diabetes mellitus”.

Tumour: C00-C75, “Malignant neoplasms, stated or presumed to be primary, of specified sites, except of lymphoid, haematopoietic and related tissue”; C76, “Malignant neoplasm of other and ill-defined sites”; C80, “Malignant neoplasm, without specification of site”; C88, “Malignant immunoproliferative diseases”; C90.0, “Multiple myeloma”; C90.2, “Extramedullary plasmacytoma”; C96, “Other and unspecified malignant neoplasms of lymphoid, haematopoietic and related tissue”; C97, “Malignant neoplasms of independent (primary) multiple sites”; D00-D09, “In situ neoplasms”; D10-D36, “Benign neoplasms”; and D37-D48, “Neoplasms of uncertain or unknown behaviour”.

Leukaemia: C90.1, “Plasma cell leukaemia”; C91, “Lymphoid leukaemia”; C92, “Myeloid leukaemia”; C93, “Monocytic leukaemia”; C94, “Other leukaemias of specified cell type”; and C95 “Leukaemia of unspecified cell type”.

Lymphoma: C81, “Hodgkin lymphoma”; C82, “Follicular lymphoma”; C83, “Non-follicular lymphoma”; C84, “Mature T/NK-cell lymphomas”; and C85, “Other and unspecified types of non-Hodgkin lymphoma”.



For peer review only - http://bmjopen.bmj.com/site/about/guidelines.xhtml

Moderate/severe liver disease: K70, “Alcoholic liver disease except”; K71, “Toxic liver disease”; K72, “Hepatic failure, not elsewhere classified”; K73, “Chronic hepatitis, not elsewhere classified”; K74, “Fibrosis and cirrhosis of liver”; K75, “Other inflammatory liver diseases”; and K76, “Other diseases of liver”. Excluding codes for mild liver disease: K70.0, K76.0 and K76.1.

AIDS: B20, “HIV disease resulting in infectious and parasitic diseases”; B21, “HIV disease resulting in malignant neoplasms”; B22, “HIV disease resulting in other specified diseases”; and B23 “HIV disease resulting in other conditions”.

Metastatic solid tumour: C77, “Secondary and unspecified malignant neoplasm of lymph nodes”; C78, “Secondary malignant neoplasm of respiratory and digestive organs”; and C79, “Secondary malignant neoplasm of other and unspecified sites”.

For peer review only

STROBE Statement—Checklist of items that should be included in reports of ***cohort studies***

| | Item No | Checked and present | Recommendation |
|---|---|---|---|
| **Title and abstract** | 1 | Page 3 abstract | (*a*) Indicate the study's design with a commonly used term in the title or the abstract |
| | | Page 3 | (*b*) Provide in the abstract an informative and balanced summary of what was done and what was found |
| **Introduction** | | | |
| Background/rationale | 2 | Pages 5-6 | Explain the scientific background and rationale for the investigation being reported |
| Objectives | 3 | Page 6 | State specific objectives, including any prespecified hypotheses |
| **Methods** | | | |
| Study design | 4 | Pages 9-10 | Present key elements of study design early in the paper |
| Setting | 5 | Page 6 | Describe the setting, locations, and relevant dates, including periods of recruitment, exposure, follow-up, and data collection |
| Participants | 6 | Page 9 | (*a*) Give the eligibility criteria, and the sources and methods of selection of participants. Describe methods of follow-up |
| | | NA | (*b*) For matched studies, give matching criteria and number of exposed and unexposed |
| Variables | 7 | Pages 7-8 | Clearly define all outcomes, exposures, predictors, potential confounders, and effect modifiers. Give diagnostic criteria, if applicable |
| Data sources/ measurement | 8* | Pages 6-9 | For each variable of interest, give sources of data and details of methods of assessment (measurement). Describe comparability of assessment methods if there is more than one group |
| Bias | 9 | Pages 9 | Describe any efforts to address potential sources of bias |
| Study size | 10 | Page 9 | Explain how the study size was arrived at |
| Quantitative variables | 11 | Page 7-8 | Explain how quantitative variables were handled in the analyses. If applicable, describe which groupings were chosen and why |
| Statistical methods | 12 | Page 10-11 | (*a*) Describe all statistical methods, including those used to control for confounding |
| | | Page 10-11 | (*b*) Describe any methods used to examine subgroups and interactions |
| | | Page 9 | © Explain how missing data were addressed |
| | | NA | (*d*) If applicable, explain how loss to follow-up was addressed |
| | | NA | (*e*) Describe any sensitivity analyses |
| **Results** | | | |
| Participants | 13* | Page 12, figure 1 | (a) Report numbers of individuals at each stage of study—eg numbers potentially eligible, examined for eligibility, confirmed eligible, included in the study, completing follow-up, and analysed |
| | | Figure 1 | (b) Give reasons for non-participation at each stage |

For peer review only

| | | | |
|---|---|---|---|
| | | Figure 1 | © Consider use of a flow diagram |
| Descriptive data | 14* | Page 12 | (a) Give characteristics of study participants (eg demographic, clinical, social) and information on exposures and potential confounders |
| | | Page 13, Figure 1, Supplementary Tables S4-S5 | (b) Indicate number of participants with missing data for each variable of interest |
| | | Page 15 | © Summarise follow-up time (eg, average and total amount) |
| Outcome data | 15* | Pages 13-15 | Report numbers of outcome events or summary measures over time |
| Main results | 16 | Pages 13-15, Table 1 Supplementary Tables S1-S3 and S6-S7 | (*a*) Give unadjusted estimates and, if applicable, confounder-adjusted estimates and their precision (eg, 95% confidence interval). Make clear which confounders were adjusted for and why they were included |
| | | Pages 13-15, Table 1 Supplementary Tables S1-S3 and S6-S7 | (*b*) Report category boundaries when continuous variables were categorized |
| | | NA | © If relevant, consider translating estimates of relative risk into absolute risk for a meaningful time period |
| Other analyses | 17 | Figures 3 and 4 | Report other analyses done—eg analyses of subgroups and interactions, and sensitivity analyses |
| **Discussion** | | | |
| Key results | 18 | Page 15 | Summarise key results with reference to study objectives |
| Limitations | 19 | Pages 15-16 | Discuss limitations of the study, taking into account sources of potential bias or imprecision. Discuss both direction and magnitude of any potential bias |
| Interpretation | 20 | Pages 17-19 | Give a cautious overall interpretation of results considering objectives, limitations, multiplicity of analyses, results from similar studies, and other relevant evidence |
| Generalisability | 21 | Pages 17-19 | Discuss the generalisability (external validity) of the study results |
| **Other information** | | | |
| Funding | 22 | Page 20 | Give the source of funding and the role of the funders for the present study and, if applicable, for the original study on which the present article is based |

*Give information separately for exposed and unexposed groups.

**Note:** An Explanation and Elaboration article discusses each checklist item and gives methodological background and published examples of transparent reporting. The STROBE checklist is best used in conjunction with this article (freely available on the Web sites of PLoS Medicine at http://www.plosmedicine.org/, Annals of Internal Medicine at http://www.annals.org/, and Epidemiology at http://www.epidem.com/). Information on the STROBE Initiative is available at http://www.strobe-statement.org.

For peer review only

BMJ Open

# Assessment on Patient Outcomes of Primary Hip Replacement: an Interrupted Time Series Analysis from "The National Joint Registry of England and Wales"

| | |
|---|---|
| Journal: | *BMJ Open* |
| Manuscript ID | bmjopen-2019-031599.R1 |
| Article Type: | Original research |
| Date Submitted by the Author: | 10-Sep-2019 |
| Complete List of Authors: | Garriga, Cesar; University of Oxford, Nuffield Department of Orthopaedics, Rheumatology and Musculoskeletal Sciences (NDORMS); University of Oxford, Centre for Statistics in Medicine, Nuffield Department of Orthopaedics, Rheumatology and Musculoskeletal Sciences<br>Murphy, Jacqueline; University of Oxford, Nuffield Department of Orthopaedics, Rheumatology and Musculoskeletal Sciences (NDORMS); University of Oxford, Nuffield Department of Population Health<br>Leal, Jose; University of Oxford, Nuffield Department of Population Health<br>Arden, Nigel; University of Oxford, Nuffield Department of Orthopaedics, Rheumatology and Musculoskeletal Sciences (NDORMS); University of Southampton, MRC Lifecourse Epidemiology Unit<br>Price, Andrew; University of Oxford, Nuffield Department of Orthopaedics, Rheumatology and Musculoskeletal Sciences<br>Prieto-Alhambra, Daniel; University of Oxford Nuffield Department of Orthopaedics Rheumatology and Musculoskeletal Sciences; University of Oxford, Centre for Statistics in Medicine, Nuffield Department of Orthopaedics, Rheumatology and Musculoskeletal Sciences<br>Carr, Andrew; University of Oxford, Nuffield Department of Orthopaedics, Rheumatology and Musculoskeletal Sciences (NDORMS); University of Oxford, Centre for Statistics in Medicine, Nuffield Department of Orthopaedics, Rheumatology and Musculoskeletal Sciences<br>Rangan, Amar; University of Oxford, Nuffield Department of Orthopaedics, Rheumatology and Musculoskeletal Sciences (NDORMS); University of York, Department of Health Sciences<br>Cooper, Cyrus; University of Oxford, Nuffield Department of Orthopaedics, Rheumatology and Musculoskeletal Sciences (NDORMS); University of Southampton, MRC Lifecourse Epidemiology Unit<br>Peat, George; Keele University, Primary Care & Health Sciences<br>Fitzpatrick, Ray; University of Oxford, Nuffield Department of Population Health<br>Barker, Karen L.; University of Oxford, Nuffield Department of Orthopaedics, Rheumatology and Musculoskeletal Sciences (NDORMS); Oxford University Hospitals NHS Foundation Trust Nuffield Orthopaedic Centre<br>Judge, Andrew; University of Oxford, Nuffield Department of Orthopaedics, Rheumatology and Musculoskeletal Sciences (NDORMS); |

| | |
|---|---|
| | University of Bristol, Musculoskeletal Research Unit, Translational Health Sciences, Bristol Medical School |
| <b>Primary Subject Heading</b>: | Health services research |
| Secondary Subject Heading: | Epidemiology, Health economics, Health services research, Surgery, Rheumatology |
| Keywords: | Hip < ORTHOPAEDIC & TRAUMA SURGERY, EPIDEMIOLOGY, HEALTH ECONOMICS, HEALTH SERVICES ADMINISTRATION & MANAGEMENT |
| | |

SCHOLARONE™
Manuscripts

**Title:** Assessment on Patient Outcomes of Primary Hip Replacement: an Interrupted Time Series Analysis from "The National Joint Registry of England and Wales"

**Author names:** Cesar Garriga[1,2], Postdoctoral researcher (PhD, cesar.garriga-fuentes@ndorms.ox.ac.uk), Jacqueline Murphy[1,3], Researcher (MMath, jacqueline.murphy@qmul.ac.uk), Jose Leal[3], University Research Lecturer (DPhil, jose.leal@dph.ox.ac.uk), Nigel Arden[1,4], Professor in Rheumatic Diseases (Prof, nigel.arden@ndorms.ox.ac.uk), Andrew Price[1], Professor of Orthopaedic Surgery (Prof, andrew.price@ndorms.ox.ac.uk), Daniel Prieto-Alhambra[1,2,4], Professor of Pharmaco- and Device Epidemiology (Prof, daniel.prietoalhambra@ndorms.ox.ac.uk), Andrew Carr[1,2], Nuffield Professor of Orthopaedic Surgery (Prof, andrew.carr@ndorms.ox.ac.uk), Amar Rangan[1,5], Professor of Orthopaedic Surgery (Prof, Amar.Rangan@york.ac.uk), Cyrus Cooper[1,4], Professor of Rheumatology (Prof, cc@mrc.soton.ac.uk), George Peat[6], Professor of Clinical Epidemiology (Prof, g.m.peat@keele.ac.uk), Raymond Fitzpatrick[3], Professor of Public Health and Primary Care (Prof, raymond.fitzpatrick@nuffield.ox.ac.uk), Karen Barker[1,7], Professor of Physiotherapy (Prof, karen.barker@ouh.nhs.uk), Andrew Judge[1,2,4,8], Professor of Translational Statistics (Prof, andrew.judge@ndorms.ox.ac.uk)

[1] Nuffield Department of Orthopaedics, Rheumatology and Musculoskeletal Sciences, Nuffield Orthopaedic Centre, University of Oxford, Windmill Road, Oxford, OX3 7LD, UK.

[2] Centre for Statistics in Medicine, Nuffield Department of Orthopaedics, Rheumatology and Musculoskeletal Sciences, Nuffield Orthopaedic Centre, University of Oxford, Windmill Road, Oxford, OX3 7LD, UK.

[3] Nuffield Department of Population Health, University of Oxford, Richard Doll Building, Old Road Campus, Oxford, OX3 7LF, UK.

For peer review only

[4] MRC Lifecourse Epidemiology Unit, University of Southampton, Southampton General Hospital, Southampton, SO16 6YD, UK.

[5] Department of Health Sciences, University of York, Seebohm Rowntree Building, Heslington, York YO10 5DD

[6] Primary Care & Health Sciences, Keele University, Keele, Staffordshire ST5 5BG, UK.

[7] Physiotherapy Research Unit, Nuffield Orthopaedic Centre, Windmill Rd, Headington, Oxford, OX3 7HE, UK.

[8] Musculoskeletal Research Unit, Translational Health Sciences, Bristol Medical School, University of Bristol, Learning and Research Building, Level 1, Southmead Hospital, Bristol, BS10 5NB, UK.

**Corresponding Author**: Cesar Garriga; Botnar Research Centre; Nuffield Department of Orthopaedics, Rheumatology and Musculoskeletal Sciences; University of Oxford; Windmill Road, Headington; Oxford, OX3 7LD, UK; Tel. +441865 737859; F +44 (0)1865 227 966.

Email: cesar.garriga-fuentes@ndorms.ox.ac.uk.

For peer review only

**STRUCTURED ABSTRACT**

**Objectives**: Effects of the UK Department of Health's national Enhanced Recovery After Surgery (ERAS) Programme on outcomes after primary hip replacement.

**Design:** Natural experimental study using interrupted time-series to assess the changes in trends before, during, and after ERAS implementation (April 2009 to March 2011).

**Setting:** Surgeries in the UK National Joint Registry were linked with Hospital Episode Statistics containing inpatient episodes from NHS trusts in England and patient-reported outcome measures.

**Participants**: Patients aged ≥18 years from 2008 to 2016.

**Main outcome measures:** Regression coefficients of monthly means of length of hospital stay, bed day cost, change in Oxford hip scores (OHS) 6 months post-surgery, complications 6 months post-surgery, and revision rates 5 years post-surgery.

**Results:** 438,921 primary hip replacements were identified. Hospital stays shortened from 5.6 days in April 2008 to 3.6 in December 2016. There were also improvements in bed day costs (£7573 in April 2008 to £5239 in December 2016), positive change in self-reported OHS from baseline to 6 months post-surgery (17.7 points in April 2008 to 22.9 points in December 2016), complication rates (4.1 % in April 2008 to 1.7 % March 2016), and 5-year revision rates (5.9 per 1000 implant-years (95% CI: 4.8 to 7.2) in April 2008 to 2.9 (95% CI:



For peer review only - http://bmjopen.bmj.com/site/about/guidelines.xhtml

2.2 to 3.9) in December 2011). The positive trends in all outcomes started before ERAS was implemented and continued during and after the programme**.**

**Conclusions:** Patient outcomes after hip replacement have improved over the last decade. A national ERAS programme maintained this improvement but did not alter the existing rate of change.

**Strengths and limitations of this study**

- Our study design is a "natural experiment" which controlled for unobservable sources of confounding. This approach for evaluating complex interventions allows for causal inferences without randomised controlled experiments.
- Routinely collected data provided actual-practice information on trends in length of stay, patient reported outcome measures of hip pain and function, complications, and revision surgeries following primary hip replacement procedures.
- A limitation is the variation in interpretation and adoption across centres because what constitutes enhanced recovery after surgery (ERAS) was not clearly established after the expected identification of best practices in the first year of the ERAS programme.

For peer review only



For peer review only - http://bmjopen.bmj.com/site/about/guidelines.xhtml

**INTRODUCTION**

Osteoarthritis represents a significant, and growing, population health burden worldwide[1]. In the United Kingdom (UK), over one million adults aged over 45 years consult general practice for osteoarthritis each year[2]. Osteoarthritis accounts for £3.2 billion in lost productivity in the UK[3], with total direct and indirect costs equivalent to 0.25-0.50% of the gross domestic product[4 5]. Almost 88,000 primary hip replacement operations were undertaken during 2016 in the National Health Service (NHS), over 90% of which were for osteoarthritis. This number continues to increase[6].

Between April 2009 and March 2011, the UK Department of Health implemented an Enhanced Recovery After Surgery (ERAS) Partnership Programme[7] to improve recovery in major planned colorectal, musculoskeletal, gynaecological, and urological surgical pathways. The first year of the programme focused on learning best practice from pioneer units of ERAS practice in the NHS. ERAS has a series of evidence-based care elements that all support recovery by reducing the bodily stress reactions caused by injury during surgery. These reductions in the stress responses are of particular importance for the vulnerable patient with co-morbidities, who is often also frail and elderly.[8] It collected information about principles of enhanced recovery, clinical elements of the patient pathway, metrics, and success factors. It established a website to share information and resources, generated a financial and equality impact evaluation, published an implementation guide, and developed an online reporting tool to support implementation. A lead for enhanced recovery was named in each local health authority to prepare for the spread of the ERAS programme across the NHS during the implementation phase in the programme's second year.



For peer review only - http://bmjopen.bmj.com/site/about/guidelines.xhtml

The ERAS in musculoskeletal care focused on planned hip and knee replacement surgeries. ERAS is a complex intervention[9 10] to improve several areas of care across a patient's pathway through surgery. Pre-operatively, ERAS aims for the patient to be in the best possible condition for surgery. In primary care haemoglobin levels and pre-existing co-morbidities like diabetes are assessed. At the hospital nurses test cardiopulmonary exercise and appropriate anaesthetic for covering surgery is evaluated. In addition, there is informed decision making after offering to the patient information and managing her/his expectations. At the admission, hospitals arrange to admit patients the same day of surgery, fluid hydration is optimised using i oral complex carbohydrates to reduce patient anxiety, reduce the body's resistance to insulin and inflammatory response. Peri-operatively, ERAS aims to give the patient the best possible management during and after surgery[11]. It includes: minimally invasive surgery if possible, individualised fluid therapy, avoid crystalloid overload, use of regional/spinal and local anaesthetic with sedation, and hypothermia prevention. Post-operatively, ERAS aims to give the patient the best possible rehabilitation. It includes: no routine use of wound drains and/or nasogastric tubes, active, planned mobilisation within 24 hours, early oral hydration and nutrition, intravenous therapy stopped early, catheters removed early, oral analgesia avoiding systemic opiates where possible. Follow-up covers: discharge on planned day or when criteria met, therapy support (stoma, physiotherapy, dietitian…) and 24 hour telephone follow-up if appropriate. Patients are given information before and after surgery, such as changes to make around the home, strengthening exercises, and changes to nutrition. ERAS aims to enable earlier return home from hospital with tailored discharge, when suitable for the patient.

Despite their widespread implementation, there is limited evidence about the effectiveness of ERAS programmes in hip and knee replacement surgery[12]. Length of hospital stay has been



For peer review only - http://bmjopen.bmj.com/site/about/guidelines.xhtml

declining prior to the intervention, and we hypothesised that after the implementation of ERAS, this downward secular trend would decline faster. For the outcomes of complications, revision, pain and function, we did not have a specific a-prior hypothesis as it is unclear what impact ERAS would have on these outcomes. We aimed to determine whether implementing ERAS in hip replacement has led to improved patient outcomes and shorter hospital stays.

## METHODS

### Data source

We used data from the UK National Joint Registry (NJR), which contains data on hip replacement surgeries from all English and Welsh hospitals. It includes 2 million patients since 2003 and covers 95% and 91% of primary hip replacements and revisions, respectively[13].

### Data linkages

Primary operations were linked with Hospital Episode Statistics (HES) data, which contain records of all NHS funded inpatient episodes undertaken in NHS trusts in England (125 million each year). Planned hip replacements were linked to patient-reported outcome measures (PROMs). Patients funded by the NHS in England are asked to complete questionnaires before and 6 months after surgery to evaluate their perceived improvement in health. We retrieved a cohort of patients undergoing planned hip replacement in England between April 2008 and December 2016. Mortality data were matched to the Office for National Statistics database.

### Outcome measures

For peer review only



For peer review only - http://bmjopen.bmj.com/site/about/guidelines.xhtml

We evaluated trends in hospital stay length for patients undergoing primary hip replacement. Length of stay was calculated as the number of days between the hospital admission and discharge dates. We used the same set of patients to estimate the inpatient cost for the index episode using NHS reference costs from 2015/16[14]. We estimated the mean cost per bed day based on each patient's healthcare resource use and length of hospital stay. Appendix 1 further explains the cost methods.

We also assessed the absolute change in the Oxford hip score (OHS), a PROM. Patients complete the same questionnaire about their hip pain and function before and 6 months after surgery[15]. Each question is scored between 0 (worst symptoms) and 4 (least symptoms). The scores from the 12 questions are summed to give a total score between 0 (worst) and 48 (best). We calculated the difference between the total scores 6 months post-surgery and at baseline to obtain a measure of change associated with surgery. Higher positive values for OHS change represent greater self-reported improvement in pain and function.

We calculated the 6 months post-surgery complication proportions. We defined post-operative complications as one or more events happening up to 6 months after primary hip replacement: stroke (excluding transient ischaemic attack), respiratory infection, acute myocardial infarction, pulmonary embolism/deep vein thrombosis, urinary tract infection, wound disruption, surgical site infection, fracture after implant, complication of prosthesis, neurovascular injury, acute renal failure, or blood transfusion. We identified these complications in HES data using diagnosis codes from the "International Statistical Classification of Diseases and Related Health Problems 10th Revision" (ICD-10) (Appendix 2), except for blood transfusion, for which we used the "Classification of Interventions and Procedures version 4" (OPCS-4) codes (Appendix 3).



For peer review only - http://bmjopen.bmj.com/site/about/guidelines.xhtml

We evaluated the rate of revision surgery up to 5 years after primary hip replacement. We included revisions declared to the NJR registry by surgeons[16] and reported to HES using codes from Appendix 4. We specified our analysis time in years, reporting the rate as the number of revisions per 1000 implant-years.

**Intervention**

The ERAS programme for hip replacement surgery was implemented nationally between April 2009 and March 2011. During the first year the programme focused on identifying best practice, determining clinical elements of the patient pathway, publishing an implementation guide, supporting early adopters of the programme to better understand key factors for implementation and sustainability [17]. During the second year ERAS supported local health areas for delivering and commissioning implementation of ERAS.

**Potential modifiers**

We evaluated whether trends in hospital stay length and OHS change after surgery differed by age at the time of primary hip replacement (18-59, 60-69, 70-79, 80-84, or ≥85 years) and presence of co-morbidities according to the Charlson classification[18] (no comorbidities versus one or more comorbidities): myocardial infarction, congestive heart failure, peripheral vascular disease, cerebrovascular disease, dementia, chronic obstructive pulmonary disease, connective tissue disease, peptic ulcer disease, mild liver disease, mild diabetes, hemiplegia, moderate/severe renal disease, severe diabetes (i.e., with organ damage), tumour, leukaemia, lymphoma, moderate/severe liver disease, AIDS, and metastatic solid tumour. The ICD-10 codes used are listed in Appendix 5.



For peer review only - http://bmjopen.bmj.com/site/about/guidelines.xhtml

**Participants and inclusion criteria**

We included patients receiving planned hip replacement surgery (Fig. 1) between 1 April 2008 and 31 December 2016. We excluded patients without a concordant date of replacement between NJR and HES registries.

When analysing length of stay, we also excluded patients staying more than 15 days in hospital and patients with a missing hospital stay length or a hospital discharge date before their hospital admission date. When analysing change in OHS, we also excluded patients missing baseline and/or 6-month follow-up OHS scores. When analysing complications, we also excluded patients with complications before the surgery date and those with surgery after June 2016 to guarantee all patients had at least 6 months of follow-up. When analysing 5-year revision rates, we excluded patients who received surgery after 2011 to ensure all patients had at least 5 years of follow-up.

**Missing data**

We excluded patients with missing length of hospital stay or missing OHS data from the respective analyses. We used Pearson's $\chi^2$ statistic to compare missingness in the OHS measure before, during, and after ERAS implementation (April 2008 - March 2009, April 2009 - March 2011, and April 2011 - December 2016), across patient age (18-59, 60-69, 70-79, 80-84, and ≥85 years), and in the presence and absence of co-morbidities. We also compared the distribution of patients with and without OHS data by study period, patient age, and presence of co-morbidities.

**Study design and statistical analysis**

For peer review only



For peer review only - http://bmjopen.bmj.com/site/about/guidelines.xhtml

We used a natural experimental study design[19]. We evaluated the effect of ERAS on trends before, during, and after its implementation [20 21]. Trusts could choose when to start implementing the programme within the implementation period (April 2009 to March 2011), and we assumed that they only implemented the programme within this period. We described the trends by calculating monthly outcomes as means (length of stay, bed costs, change in OHS), proportions (complications), or rates (revision), and their 95% confidence intervals (CI). We estimated a fractional polynomial over the study period and plotted the resulting curve with the CI of the mean.

We used an interrupted time series approach to estimate changes in outcomes during and immediately after the intervention period, while controlling for baseline levels and trends. We modelled aggregated data points of each outcome of interest by month using segmented linear regression[21]:

$$Y_t = \beta_0 + (\beta_1 * \text{time}_t) + (\beta_2 * \text{ERAS}_0) + (\beta_3 * \text{time after ERAS}_0) + (\beta_4 * \text{ERAS}_{end}) + (\beta_5 * \text{time after ERAS}_{end}) + e_t.$$

Yt is the mean of each outcome for patients undergoing primary hip replacements – mean days in hospital for length of stay, mean OHS change for the PROM analysis, mean proportion of complications for 6-month complications outcome, and mean rate of revisions of primary hip replacements for the 5-year revision outcome – in month t. "Time" is a continuous variable representing the number of months from the start of the observation period (April 2008) until time t. $\beta_0$ estimates the baseline level of the outcome at the beginning of the time series (April 2008). $\beta_1$ estimates the trend before ERAS was implemented in April 2009. $\beta_2$ is the change in level immediately after the intervention



For peer review only - http://bmjopen.bmj.com/site/about/guidelines.xhtml

($ERAS_0$ = April 2009). $\beta_3$ estimates the change in the trend in the monthly mean (number or rate, depending on outcome) after ERAS started (i.e., ERAS implementation trend). $\beta_4$ is the change in level immediately after the end of the intervention ($ERAS_{end}$ = March 2011). $\beta_5$ estimates the change in the trend in the mean monthly number or rate (depending on outcome) after ERAS ended.

We excluded non-significant terms using a backward approach to maximise statistical power, producing a parsimonious model with meaningful selected variables. In preliminary analysis we checked for autocorrelation with the previous month, two months…twelve months using Durbin's alternative test[22]. As autocorrelation invalidated the interpretation of the model, we estimated the linear regression models with Newey-West standard errors[23]. Parsimonious models were generated using the variables previously selected in the backward regression. We also report the full models.

All analyses were conducted using Stata version 13.1 statistical software (StataCorp, College Station, Texas). We followed the STROBE (Strengthening the Reporting of Observational Studies in Epidemiology) guideline in reporting this study[24].

**Patient and public involvement**

The James Lind Alliance has identified the need for involving patients in identifying outcomes that matter to them (patient-identified outcomes). We conducted a patient forum with the PEP-R group to identify outcomes from those available in the routine datasets available for this study. The top outcomes were: 1) pain and function, 2) complications (particularly hospital-acquired infection), 3) length of stay (dependent on the level of support

For peer review only



For peer review only - http://bmjopen.bmj.com/site/about/guidelines.xhtml

at home), 4) revision surgery, and 5) mortality (rated low importance) (further detail is provided in Supplementary Text S1).

## RESULTS

We identified 438,921 planned primary hip replacements between April 2008 and December 2016 (Fig. 1). Sixty percent of patients were women and the average age was 69 years (SD ±11 years). The mean body mass index (BMI) at primary surgery of 28.9 kg/m$^2$ (SD ±5.2 kg/m$^2$) fell into the overweight category [25], although BMI was missing for 28% of patients. Most patients (84%) had a physical status[26] of "mild" or "fit".

### Length of stay

Hospital stays shortened from 5.6 days (95% CI: 5.5 to 5.7) in April 2008 to 3.6 (95% CI: 3.6 to 3.7) in December 2016 (Fig. 2A). As shown in Table 1, they were already shortening significantly by -0.020% every month (95% CI: -0.023% to -0.017%) before ERAS (full models in Supplementary Table S1). Hospital stay shortened more quickly (-0.033%) during the implementation period (April 2009 to March 2011), then declined at a slower rate (-0.002%) once ERAS ended (April 2011 to December 2016).

Although older patients had longer hospital stays, all age groups shared the shortening stay trend (Fig. 3, Supplementary Tables S2 and S3). For example, those aged 18-59 years decreased from 4.7 days (95% CI: 4.6 to 4.9) in April 2008 to 3.0 days (95% CI: 2.9 to 3.1) in December 2016 and those aged ≥85 years decreased from 8.1 days (95% CI: 7.7 to 8.6) in April 2008 to 5.3 days (95% CI: 4.9 to 5.6) in December 2016. Patients with and without pre-existing co-morbidity also had shortening hospital stays (Fig. 4).



For peer review only - http://bmjopen.bmj.com/site/about/guidelines.xhtml

We estimated cost data for 432,143 patients. The mean inpatient bed day cost over time showed a similar trend to that observed for hospital stay length. The overall mean cost of the index hospital episode decreased from £7573 (95% CI: £7477 to £7668) in April 2008 to £5239 (95% CI: £5171 to £5306) in December 2016 (Supplementary Fig. S1).

**OHS change**

We excluded 48% of patients with missing OHS information from the change in OHS analysis (Fig. 1). We found more missing OHS data before the intervention (89.7%) than during (41.9%) or after (45.0%) the intervention (Supplementary Table S4). Supplementary Table S5 shows more patients without OHS change data than with this data in the period prior to ERAS (16.2% and 1.7%, respectively).

Self-reported OHS scores improved over the study period, with an increase in OHS 6 months after surgery of 17.7 points (95% CI: 16.4 to 19.0) in April 2008, to 22.9 points (95% CI: 21.8 to 23.9) in December 2016 (Fig. 2B). This trend was also seen in patients with and without co-morbidities and in all age groups, except in those aged ≥85 years, whose change in OHS remained stable over the study period (Supplementary Figures S2 and S3, Supplementary Tables S6 and S7).

According to the interrupted time-series model (Table 1), OHS change increased significantly by 0.158% (95% CI: 0.130% to 0.186%) every month before the intervention (see Supplementary Table S1 for the full model). During ERAS implementation (April 2009 to March 2011), the increase continued but slowed to 0.027%. Change in OHS then became stable after ERAS implementation ended (April 2011 to December 2016).

For peer review only



For peer review only - http://bmjopen.bmj.com/site/about/guidelines.xhtml

**Complications at 6 months**

6,232 (1.6%) patients with a primary hip replacement between April 2008 and March 2016 had one or more complication in the 6 months after surgery. The proportion of complications at 6 months decreased from 4.1% (95% CI: 3.4 to 4.7) to 1.7% (95% CI: 1.3 to 2.1) over the study period (Fig. 2C). According to the interrupted time-series model, complications at 6 months decreased by -0.078% (95% CI: -0.096% to -0.071%) every month before the intervention (Table 1). During the intervention, the trend reversed and complications increased by 0.078%. The proportion of complications stabilised after the intervention ended.

**Five-year revision rates**

According to the NJR registry, 3,392 (2.1%) patients with a primary hip replacement between April 2008 and December 2011 had a hip revision in the following 5 years. We found 840 more 5-year revisions using HES, giving a total of 4,232 (2.6%). Rates of 5-year hip revision decreased from 7.6 per 1000 implant-years (95% CI: 6.4 to 9.2) at risk in April 2008 to 3.8 (95% CI: 3.0 to 4.9) in December 2011 (Fig. 2D).

According to a parsimonious model of 5-year hip-revision rates (Table 1), there was a significant downward trend of -0.098 per 1000 implant-years (95% CI: -0.108 to -0.087) before the implementation of the intervention (April 2009 to March 2011) (see Supplementary Table S1 for the full model). The trend reversed after the intervention ended (April 2011 to December 2016) to increase 0.091 per 1000 implant-years (95% CI: 0.052 to 0.129).

**DISCUSSION**

For peer review only

We have shown that primary hip replacement outcomes have been improving since 2008, with decreasing length of hospital stay, estimated average inpatient bed day costs, complications, and 5-year revision risk, and improving patient-reported pain and function. These positive trends were seen across all age groups and in those with and without co-morbidity, and began before the NHS ERAS programme was implemented. Hospital stays have shortened without adversely affecting patient outcomes.

We hypothesised that the national ERAS intervention would improve primary hip replacement outcomes by changing trends during and after its implementation. Our hypothesis was not confirmed, as ERAS did not influence existing trends. However, we collected only one year of data before the intervention was implemented (April 2008 to March 2009), in comparison with two years of data during implementation (April 2009 to March 2011) and five years of data post-intervention (April 2011 to December 2016). We know from other UK studies that length of stay has been in gradual decline in the years prior to 2008, where Burn et al found that in 1997 mean hospital stays for total hip replacement was 14.28 days, and in 2008, before the ERAS intervention, 7.94 days[27]. Although our study design controlled for unobservable sources of confounding, we stratified by age and presence/absence of comorbidities at surgery to detect patterns in outcome variation. This approach for evaluating complex interventions allows for strong causal inferences without randomised controlled experiments [10 20].

For this "natural experiment," we assumed that ERAS was implemented homogenously across all England NHS trusts in the 2-year implementation period. This assumption is unlikely to be true. The shortening hospital stays and improved outcomes seen before April 2009 may reflect some trusts implementing ERAS elements before the national programme



For peer review only - http://bmjopen.bmj.com/site/about/guidelines.xhtml

began. Shortening hospital stays before ERAS may also reflect attempts to improve the cost-effectiveness of hip replacement surgery, which is an important expenditure for the NHS [27-29].

The hospitals implemented ERAS on different dates, and some had not implemented ERAS by March 2011[17]. The Department of Health surveyed trusts on their use of ERAS near the end of the implementation period (February 2011) and reported full implementation by 81 consultant teams, partial implementation by about 20 teams, and plans to implement ERAS by about 30 teams. As the Department of Health did not clearly describe ERAS after best practices were identified in the first year of the programme[30], there is also variation in the teams' interpretation and adoption of the programme. The Department of Health guideline did not indicate how long ERAS should be implemented for and its later report did not measure actual implementation periods in each hospital[17 30]. Considering the complexity of the intervention and stakeholders involved, implementation length is likely to have varied between hospitals. Our quasi-experimental approach smooths the potential dissimilarities.

Existing systematic reviews and randomised clinical trials have found that ERAS programmes for planned colorectal, joint replacement, and other surgeries reduced hospital stays when compared with conventional care[12]. Many kinds of ERAS programmes for hip replacement have been investigated: physical therapy the same day of the surgery in the recovery room[31]; preoperative patient education, postoperative multimodal analgesia with periarticular injections, early physiotherapy and rehabilitation, and discharge home with an outpatient rehabilitation programme[32 33]; patient and staff education on "enhanced recovery" principles, pre-admission medication, perioperative urinary catheterisation, low-dose spinal



For peer review only - http://bmjopen.bmj.com/site/about/guidelines.xhtml

anaesthesia, and aiming for same-day mobilisation[34]; perioperative information, pain relief, nausea control, nutrition, mobilisation, and elimination[35]; preoperative patient seminar, treatment of pain (spinal anaesthesia) and early mobilisation, standardised programme in the operating theatre (tranexamic acid and no drains), 1-2 hours of a multimodal fast-track rehabilitation regime, daily physiotherapy within the first 24 hours, and a multimodal oral opioid-sparing analgesia[36]; or a perioperative analgesic blocking the peripheral nerve[37]. However, these studies involved small sample sizes (170, 57, 1256, 630, 28, 98, 15)[31-37], were limited to one hospital or trust, and only compared their intervention with traditional management. They cannot be generalised to the wider population. We investigated whether the ERAS for hip replacement was successfully implemented by comparing with a pre-ERAS period, as was done in other studies[32-34], but for the first time, by also comparing with the post-intervention period. We also included all of the hospitals in one country.

Shorter hospital stays were reflected in lower estimated average inpatient bed day costs. Most surgery episodes in the dataset had a hospital stay shorter than the trim point for the cost of the relevant healthcare resource use group. A drop in hospital stay length within the trim point would not be reflected by a change in the estimated average episode costs, if we assign the same unit cost to all patients in the same healthcare resource use group who had a length of stay shorter than the trim point. We therefore estimated the true reduction in NHS expenditure by estimating a cost per bed day reflecting each patient's hospital stay length.

OHS change scores increased across the study period: the difference between pain and function at baseline and 6 months post-surgery was greater at the end of 2016 than in 2008, indicating less pain and better function after surgery. A review of ERAS in total hip replacement showed that better improvement in pain and function scores could be related to



For peer review only - http://bmjopen.bmj.com/site/about/guidelines.xhtml

making patients active participants in their recovery and managing patient expectations[29]. A Cochrane review on preoperative education for hip or knee replacement do not find benefits over usual care, but did find a non-significant reduction in pain and better function associated with perioperative education[38].

Complications 6 months after surgery were decreasing before ERAS was implemented, remained steady during the ERAS period, and increased after the intervention ended. Discharging patients too soon after surgery could increase complications. A meta-analysis of ERAS programmes for colorectal surgery did not find evidence of increased surgical site infections or anastomotic leakage, classified as surgical complications (relative risk = 0.76, 95 % CI 0.54–1.08), and found that cardiovascular, pulmonary, and infectious medical complications decreased (relative risk = 0.40, 95 % CI 0.27–0.61).[39] Patients with diabetes undergoing hip and knee replacement under ERAS protocols had a lower additional risk for complications otherwise associated with operating on patients with diabetes[40].

The 5-year revision surgery rates dropped over the study period, a desirable finding as the revision procedure is more complicated than the initial procedure[41]. Revision rates may have declined due to the UK National Institute for Health and Care Excellence (NICE) recommendation to only use implants with a 10-year revision rate of 5% or lower, to avoid low-quality prostheses[28].

The promising improvements in primary hip replacement outcomes reported here were achieved amidst increasingly strained NHS funding and hospital budgets. NHS funding growth in our study period was slower than historical trends[42]. There are fewer hospital beds available for hip replacement today than at the beginning of the study period, and wards have



For peer review only - http://bmjopen.bmj.com/site/about/guidelines.xhtml

been closed. For example, there was an average daily 10,015 occupied beds open overnight for trauma and orthopaedics in England between April and June 2010, dropping to 8,770 between October and December 2016[43]. Conversely, the number of primary hip replacements in England increased from 67,128 in 2008 to 87,733 in 2016[6]. It has been estimated that 97,516 total hip replacements will take place in 2035[44]. Meeting this demand with existing or lower capacity will require efficiencies. During the period of our study we observed an increasing trend in the proportion of NHS funded primary hip replacements being carried out in independent hospitals (increasing from around 10% in 2008 to 27% in 2016) and a small increase in those within Independent Sector Treatment Centres (ISTC) (from 3.5% in 2008 to 5% in 2016). These changes will have supported an increase in capacity for surgery (although such centres typically treat healthier and less complex patients than nearby public hospitals, with a worsening case-mix of those patients treated in public hospitals[45]. Such changes in the sorting of routine and complex patients between public and private hospital settings over time could also influence observed changes in outcomes of surgery over time. ERAS has kept the improvements happening when other changes were occurring which may have caused deterioration, e.g.: older, sicker, and more obese patients. However, changes in the case-mix of patients have not altered improving trends in outcomes of surgery.

An important issue is the high variation in services and practices across English hospitals. The Getting It Right First Time (GIRFT) programme aims to reduce discrepancies in activity volume, implant choice, and guideline follow-up between hospitals[46]. Despite national improvements in hip replacement outcomes, GIRFT reports substantial variation in outcomes between hospital trusts. In 2016, the mean length of hospital stay after primary hip replacement varied between 2.5 and 11.6 days, and OHS change varied between 12.0 and 23.5 points. Although the national picture has improved for patients, there is still work



For peer review only - http://bmjopen.bmj.com/site/about/guidelines.xhtml

remaining to understand and reduce unwarranted outcome variations between individual hospitals. This study would be strengthened with the comparison with trends from another country (Wales for example). However, we do not have access to hospital admission data for Wales for this study. In addition, the comparison with another procedure where the enhanced recovery intervention was not applied, could also act as a potential control group (such as cataract surgery). Nevertheless, the importance of an external control group would have been higher if within this study we had observed a significant impact of ERAS intervention on a change in trend in outcomes of surgery, as it would have given reassurance of an intervention effect was not observed in the control group. However, in the case of our study, we do not observe an intervention effect – rather than continuation of an existing secular trend that was happening before introduction of the intervention.

Although the NJR registry captures all primary hip replacements including those undertaken in the private sector linkage to English HES data means that we only have access to information on patients receiving NHS funded operations including public and private hospitals. Therefore, this study do not include private funded operations undertaken by the independent sector. It is estimated 13.7% to 19.7% of all hip replacements were carried by the independent sector in 2012-2013 and 2016-2017, respectively (Source: Hospital Episode Statistics, NHS Digital.)

**Conclusion**

Our study shows that outcomes after planned hip replacement are better today than 10 years ago. Hospital stays shortened substantially from 2008 to 2016 for all age groups and in people with and without co-morbidity, without adversely affecting patient outcomes. Patient-reported pain and function have improved, revision rates are in decline, and complication

For peer review only



For peer review only - http://bmjopen.bmj.com/site/about/guidelines.xhtml

rates remain stable. The introduction of a national ERAS programme maintained this improvement but did not alter the rate of change already underway.

**Acknowledgements**

We would like to thank the patients and staff of all the hospitals in England and Wales who have contributed data to the National Joint Registry (NJR); and the Healthcare Quality Improvement Partnership (HQIP), the NJR Steering Committee and staff at the NJR Centre for facilitating this work. The authors have conformed to the NJR's standard protocol for data access and publication. We acknowledge English language editing by Dr Jennifer A de Beyer (Centre for Statistics in Medicine, University of Oxford) and the addition of her informative suggestions. We acknowledge Albert Prats Uribe and Sam Hawley of the Botnar Research Centre (University of Oxford) for providing their expertise in how to conduct an interrupted-time series analysis.

**Contributors**

AJ conceived the study. AJ, JL and CG contributed to study design. AJ acquired raw data from NJR, HES and PROMs registries. CG had full access to all of the study data and takes responsibility for the integrity of the data and the accuracy of the data analysis. JM and JL had full access to all of the study data and takes responsibility for the integrity of the generated health-economic dataset and the accuracy of the health-economic data analysis. CG, JM, JL, NA, AP, DPA, AC, AR, CC, GP, RF, KB and AJ contributed to interpretation of results. CG wrote the first draft of the manuscript and AJ made substantial contributions to the final manuscript. CG produced all tables, figures, supplementary material and appendixes except for Appendix 1, JM wrote Appendix 1. CG, JM, JL, NA, AP, DPA, AC, AR, CC, GP, RF, KB and AJ had full access to all statistical reports and tables in the study. CG, JM, JL,

For peer review only

NA, AP, DPA, AC, AR, CC, GP, RF, KB and AJ contributed to the interpretation of results and critical revision of the manuscript and approved the final manuscript. AJ is the guarantor. The corresponding author (CG) attests that all listed authors meet authorship criteria and that no others meeting the criteria have been omitted.

**Competing interests**

All authors have completed the Unified Competing Interest form at www.icmje.org/coi_disclosure.pdf and declare: AJ has received consultancy fees from Freshfields Bruckhaus Deringer, and has held advisory board positions (which involved receipt of fees) from Anthera Pharmaceuticals, INC. AP reports personal fees from Zimmer Biomet, outside the submitted work. AR reports grants from DePuy Ltd outside the submitted work. CC received personal fees from: Alliance for Better Bone Health; Amgen; Eli Lilly; GSK; Medtronic; Merck; Novartis; Pfizer; Roche; Servier; Takeda; UCB, outside the submitted work. NKA received grants and personal fees outside the submitted work from: Bioberica; Merck; Flexion; Regeneron; Freshfields Bruckhaus Deringer. DPA received grants from: Amgen; UCB Biopharma; Servier; Astellas; Novartis, outside the submitted work. All other authors declare no conflicts of interest.

**Funding**

This project was funded by the NIHR Health Services and Delivery Research programme (project number 14/46/02). Andrew Judge is supported by the NIHR Biomedical Research Centre at the University Hospitals Bristol NHS Foundation Trust and the University of Bristol. The views expressed in this publication are those of the authors and do not necessarily reflect those of the NHS, the National Institute for Health Research or the Department of Health and Social Care.

For peer review only

## Data sharing

Access to data is available from the National Joint Registry for England and Wales, Northern Ireland and the Isle of Man, but restrictions apply to the availability of these data, which were used under license for the current study, and so are not publicly available. Data access applications can be made to the National Joint Registry Research Committee. Access to linked HES and PROMs data is available through data applications to NHS Digital.

## REFERENCES

1. Global, regional, and national incidence, prevalence, and years lived with disability for 328 diseases and injuries for 195 countries, 1990–2016: a systematic analysis for the Global Burden of Disease Study 2016. *The Lancet* 2017;390(10100):1211-59. doi: https://doi.org/10.1016/S0140-6736(17)32154-2

2. Jordan KP, Joud A, Bergknut C, et al. International comparisons of the consultation prevalence of musculoskeletal conditions using population-based healthcare data from England and Sweden. *Annals of the rheumatic diseases* 2014;73(1):212-8. doi: 10.1136/annrheumdis-2012-202634 [published Online First: 2013/01/25]

3. Chen A, Gupte C, Akhtar K, et al. The Global Economic Cost of Osteoarthritis: How the UK Compares. *Arthritis* 2012;2012:698709. doi: 10.1155/2012/698709 [published Online First: 2012/10/20]

4. Hiligsmann M, Cooper C, Arden N, et al. Health economics in the field of osteoarthritis: an expert's consensus paper from the European Society for Clinical and Economic Aspects of Osteoporosis and Osteoarthritis (ESCEO). *Seminars in arthritis and rheumatism* 2013;43(3):303-13. doi: 10.1016/j.semarthrit.2013.07.003 [published Online First: 2013/09/03]

For peer review only

5. Puig-Junoy J, Ruiz Zamora A. Socio-economic costs of osteoarthritis: a systematic review of cost-of-illness studies. *Seminars in arthritis and rheumatism* 2015;44(5):531-41. doi: 10.1016/j.semarthrit.2014.10.012 [published Online First: 2014/12/17]

6. National Joint Registry. 14th Annual Report of the UK NJR. London: National Joint Registry for England, Wales and Northern Ireland, 2017., 2017.

7. NHS_Improving_Quality. Enhanced recovery care pathways: a better journey for patients seven days a week and a better deal for the NHS: NHS England, 2013:16.

8. Ljungqvist O, Hubner M. Enhanced recovery after surgery-ERAS-principles, practice and feasibility in the elderly. *Aging Clin Exp Res* 2018;30(3):249-52. doi: 10.1007/s40520-018-0905-1 [published Online First: 2018/02/18]

9. Campbell M, Fitzpatrick R, Haines A, et al. Framework for design and evaluation of complex interventions to improve health. *BMJ (Clinical research ed)* 2000;321(7262):694-6. [published Online First: 2000/09/15]

10. Craig P, Dieppe P, Macintyre S, et al. Developing and evaluating complex interventions: the new Medical Research Council guidance. *BMJ (Clinical research ed)* 2008;337:a1655. doi: 10.1136/bmj.a1655 [published Online First: 2008/10/01]

11. Wilmore DW, Kehlet H. Management of patients in fast track surgery. *BMJ (Clinical research ed)* 2001;322(7284):473-76. doi: 10.1136/bmj.322.7284.473

12. Paton F, Chambers D, Wilson P, et al. Effectiveness and implementation of enhanced recovery after surgery programmes: a rapid evidence synthesis. *BMJ open* 2014;4(7):e005015. doi: 10.1136/bmjopen-2014-005015 [published Online First: 2014/07/24]

13. NJR-Editorial-Board. 14th Annual Report: National Joint Registry for England, Wales, Northern Ireland and the Isle of Man. http://www.njrreports.org.uk/Portals/0/PDFdownloads/NJR%2014th%20Annual%20Report%202017.pdf 2017:202 pp.

14. Deparment_of_Health. Reference Costs 2015-16, 2016:59.

For peer review only



For peer review only - http://bmjopen.bmj.com/site/about/guidelines.xhtml

15. A Guide to PROMs Methodology. 4 ed: Health and Social Care Information Centre. http://content.digital.nhs.uk/media/1537/A-Guide-to-PROMs-Methodology/pdf/PROMs_Guide_V12.pdf, 2017:42 pp.

16. National-Joint-Registry. Data collection forms - revision procedure for hip. http://www.njrcentre.org.uk/njrcentre/Portals/0/Documents/England/Data%20collection%20forms/MDSv6.0_H2_V004.pdf 2014 [Available from: http://www.njrcentre.org.uk/njrcentre/Healthcareproviders/Collectingdata/Datacollectionforms/tabid/103/Default.aspx accessed 04/12/2017.

17. Deparment_of_Health. Enhanced recovery Partnership Project Report - March 2011. In: NHS-Institute DN-IN, ed.: Department of health, 2011.

18. Charlson ME, Pompei P, Ales KL, et al. A new method of classifying prognostic comorbidity in longitudinal studies: development and validation. *Journal of chronic diseases* 1987;40(5):373-83.

19. Craig P, Cooper C, Gunnell D, et al. Using natural experiments to evaluate population health interventions: new Medical Research Council guidance. *Journal of Epidemiology and Community Health* 2012;66(12):1182-86. doi: 10.1136/jech-2011-200375

20. Kontopantelis E, Doran T, Springate DA, et al. Regression based quasi-experimental approach when randomisation is not an option: interrupted time series analysis. *BMJ (Clinical research ed)* 2015;350:h2750. doi: 10.1136/bmj.h2750 [published Online First: 2015/06/11]

21. Wagner AK, Soumerai SB, Zhang F, et al. Segmented regression analysis of interrupted time series studies in medication use research. *Journal of clinical pharmacy and therapeutics* 2002;27(4):299-309. [published Online First: 2002/08/14]

22. Durbin J. Testing for Serial Correlation in Least-Squares Regression When Some of the Regressors are Lagged Dependent Variables. *Econometrica* 1970;38(3):410-21. doi: 10.2307/1909547

For peer review only

636 23. Newey WK, West KD. A Simple, Positive Semi-Definite, Heteroskedasticity and Autocorrelation Consistent Covariance Matrix. *Econometrica* 1987;55(3):703-08. doi: 10.2307/1913610

639 24. Vandenbroucke JP, Elm E, Altman DG, et al. Strengthening the reporting of observational studies in epidemiology (strobe): Explanation and elaboration. *Annals of internal medicine* 2007;147(8):W-163-W-94. doi: 10.7326/0003-4819-147-8-200710160-00010-w1

643 25. World-Health-Organization. Obesity: preventing and managing the global epidemic. Geneva, Switzerland, 2000:252.

645 26. Lubin MF, Dodson TF, Winawer NH. Medical management of the surgical patient: a textbook of perioperative medicine: Cambridge University Press 2013.

647 27. Burn E, Edwards CJ, Murray DW, et al. Trends and determinants of length of stay and hospital reimbursement following knee and hip replacement: evidence from linked primary care and NHS hospital records from 1997 to 2014. *BMJ open* 2018;8(1):e019146. doi: 10.1136/bmjopen-2017-019146 [published Online First: 2018/01/29]

652 28. NICE-guidelines. Total hip replacement and resurfacing arthroplasty for end-stage arthritis of the hip. Technology appraisal guidance [TA304]. In: Excellence NIfHaC, ed., 2014:60 pp.

655 29. Ibrahim MS, Twaij H, Giebaly DE, et al. Enhanced recovery in total hip replacement. *A clinical review* 2013;95-B(12):1587-94. doi: 10.1302/0301-620x.95b12.31303

657 30. Enhanced-Recovery-Partnership-Programme. Delivering enhanced recovery – Helping patients to get better sooner after surgery. In: Health Do, ed., 2010.

659 31. Tayrose G, Newman D, Slover J, et al. Rapid mobilization decreases length-of-stay in joint replacement patients. *Bulletin of the Hospital for Joint Disease (2013)* 2013;71(3):222-6. [published Online First: 2013/10/25]



For peer review only - http://bmjopen.bmj.com/site/about/guidelines.xhtml

32. Raphael M, Jaeger M, van Vlymen J. Easily adoptable total joint arthroplasty program allows discharge home in two days. *Canadian journal of anaesthesia = Journal canadien d'anesthesie* 2011;58(10):902-10. doi: 10.1007/s12630-011-9565-8 [published Online First: 2011/08/09]

33. Khan SK, Malviya A, Muller SD, et al. Reduced short-term complications and mortality following Enhanced Recovery primary hip and knee arthroplasty: results from 6,000 consecutive procedures. *Acta Orthopaedica* 2014;85(1):26-31. doi: 10.3109/17453674.2013.874925

34. Malviya A, Martin K, Harper I, et al. Enhanced recovery program for hip and knee replacement reduces death rate. *Acta Orthopaedica* 2011;82(5):577-81. doi: 10.3109/17453674.2011.618911

35. Larsen K, Sorensen OG, Hansen TB, et al. Accelerated perioperative care and rehabilitation intervention for hip and knee replacement is effective: a randomized clinical trial involving 87 patients with 3 months of follow-up. *Acta Orthop* 2008;79(2):149-59. doi: 10.1080/17453670710014923 [published Online First: 2008/05/15]

36. Husted H, Lunn TH, Troelsen A, et al. Why still in hospital after fast-track hip and knee arthroplasty? *Acta Orthopaedica* 2011;82(6):679-84. doi: 10.3109/17453674.2011.636682

37. Hebl JR, Dilger JA, Byer DE, et al. A pre-emptive multimodal pathway featuring peripheral nerve block improves perioperative outcomes after major orthopedic surgery. *Regional anesthesia and pain medicine* 2008;33(6):510-7. [published Online First: 2009/03/05]

38. McDonald S, Page MJ, Beringer K, et al. Preoperative education for hip or knee replacement. *Cochrane Database of Systematic Reviews* 2014(5) doi: 10.1002/14651858.CD003526.pub3

39. Greco M, Capretti G, Beretta L, et al. Enhanced Recovery Program in Colorectal Surgery: A Meta-analysis of Randomized Controlled Trials. *World Journal of Surgery* 2014;38(6):1531-41. doi: 10.1007/s00268-013-2416-8

For peer review only



For peer review only - http://bmjopen.bmj.com/site/about/guidelines.xhtml

40. Jorgensen CC, Madsbad S, Kehlet H. Postoperative morbidity and mortality in type-2 diabetics after fast-track primary total hip and knee arthroplasty. *Anesthesia and analgesia* 2015;120(1):230-8. doi: 10.1213/ane.0000000000000451 [published Online First: 2015/01/28]

41. Kandala N-B, Connock M, Pulikottil-Jacob R, et al. Setting benchmark revision rates for total hip replacement: analysis of registry evidence. *BMJ : British Medical Journal* 2015;350 doi: 10.1136/bmj.h756

42. Luchinskaya DS, P. Stoye, G. UK health and social care spending. 2017

43. NHS_England. Bed Availability and Occupancy 2018 [The KH03 is a quarterly collection from all NHS organisations that operate beds, open overnight or day only. It collects the total number of available bed days and the total number of occupied bed days by consultant main specialty. Data for this collection is available back to 2000-01.]. Available from: https://www.england.nhs.uk/statistics/statistical-work-areas/bed-availability-and-occupancy/ accessed 05/04/2018.

44. Culliford D, Maskell J, Judge A, et al. Future projections of total hip and knee arthroplasty in the UK: results from the UK Clinical Practice Research Datalink. *Osteoarthritis and Cartilage* 2015;23(4):594-600. doi: http://dx.doi.org/10.1016/j.joca.2014.12.022

45. Cooper Z, Gibbons S, Skellern M. Does competition from private surgical centres improve public hospitals' performance? Evidence from the English National Health Service. *Journal of Public Economics* 2018;166:63-80. doi: https://doi.org/10.1016/j.jpubeco.2018.08.002

46. Briggs T. A national review of adult elective orthopaedic services in England. GETTING IT RIGHT FIRST TIME: British orthopaedic association, 2015:68.

For peer review only



For peer review only - http://bmjopen.bmj.com/site/about/guidelines.xhtml

## FIGURE LEGENDS

**Fig. 1. Flow diagram showing selection of patients for inclusion in this study (blue shows inclusion and orange shows exclusion).**

**Fig. 2. Effect of the enhanced recovery after surgery (ERAS) programme implemented April 2009 to March 2011 on trends in outcomes following primary hip replacement in England, UK, 2008 – 2016, by month.** (A) Length of hospital stay, (B) change in self-reported pain and function, measured using the Oxford hip score (OHS) at baseline and 6 months after the surgery, (C) any complication in the 6 months after surgery, and (D) hip revision in the 5 years after surgery.

**Fig. 3. Trends in length of hospital stay following primary hip replacement according to patient age categories in England, UK, 2008 – 2016, by month.**

Enhanced recovery after surgery programme implemented in England from April 2009 to March 2011, ERAS.

**Fig. 4. Trends in length of hospital stay following primary hip replacement according to whether patients do or do not present with comorbidities, in England, UK, 2008 – 2016, by month.**

Enhanced recovery after surgery programme implemented in England from April 2009 to March 2011, ERAS.



For peer review only - http://bmjopen.bmj.com/site/about/guidelines.xhtml

**Table 1.** Temporal trends in patients undergoing planned primary hip replacement from April 2008 to December 2016. Parsimonious models with Newey-West standard errors.

| Parameter | Coefficient | Lower 95% CI | Upper 95% CI | *P*-value |
|---|---|---|---|---|
| *Length of stay in hospital* | | | | |
| Intercept | 5.674 | 5.655 | 5.693 | <0.001 |
| Monthly trend | -0.020 | -0.023 | -0.017 | <0.001 |
| Level change $ERAS_0$ | 0.176 | 0.120 | 0.232 | <0.001 |
| Trend change after $ERAS_0$ | -0.013 | -0.017 | -0.009 | <0.001 |
| Level change $ERAS_{end}$ | -0.102 | -0.203 | -0.001 | 0.049 |
| Trend change after $ERAS_{end}$ | 0.019 | 0.015 | 0.022 | <0.001 |
| *Change in Oxford hip score (score at 6 months – score at baseline)* | | | | |
| Intercept | 17.063 | 16.896 | 17.230 | <0.001 |
| Monthly trend | 0.158 | 0.130 | 0.186 | <0.001 |
| Level change $ERAS_0$ | 0.772 | 0.538 | 1.006 | <0.001 |
| Trend change after $ERAS_0$ | -0.131 | -0.161 | -0.101 | <0.001 |
| Level change $ERAS_{end}$ | 0.564 | 0.208 | 0.920 | 0.002 |
| Trend change after $ERAS_{end}$ | -0.013 | -0.025 | -0.001 | 0.039 |
| *Complication by 6 months after surgery* | | | | |
| Intercept | 4.044 | 3.465 | 4.624 | <0.001 |
| Monthly trend | -0.078 | -0.096 | -0.061 | <0.001 |
| Level change $ERAS_0$ | – | – | – | – |
| Trend change after $ERAS_0$ | – | – | – | – |
| Level change $ERAS_{end}$ | – | – | – | – |
| Trend change after $ERAS_{end}$ | 0.078 | 0.056 | 0.100 | <0.001 |



For peer review only - http://bmjopen.bmj.com/site/about/guidelines.xhtml

| | | | | |
|---|---|---|---|---|
| *Revision rates by 5 years after surgery* | | | | |
| Intercept | 7.901 | 7.653 | 8.149 | <0.001 |
| Monthly trend | -0.098 | -0.108 | -0.087 | <0.001 |
| Level change $ERAS_0$ | — | — | — | — |
| Trend change after $ERAS_0$ | — | — | — | — |
| Level change $ERAS_{end}$ | — | — | — | — |
| Trend change after $ERAS_{end}$ | 0.091 | 0.052 | 0.129 | <0.001 |

Confidence intervals, CI; Enhanced Recovery After Surgery, ERAS; start point of ERAS intervention in April 2009, $ERAS_0$; end point of ERAS intervention in March 2011, $ERAS_{end}$; —, $P \geq 0.05$.

For peer review only

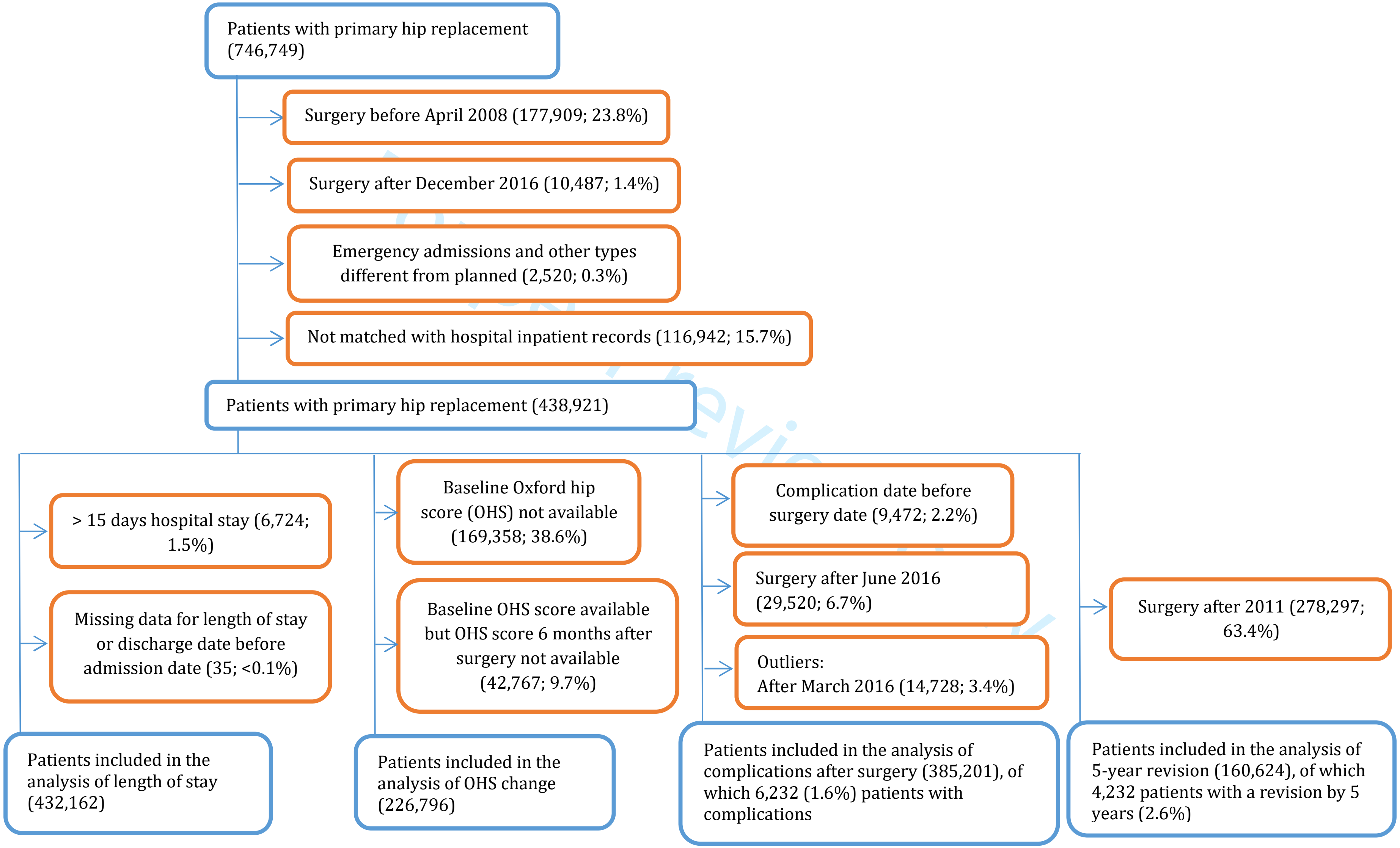
Patients with primary hip replacement (746,749)
Surgery before April 2008 (177,909; 23.8%)
Surgery after December 2016 (10,487; 1.4%)
Emergency admissions and other types different from planned (2,520; 0.3%)
Not matched with hospital inpatient records (116,942; 15.7%)
Patients with primary hip replacement (438,921)
> 15 days hospital stay (6,724; 1.5%)
Missing data for length of stay or discharge date before admission date (35; <0.1%)
Baseline Oxford hip score (OHS) not available (169,358; 38.6%)
Baseline OHS score available but OHS score 6 months after surgery not available (42,767; 9.7%)
Complication date before surgery date (9,472; 2.2%)
Surgery after June 2016 (29,520; 6.7%)
Outliers:
After March 2016 (14,728; 3.4%)
Surgery after 2011 (278,297; 63.4%)
Patients included in the analysis of length of stay (432,162)
Patients included in the analysis of OHS change (226,796)
Patients included in the analysis of complications after surgery (385,201), of which 6,232 (1.6%) patients with complications
Patients included in the analysis of 5-year revision (160,624), of which 4,232 patients with a revision by 5 years (2.6%)

For peer review only

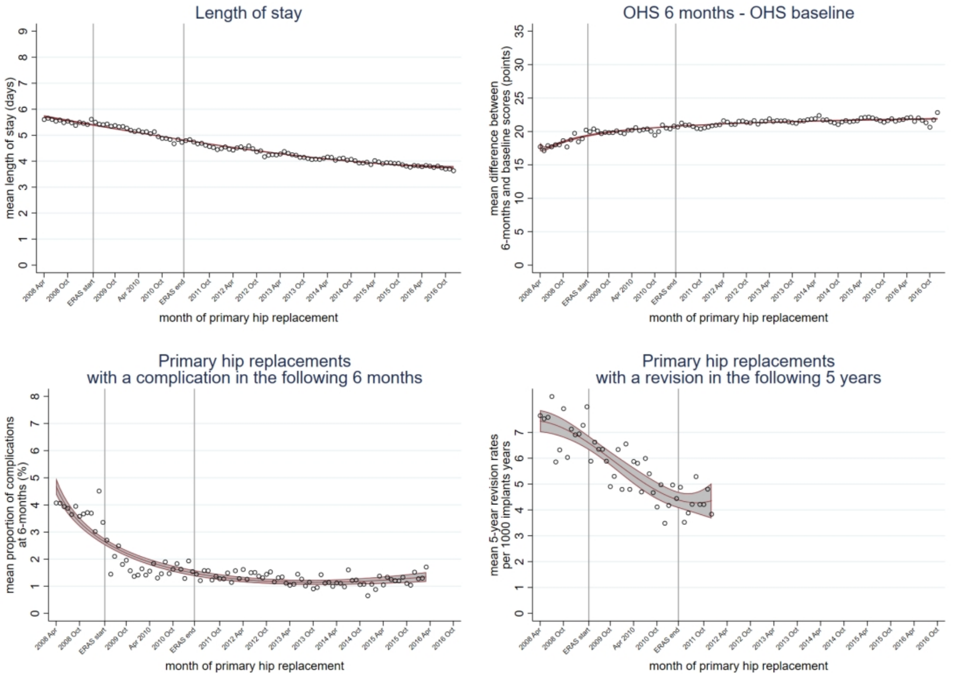


Fig. 2. Effect of the enhanced recovery after surgery (ERAS) programme implemented April 2009 to March 2011 on trends in outcomes following primary hip replacement in England, UK, 2008 – 2016, by month. / (A) Length of hospital stay, (B) change in self-reported pain and function, measured using the Oxford hip score (OHS) at baseline and 6 months after the surgery, (C) any complication in the 6 months after surgery, and (D) hip revision in the 5 years after surgery.

705x513mm (72 x 72 DPI)

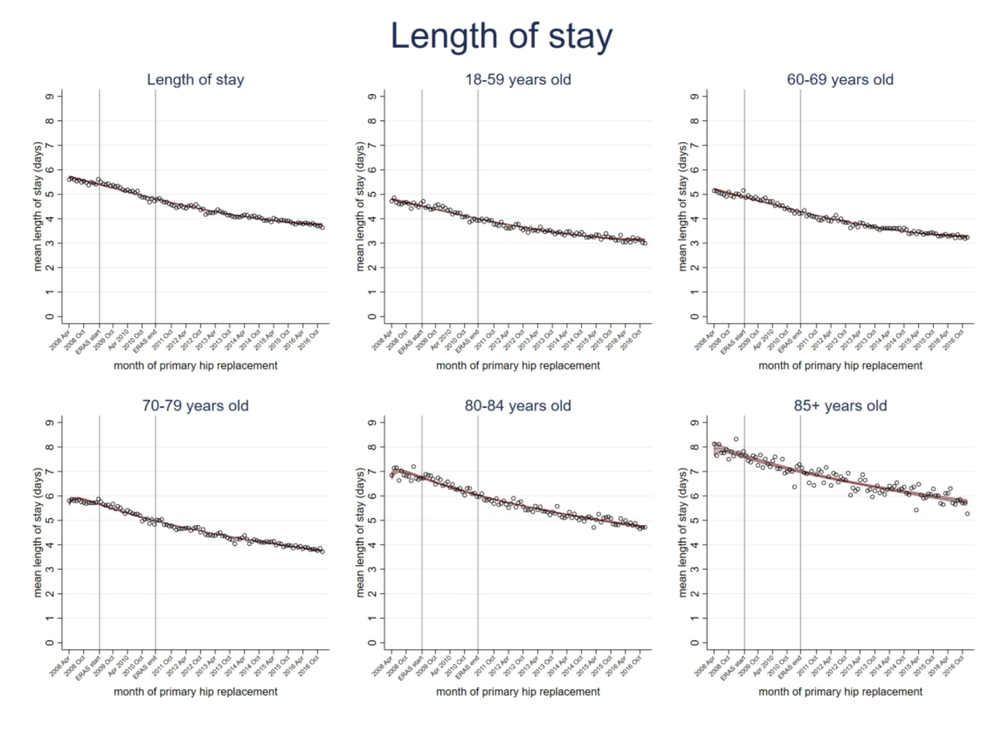


Fig. 3. Trends in length of hospital stay following primary hip replacement according to patient age categories in England, UK, 2008 – 2016, by month. /
Enhanced recovery after surgery programme implemented in England from April 2009 to March 2011, ERAS.

705x513mm (72 x 72 DPI)

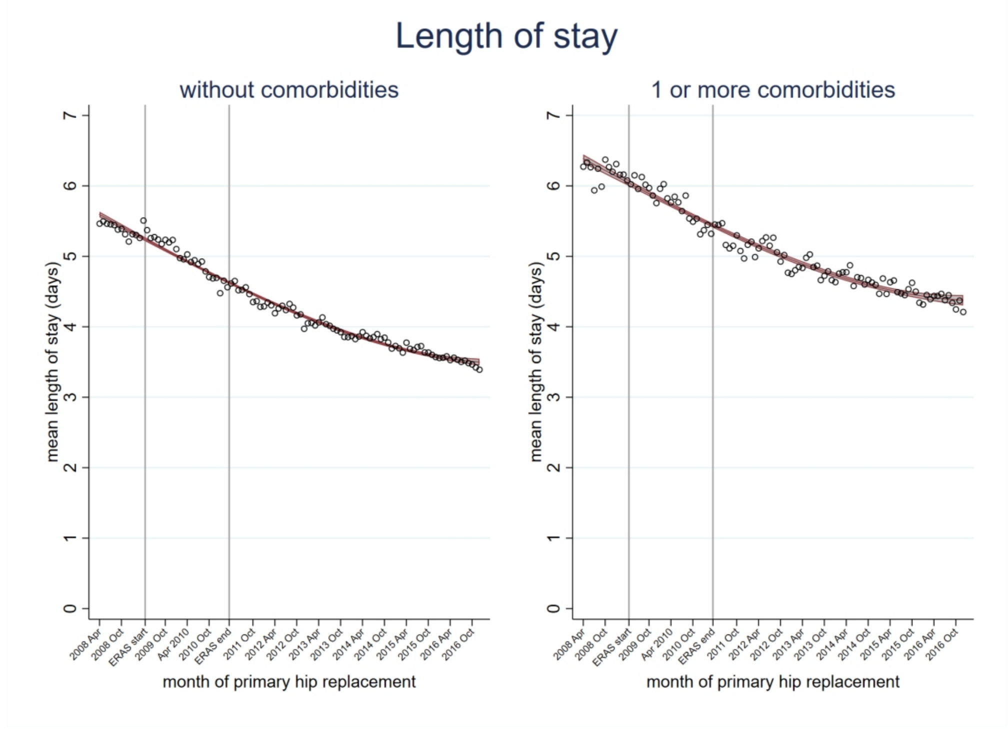


Fig. 4. Trends in length of hospital stay following primary hip replacement according to whether patients do or do not present with comorbidities, in England, UK, 2008 – 2016, by month. /
Enhanced recovery after surgery programme implemented in England from April 2009 to March 2011, ERAS.

705x513mm (72 x 72 DPI)

**MANUSCRIPT TITLE:** Assessment of a National Enhanced Recovery Programme on Patient Outcomes of Primary Hip Replacement: an Interrupted Time Series Analysis from “The National Joint Registry of England and Wales”.

## SUPPLEMENTARY MATERIAL

### Supplementary Text S1. Patient and public involvement

For peer review only

During study development, we identified a person who cares for a wife with extensive arthritis and has himself recently undergone hip replacement. He considered this a worthwhile and important project, but cautioned that enhanced recovery could be perceived as a way for hospitals to discharge patients early to save money.

The study was developed in collaboration with the University of Bristol Musculoskeletal Research Unit’s Patient Experience Partnership in Research (PEP-R) group, comprising patient representatives with experience of both chronic musculoskeletal conditions and joint replacement. They felt that discharge within four days should not become an NHS target, but that discharge time should instead be tailored to individual needs. They identified specific elements of enhanced recovery that they felt could have had a big impact on their recovery.

The James Lind Alliance has identified the need for involving patients in identifying outcomes that matter to them (patient-identified outcomes). We conducted a patient forum with the PEP-R group to identify outcomes from those available in the routine datasets available for this study. The top outcomes were: 1) pain and function, 2) complications (particularly hospital-acquired infection), 3) length of stay (dependent on the level of support at home), 4) revision surgery, and 5) mortality (rated low importance).



For peer review only - http://bmjopen.bmj.com/site/about/guidelines.xhtml

We met with the PEP-R group to report our findings of the effect of the ERAS programme in primary hip replacement on patient outcomes. The group wanted to see graphs of trends in pain, function, and other outcomes, stratified by age group and co-morbidity. They wanted the graphs to be clear, particularly the scale used when outcomes were rare as a restricted scale could be visually misleading. They also wanted the graphs to show the overall trend rather than trend before, during, and after the implementation of ERAS. They found the study finding that reduced length of stay did not adversely affect patient outcomes to be positive.


For peer review only - http://bmjopen.bmj.com/site/about/guidelines.xhtml

**Supplementary Figure S1. Trends in cost per bed day following primary hip replacement in England, UK, 2008 – 2016, by month.**

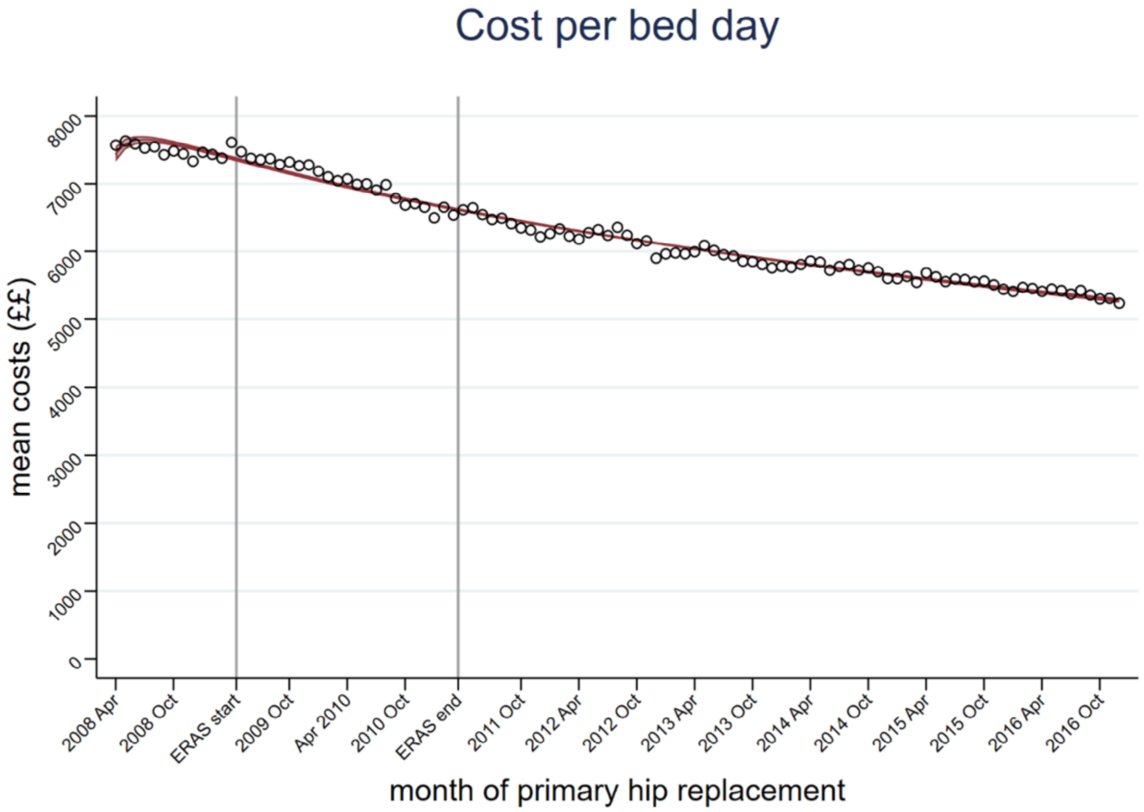


Enhanced recovery after surgery programme implemented in England from April 2009 to March 2011, ERAS.



For peer review only - http://bmjopen.bmj.com/site/about/guidelines.xhtml

**Supplementary Figure S2. Trends in change in Oxford hip score following primary hip replacement according to patient age categories in England, UK, 2008 – 2016, by month.**

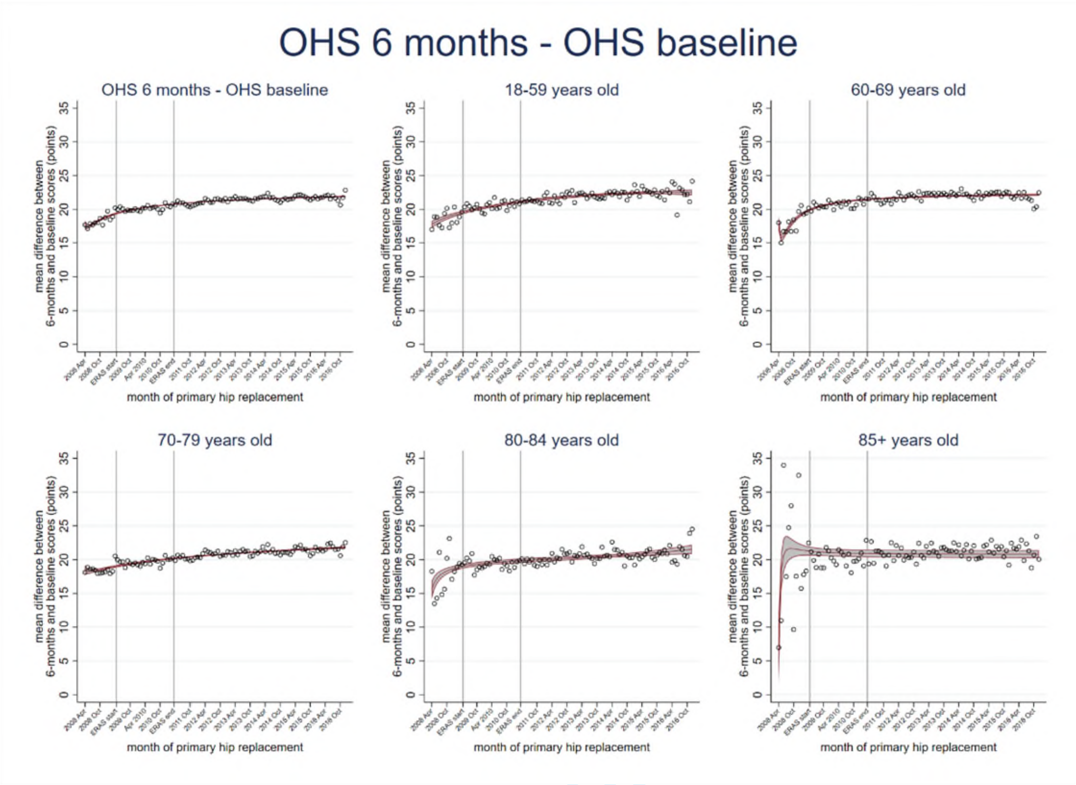


Oxford hip score, OHS; enhanced recovery after surgery programme implemented in England from April 2009 to March 2011, ERAS.



For peer review only - http://bmjopen.bmj.com/site/about/guidelines.xhtml

**Supplementary Figure S3. Trends in change in Oxford hip score following primary hip replacement according to whether patients do or do not present with comorbidities, in England, UK, 2008 – 2016, by month.**

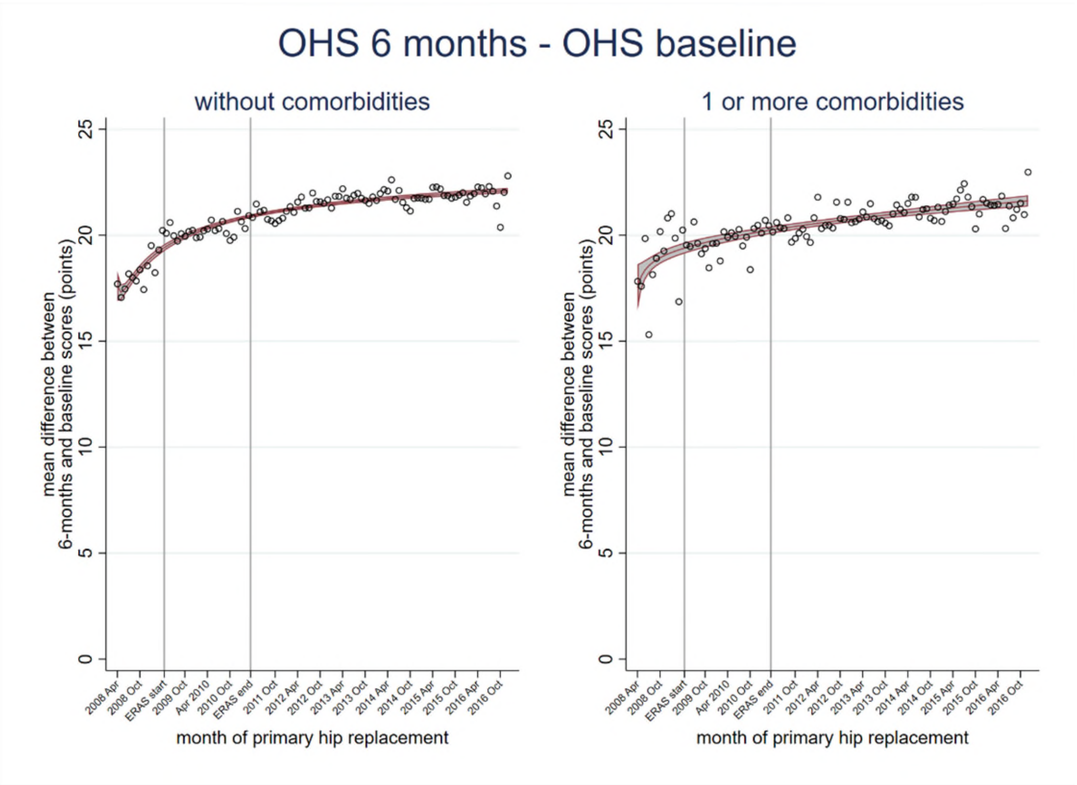


Oxford hip score, OHS; enhanced recovery after surgery programme implemented in England from April 2009 to March 2011, ERAS.



For peer review only - http://bmjopen.bmj.com/site/about/guidelines.xhtml

**Supplementary Table S1.** Temporal trends in patients underwent planned primary hip replacement from April 2008 to December 2016. Full models with Newey-West standard errors.

| **Parameter** | **Coefficient** | **Lower 95% CI** | **Upper 95% CI** | ***P*-value** |
|---|---|---|---|---|
| *Length of stay in hospital* | | | | |
| Intercept | 5.674 | 5.655 | 5.693 | <0.001 |
| Monthly trend | -0.020 | -0.023 | -0.017 | <0.001 |
| Level change $ERAS_0$ | 0.176 | 0.120 | 0.232 | <0.001 |
| Trend change after $ERAS_0$ | -0.013 | -0.017 | -0.009 | <0.001 |
| Level change $ERAS_{end}$ | -0.102 | -0.203 | -0.001 | 0.049 |
| Trend change after $ERAS_{end}$ | 0.019 | 0.015 | 0.022 | <0.001 |
| *Change in Oxford hip score (score at 6 months – score at baseline)* | | | | |
| Intercept | 17.063 | 16.896 | 17.230 | <0.001 |
| Monthly trend | 0.158 | 0.130 | 0.186 | <0.001 |
| Level change $ERAS_0$ | 0.772 | 0.538 | 1.006 | <0.001 |
| Trend change after $ERAS_0$ | -0.131 | -0.161 | -0.101 | <0.001 |
| Level change $ERAS_{end}$ | 0.564 | 0.208 | 0.920 | 0.002 |
| Trend change after $ERAS_{end}$ | -0.013 | -0.025 | -0.001 | 0.039 |
| *Complication rates by 6 months after surgery* | | | | |
| Intercept | 4.254 | 4.161 | 4.347 | <0.001 |
| Monthly trend | -0.072 | -0.088 | -0.056 | <0.001 |
| Level change $ERAS_0$ | -0.729 | -1.531 | 0.072 | 0.074 |
| Trend change after $ERAS_0$ | 0.011 | -0.037 | 0.060 | 0.648 |

For peer review only



For peer review only - http://bmjopen.bmj.com/site/about/guidelines.xhtml

| | | | | |
|---|---|---|---|---|
| Level change $ERAS_{end}$ | 0.179 | -0.372 | 0.730 | 0.521 |
| Trend change after $ERAS_{end}$ | 0.057 | 0.006 | 0.107 | 0.029 |
| *Revision rates by 5 years after surgery* | | | | |
| Intercept | 7.755 | 7.385 | 8.124 | <0.001 |
| Monthly trend | -0.091 | -0.138 | -0.044 | <0.001 |
| Level change $ERAS_0$ | 0.322 | -0.137 | 0.780 | 0.164 |
| Trend change after $ERAS_0$ | -0.023 | -0.083 | 0.036 | 0.432 |
| Level change $ERAS_{end}$ | 0.111 | -0.233 | 0.455 | 0.518 |
| Trend change after $ERAS_{end}$ | 0.112 | 0.066 | 0.157 | <0.001 |

Confidence intervals, CI; Oxford hip score, OHS; Enhanced Recovery After Surgery, ERAS ; start point of ERAS intervention in April 2009, $ERAS_0$; end point of ERAS intervention in March 2011, $ERAS_{end}$.

For peer review only

**Supplementary Table S2.** Temporal trends in patients underwent planned primary hip replacement according to length of stay by age group and comorbidities. April 2008 to December 2016. Parsimonious models with Newey-West standard errors.

| **Parameter** | **Coefficient** | **Lower 95% CI** | **Upper 95% CI** | ***P*-value** |
|---|---|---|---|---|
| *Length of stay in hospital* | | | | |
| *18-59 years old* | | | | |
| Intercept | 4.789 | 4.747 | 4.831 | <0.001 |
| Monthly trend | -0.021 | -0.027 | -0.016 | <0.001 |
| Level change $ERAS_0$ | 0.189 | 0.067 | 0.310 | 0.003 |
| Trend change after $ERAS_0$ | -0.012 | -0.019 | -0.006 | <0.001 |
| Level change $ERAS_{end}$ | — | — | — | — |
| Trend change after $ERAS_{end}$ | 0.021 | 0.014 | 0.027 | <0.001 |
| *60-69 years old* | | | | |
| Intercept | 5.126 | 5.063 | 5.188 | <0.001 |
| Monthly trend | -0.015 | -0.024 | -0.006 | 0.001 |
| Level change $ERAS_0$ | 0.129 | 0.047 | 0.211 | 0.002 |
| Trend change after $ERAS_0$ | -0.017 | -0.025 | -0.009 | <0.001 |
| Level change $ERAS_{end}$ | -0.142 | -0.238 | -0.046 | 0.004 |
| Trend change after $ERAS_{end}$ | 0.018 | 0.015 | 0.021 | <0.001 |
| *70-79 years old* | | | | |
| Intercept | 5.875 | 5.858 | 5.892 | <0.001 |
| Monthly trend | -0.015 | -0.017 | -0.012 | <0.001 |



For peer review only - http://bmjopen.bmj.com/site/about/guidelines.xhtml

| | | | | |
|---|---|---|---|---|
| Level change $ERAS_0$ | 0.189 | 0.133 | 0.244 | <0.001 |
| Trend change after $ERAS_0$ | -0.026 | -0.030 | -0.022 | <0.001 |
| Level change $ERAS_{end}$ | — | — | — | — |
| Trend change after $ERAS_{end}$ | 0.023 | 0.019 | 0.028 | <0.001 |
| *80-84 years old* | | | | |
| Intercept | 6.896 | 6.848 | 6.943 | <0.001 |
| Monthly trend | — | — | — | — |
| Level change $ERAS_0$ | — | — | — | — |
| Trend change after $ERAS_0$ | -0.033 | -0.036 | -0.030 | <0.001 |
| Level change $ERAS_{end}$ | -0.179 | -0.246 | -0.112 | <0.001 |
| Trend change after $ERAS_{end}$ | 0.016 | 0.012 | 0.019 | <0.001 |
| *≥85 years old* | | | | |
| Intercept | 7.855 | 7.801 | 7.908 | <0.001 |
| Monthly trend | — | — | — | — |
| Level change $ERAS_0$ | -0.126 | -0.201 | -0.051 | 0.001 |
| Trend change after $ERAS_0$ | -0.032 | -0.036 | -0.027 | <0.001 |
| Level change $ERAS_{end}$ | — | — | — | — |
| Trend change after $ERAS_{end}$ | 0.014 | 0.008 | 0.020 | <0.001 |
| *Without comorbidities* | | | | |
| Intercept | 5.554 | 5.532 | 5.576 | <0.001 |
| Monthly trend | -0.024 | -0.027 | -0.021 | <0.001 |
| Level change $ERAS_0$ | 0.239 | 0.168 | 0.309 | <0.001 |
| Trend change after $ERAS_0$ | -0.016 | -0.022 | -0.010 | <0.001 |
| Level change $ERAS_{end}$ | — | — | — | — |



For peer review only - http://bmjopen.bmj.com/site/about/guidelines.xhtml

For peer review only

| | | | | |
|---|---|---|---|---|
| Trend change after $ERAS_{end}$ | 0.023 | 0.018 | 0.029 | <0.001 |
| *1+ comorbidities* | | | | |
| Intercept | 6.218 | 6.186 | 6.250 | <0.001 |
| Monthly trend | — | — | — | — |
| Level change $ERAS_0$ | — | — | — | — |
| Trend change after $ERAS_0$ | -0.032 | -0.036 | -0.029 | <0.001 |
| Level change $ERAS_{end}$ | -0.129 | -0.240 | -0.018 | 0.024 |
| Trend change after $ERAS_{end}$ | 0.018 | 0.014 | 0.021 | <0.001 |

Confidence intervals, CI; Enhanced Recovery After Surgery, ERAS ; start point of the ERAS intervention in April 2009, $ERAS_0$; end point of the ERAS intervention in March 2011, $ERAS_{end}$; —, $P \geq 0.05$.

For peer review only



For peer review only - http://bmjopen.bmj.com/site/about/guidelines.xhtml

**Supplementary Table S3.** Temporal trends in patients underwent planned primary hip replacement according to length of stay by age group and comorbidities. April 2008 to December 2016. Full models with Newey-West standard errors.

| **Parameter** | **Coefficient** | **Lower 95% CI** | **Upper 95% CI** | ***P*-value** |
|---|---|---|---|---|
| *Length of stay in hospital* | | | | |
| *18-59 years old* | | | | |
| Intercept | 4.789 | 4.747 | 4.831 | <0.001 |
| Monthly trend | -0.021 | -0.027 | -0.016 | <0.001 |
| Level change $ERAS_0$ | 0.152 | 0.049 | 0.255 | 0.004 |
| Trend change after $ERAS_0$ | -0.008 | -0.015 | -0.001 | 0.028 |
| Level change $ERAS_{end}$ | -0.095 | -0.225 | 0.035 | 0.149 |
| Trend change after $ERAS_{end}$ | 0.017 | 0.011 | 0.022 | <0.001 |
| *60-69 years old* | | | | |
| Intercept | 5.126 | 5.063 | 5.188 | <0.001 |
| Monthly trend | -0.015 | -0.024 | -0.006 | 0.001 |
| Level change $ERAS_0$ | 0.129 | 0.047 | 0.211 | 0.002 |
| Trend change after $ERAS_0$ | -0.017 | -0.025 | -0.009 | <0.001 |
| Level change $ERAS_{end}$ | -0.142 | -0.238 | -0.046 | 0.004 |
| Trend change after $ERAS_{end}$ | 0.018 | 0.015 | 0.021 | <0.001 |
| *70-79 years old* | | | | |
| Intercept | 5.875 | 5.858 | 5.892 | <0.001 |
| Monthly trend | -0.015 | -0.017 | -0.012 | <0.001 |
| Level change $ERAS_0$ | 0.159 | 0.125 | 0.193 | <0.001 |



For peer review only - http://bmjopen.bmj.com/site/about/guidelines.xhtml

| | | | | |
|---|---|---|---|---|
| Trend change after $ERAS_0$ | -0.022 | -0.026 | -0.019 | <0.001 |
| Level change $ERAS_{end}$ | -0.076 | -0.161 | 0.008 | 0.077 |
| Trend change after $ERAS_{end}$ | 0.020 | 0.018 | 0.023 | <0.001 |
| *80-84 years old* | | | | |
| Intercept | 7.016 | 6.931 | 7.102 | <0.001 |
| Monthly trend | -0.017 | -0.029 | -0.005 | 0.005 |
| Level change $ERAS_0$ | 0.079 | -0.019 | 0.177 | 0.113 |
| Trend change after $ERAS_0$ | -0.016 | -0.028 | -0.003 | 0.015 |
| Level change $ERAS_{end}$ | -0.181 | -0.250 | -0.112 | <0.001 |
| Trend change after $ERAS_{end}$ | 0.015 | 0.011 | 0.020 | <0.001 |
| *≥85 years old* | | | | |
| Intercept | 7.902 | 7.743 | 8.062 | <0.001 |
| Monthly trend | -0.007 | -0.030 | 0.016 | 0.562 |
| Level change $ERAS_0$ | -0.070 | -0.240 | 0.100 | 0.415 |
| Trend change after $ERAS_0$ | -0.027 | -0.050 | -0.004 | 0.020 |
| Level change $ERAS_{end}$ | 0.056 | -0.083 | 0.195 | 0.425 |
| Trend change after $ERAS_{end}$ | 0.016 | 0.010 | 0.022 | <0.001 |
| *Without comorbidities* | | | | |
| Intercept | 5.554 | 5.532 | 5.576 | <0.001 |
| Monthly trend | -0.024 | -0.027 | -0.021 | <0.001 |
| Level change $ERAS_0$ | 0.201 | 0.155 | 0.247 | <0.001 |
| Trend change after $ERAS_0$ | -0.012 | -0.017 | -0.007 | <0.001 |
| Level change $ERAS_{end}$ | -0.096 | -0.202 | 0.009 | 0.074 |
| Trend change after $ERAS_{end}$ | 0.020 | 0.017 | 0.022 | <0.001 |



For peer review only - http://bmjopen.bmj.com/site/about/guidelines.xhtml

*1+ comorbidities*

| | | | | |
|---|---|---|---|---|
| Intercept | 6.220 | 6.134 | 6.305 | <0.001 |
| Monthly trend | -0.001 | -0.011 | 0.010 | 0.902 |
| Level change $ERAS_0$ | 0.012 | -0.088 | 0.112 | 0.813 |
| Trend change after $ERAS_0$ | -0.032 | -0.042 | -0.022 | <0.001 |
| Level change $ERAS_{end}$ | -0.126 | -0.245 | -0.007 | 0.038 |
| Trend change after $ERAS_{end}$ | 0.018 | 0.013 | 0.023 | <0.001 |

Confidence intervals, CI; Enhanced Recovery After Surgery, ERAS ; start point of ERAS intervention in April 2009, $ERAS_0$; end point of ERAS intervention in March 2011, $ERAS_{end}$.

For peer review only



For peer review only - http://bmjopen.bmj.com/site/about/guidelines.xhtml

**Supplementary Table S4.** Distribution of primary hip replacements and percentage of missing values per potential modifier of OHS. England (April 2008 to December 2016)

| | **n** | **%** | **% missing values** | | |
|---|---|---|---|---|---|
| | | | **OHS at baseline** | **OHS at 6 months** | **OHS change** |
| *Total* | 438,921 | 100 | 38.6 | 47.9 | 48.3 |
| *Period* | | | *P*<0.001 | *P*<0.001 | *P*<0.001 |
| Before ERAS (April 2008 - March 2009) | 38,362 | 8.7 | 87.4 | 89.6 | 89.7 |
| During ERAS (April 2009 - March 2011) | 86,416 | 19.7 | 33.2 | 41.6 | 41.9 |
| After ERAS (April 2011 - December 2016) | 314,143 | 71.6 | 34.1 | 44.6 | 45.0 |
| *Age at surgery (years)* | | | *P*<0.001 | *P*<0.001 | *P*<0.001 |
| 18-59 | 78,383 | 17.9 | 36.9 | 52.7 | 52.9 |
| 60-69 | 13 1,441 | 30.0 | 36.1 | 44.4 | 44.7 |
| 70-79 | 157.241 | 35.8 | 39.0 | 46.2 | 46.7 |
| 80-84 | 47,919 | 10.9 | 42.5 | 50.8 | 51.3 |
| 85 or more | 23,937 | 5.5 | 47.3 | 56.9 | 57.5 |
| *Comorbidities* | | | *P*<0.001 | *P*<0.001 | *P*<0.001 |
| None | 325,750 | 74.2 | 38.3 | 47.1 | 47.5 |
| 1 or more | 113,171 | 25.8 | 39.5 | 50.3 | 50.7 |

Oxford hip score, OHS; Enhanced Recovery After Surgery, ERAS . *P* values are calculated from 2-sided Pearson's statistic. OHS change is the difference between OHS at 6 months and OHS at baseline.



For peer review only - http://bmjopen.bmj.com/site/about/guidelines.xhtml

**Supplementary Table S5.** Distribution of potential modifiers for primary hip replacements with complete and incomplete data for change in OHS (6 month - baseline scores). England (April 2008 to December 2016)

| | **Incomplete cases** | | **Complete cases** | | ***P* value** |
|---|---|---|---|---|---|
| | **n** | **%** | **n** | **%** | |
| *Period* | | | | | *P*<0.001 |
| Before ERAS (April 2008 - March 2009) | 34,405 | 16.2 | 3,957 | 1.7 | |
| During ERAS (April 2009 - March 2011) | 36,242 | 17.1 | 50,174 | 22.1 | |
| After ERAS (April 2011 - December 2016) | 141,478 | 66.7 | 172,665 | 76.1 | |
| *Age at surgery (years)* | | | | | *P*<0.001 |
| 18-59 | 41,497 | 19.6 | 36,886 | 16.3 | |
| 60-69 | 58,812 | 27.7 | 72,629 | 32.0 | |
| 70-79 | 73,480 | 34.6 | 83,761 | 36.9 | |
| 80-84 | 24,584 | 11.6 | 23,335 | 10.3 | |
| 85 or more | 13,752 | 6.5 | 10,185 | 4.5 | |
| *Comorbidities* | | | | | *P*<0.001 |
| None | 154,718 | 72.9 | 171032.0 | 75.4 | |
| 1 or more | 57,407 | 27.1 | 55764.0 | 24.6 | |



For peer review only - http://bmjopen.bmj.com/site/about/guidelines.xhtml

Oxford hip score, OHS; Enhanced Recovery After Surgery, ERAS . *P* values are calculated from 2-sided Pearson's statistic. Incomplete cases defined by a missing value in OHS at baseline or OHS at 6 months.

For peer review only

**Supplementary Table S6.** Temporal trends in patients underwent planned primary hip replacement according to OHS change by age group and comorbidities. April 2008 to December 2016. Parsimonious models with Newey-West standard errors.

| **Parameter** | **Coefficient** | **Lower 95% CI** | **Upper 95% CI** | ***P*-value** |
|---|---|---|---|---|
| *Change in Oxford hip score (score at 6 months – score at baseline)* | | | | |
| *18-59 years old* | | | | |
| Intercept | 18.096 | 17.853 | 18.340 | <0.001 |
| Monthly trend | 0.052 | 0.034 | 0.070 | <0.001 |
| Level change $ERAS_0$ | 1.039 | 0.617 | 1.461 | <0.001 |
| Trend change after $ERAS_0$ | — | — | — | — |
| Level change $ERAS_{end}$ | 0.334 | 0.014 | 0.655 | 0.041 |
| Trend change after $ERAS_{end}$ | -0.031 | -0.052 | -0.011 | 0.003 |
| *60-69 years old* | | | | |
| Intercept | 15.185 | 14.473 | 15.897 | <0.001 |
| Monthly trend | 0.395 | 0.320 | 0.471 | <0.001 |
| Level change $ERAS_0$ | | | | |
| Trend change after $ERAS_0$ | -0.344 | -0.433 | -0.254 | <0.001 |
| Level change $ERAS_{end}$ | 0.639 | 0.076 | 1.203 | 0.027 |
| Trend change after $ERAS_{end}$ | -0.048 | -0.074 | -0.021 | 0.001 |
| *70-79 years old* | | | | |
| Intercept | 18.645 | 18.474 | 18.816 | <0.001 |
| Monthly trend | -0.047 | -0.068 | -0.026 | <0.001 |



For peer review only - http://bmjopen.bmj.com/site/about/guidelines.xhtml

| | | | | |
|---|---|---|---|---|
| Level change $ERAS_0$ | 1.269 | 1.109 | 1.429 | <0.001 |
| Trend change after $ERAS_0$ | 0.068 | 0.046 | 0.089 | <0.001 |
| Level change $ERAS_{end}$ | 0.494 | 0.158 | 0.829 | 0.004 |
| Trend change after $ERAS_{end}$ | — | — | — | — |
| *80-84 years old* | | | | |
| Intercept | 15.235 | 14.237 | 16.232 | <0.001 |
| Monthly trend | 0.358 | 0.262 | 0.454 | <0.001 |
| Level change $ERAS_0$ | — | — | — | — |
| Trend change after $ERAS_0$ | -0.370 | -0.480 | -0.260 | <0.001 |
| Level change $ERAS_{end}$ | 0.548 | 0.028 | 1.068 | 0.039 |
| Trend change after $ERAS_{end}$ | 0.034 | 0.007 | 0.061 | 0.016 |
| *≥85 years old* | | | | |
| Intercept | 19.946 | 19.233 | 20.658 | <0.001 |
| Monthly trend | — | — | — | — |
| Level change $ERAS_0$ | — | — | — | — |
| Trend change after $ERAS_0$ | — | — | — | — |
| Level change $ERAS_{end}$ | 1.249 | 0.504 | 1.993 | 0.001 |
| Trend change after $ERAS_{end}$ | — | — | — | — |
| *Without comorbidities* | | | | |
| Intercept | 17.106 | 16.939 | 17.273 | <0.001 |
| Monthly trend | 0.133 | 0.105 | 0.160 | <0.001 |
| Level change $ERAS_0$ | 1.306 | 1.088 | 1.525 | <0.001 |
| Trend change after $ERAS_0$ | -0.118 | -0.147 | -0.090 | <0.001 |
| Level change $ERAS_{end}$ | 0.795 | 0.352 | 1.237 | 0.001 |


For peer review only - http://bmjopen.bmj.com/site/about/guidelines.xhtml

| | | | | |
|---|---|---|---|---|
| Trend change after $ERAS_{end}$ | — | — | — | — |
| *1+ comorbidities* | | | | |
| Intercept | 16.642 | 16.003 | 17.282 | <0.001 |
| Monthly trend | 0.334 | 0.260 | 0.408 | <0.001 |
| Level change $ERAS_0$ | -1.677 | -2.132 | -1.223 | <0.001 |
| Trend change after $ERAS_0$ | -0.279 | -0.357 | -0.202 | <0.001 |
| Level change $ERAS_{end}$ | — | — | — | — |
| Trend change after $ERAS_{end}$ | -0.034 | -0.056 | -0.013 | 0.002 |

Oxford hip score, OHS; confidence intervals, CI; Enhanced Recovery After Surgery, ERAS ; start point of ERAS intervention in April 2009, $ERAS_0$; end point of ERAS intervention in March 2011, $ERAS_{end}$; —, $P \geq 0.05$.

For peer review only

**Supplementary Table S7.** Temporal trends in patients underwent planned primary hip replacement according to OHS change by age group and comorbidities. April 2008 to December 2016. Full models with Newey-West standard errors.

| **Parameter** | **Coefficient** | **Lower 95% CI** | **Upper 95% CI** | ***P*-value** |
|---|---|---|---|---|
| *Change in Oxford hip score (score at 6 months – score at baseline)* | | | | |
| *18-59 years old* | | | | |
| Intercept | 17.781 | 17.368 | 18.193 | <0.001 |
| Monthly trend | 0.097 | 0.040 | 0.154 | 0.001 |
| Level change $ERAS_0$ | 0.867 | 0.439 | 1.295 | <0.001 |
| Trend change after $ERAS_0$ | -0.049 | -0.110 | 0.011 | 0.110 |
| Level change $ERAS_{end}$ | 0.384 | 0.070 | 0.698 | 0.017 |
| Trend change after $ERAS_{end}$ | -0.027 | -0.045 | -0.010 | 0.003 |
| *60-69 years old* | | | | |
| Intercept | 15.451 | 14.644 | 16.257 | <0.001 |
| Monthly trend | 0.342 | 0.235 | 0.450 | <0.001 |
| Level change $ERAS_0$ | 0.578 | -0.102 | 1.259 | 0.095 |
| Trend change after $ERAS_0$ | -0.303 | -0.406 | -0.200 | <0.001 |
| Level change $ERAS_{end}$ | 0.737 | 0.173 | 1.300 | 0.011 |
| Trend change after $ERAS_{end}$ | -0.035 | -0.057 | -0.013 | 0.002 |
| *70-79 years old* | | | | |
| Intercept | 18.645 | 18.473 | 18.817 | <0.001 |
| Monthly trend | -0.047 | -0.068 | -0.026 | <0.001 |
| Level change $ERAS_0$ | 1.264 | 0.977 | 1.552 | <0.001 |

For peer review only

| | | | | |
|---|---|---|---|---|
| Trend change after $ERAS_0$ | 0.068 | 0.040 | 0.096 | <0.001 |
| Level change $ERAS_{end}$ | 0.490 | 0.106 | 0.874 | 0.013 |
| Trend change after $ERAS_{end}$ | 0.000 | -0.018 | 0.017 | 0.967 |
| *80-84 years old* | | | | |
| Intercept | 15.207 | 14.066 | 16.349 | <0.001 |
| Monthly trend | 0.364 | 0.217 | 0.511 | <0.001 |
| Level change $ERAS_0$ | -0.060 | -0.965 | 0.844 | 0.895 |
| Trend change after $ERAS_0$ | -0.374 | -0.522 | -0.226 | <0.001 |
| Level change $ERAS_{end}$ | 0.537 | 0.015 | 1.060 | 0.044 |
| Trend change after $ERAS_{end}$ | 0.033 | 0.008 | 0.057 | 0.011 |
| *≥85 years old* | | | | |
| Intercept | 16.187 | 9.205 | 23.168 | <0.001 |
| Monthly trend | 0.487 | -0.395 | 1.368 | 0.276 |
| Level change $ERAS_0$ | -1.708 | -5.891 | 2.474 | 0.420 |
| Trend change after $ERAS_0$ | -0.503 | -1.386 | 0.380 | 0.261 |
| Level change $ERAS_{end}$ | 1.014 | 0.495 | 1.532 | <0.001 |
| Trend change after $ERAS_{end}$ | 0.024 | -0.005 | 0.053 | 0.099 |
| *Without comorbidities* | | | | |
| Intercept | 17.106 | 16.938 | 17.274 | <0.001 |
| Monthly trend | 0.133 | 0.105 | 0.160 | <0.001 |
| Level change $ERAS_0$ | 1.187 | 0.967 | 1.406 | <0.001 |
| Trend change after $ERAS_0$ | -0.109 | -0.137 | -0.080 | <0.001 |
| Level change $ERAS_{end}$ | 0.698 | 0.313 | 1.083 | 0.001 |
| Trend change after $ERAS_{end}$ | -0.010 | -0.021 | 0.001 | 0.083 |
| *1+ comorbidities* | | | | |



For peer review only - http://bmjopen.bmj.com/site/about/guidelines.xhtml

| | | | | |
|---|---|---|---|---|
| Intercept | 16.642 | 16.000 | 17.285 | <0.001 |
| Monthly trend | 0.334 | 0.260 | 0.408 | <0.001 |
| Level change $ERAS_0$ | -1.640 | -2.136 | -1.144 | <0.001 |
| Trend change after $ERAS_0$ | -0.284 | -0.362 | -0.206 | <0.001 |
| Level change $ERAS_{end}$ | 0.097 | -0.279 | 0.472 | 0.610 |
| Trend change after $ERAS_{end}$ | -0.030 | -0.054 | -0.006 | 0.013 |

Oxford hip score, OHS; confidence intervals, CI; Enhanced Recovery After Surgery, ERAS; start point of ERAS intervention in April 2009, $ERAS_0$; end point of ERAS intervention in March 2011, $ERAS_{end}$.

For peer review only



For peer review only - http://bmjopen.bmj.com/site/about/guidelines.xhtml

**MANUSCRIPT TITLE:**

Impact of a National Enhanced Recovery Programme on Patient Outcomes of Primary Hip Replacement: an Interrupted Time Series Analysis from England.

## APPENDIXES

**Appendix 1.** Cost methods.

Objective

We aimed to estimate the trend in National Health Service (NHS) expenditure over time, reflecting the change in length of stay (LOS) observed.

Grouper and reference cost methods

Using Hospital Episode Statistics (HES) data for the same group of patients as for LOS (i.e. excluding those with length of stay above 15 days), we generated healthcare resource use group (HRG) classifications for the index episode for each patient using the 2015/16 NHS reference costs grouper [1], which were subsequently used to estimate inpatient costs per patient using NHS reference costs from 2015/16 [2].

86% of the index episodes in the data had a LOS below the trim point for the relevant HRG. For example, the most common HRG in the data was hip replacement without complications (HN12F: "Very Major Hip Procedures for Non-Trauma with CC Score 0-1" [2]), constituting 63% of the index episodes in the data. This HRG has a trim point of 6 days, meaning that all episodes with a duration less than 6 days are assigned the same unit cost. A reduction in LOS within the trim point is therefore not reflected in the cost of the episode, despite there being a true reduction in NHS costs. In order to estimate the mean change in NHS expenditure we therefore estimated an adjusted average bed day cost.



For peer review only - http://bmjopen.bmj.com/site/about/guidelines.xhtml

Estimating the adjusted average bed day cost

For each HRG we estimated the average cost per bed day (defined as any part of a day spent in hospital) by dividing the total cost of the index episodes for that HRG by the total number of bed days for that HRG. This generated a single average bed day cost per HRG.

For each patient we estimated the adjusted episode cost by multiplying their length of stay (bed days) by the average bed day cost for the HRG that they had been assigned by the NHS reference costs grouper [1]. Therefore, instead of assigning the same unit cost to all patients with the same HRG who had a LOS below the trim point, the adjusted cost differed according to a patient's LOS, even if that LOS was below the trim point for the HRG. Using this method we were able to estimate the average difference in true NHS expenditure as a result of the reduction in length of stay over time even when the LOS was below the trim point.

The 2015/16 grouper and reference costs [1,2] were used to estimate costs for all patients in all years, as there are differences in the methodologies used for HRG classification in different cost years [3]. This prevents a like-for-like comparison between years if different groupers and/or costs are used.

Costs were estimated for a total of 432,143 patients.

References for Appendix 1

1. HRG4+ 2015/16 Reference Costs Grouper. Copyright © 2015 Health and Social Care Information Centre. Grouper version: RC 15/16. Implementation version: 1516.RC.8
2. Department of Health. NHS reference costs 2015 to 2016. https://www.gov.uk/government/publications/nhs-reference-costs-2015-to-2016
3. Reference Costs 2015-16. Department of Health. https://assets.publishing.service.gov.uk/government/uploads/system/uploads/attachment_data/file/577083/Reference_Costs_2015-16.pdf.



For peer review only - http://bmjopen.bmj.com/site/about/guidelines.xhtml

**Appendix 2.** Operative procedure codes (OPCS 4.8) that we used to identify hip revision in the Hospital Episode Statistics (HES) registry.

Procedure type 1: : W39.4, “Attention to total prosthetic replacement of hip joint (Not Elsewhere Classified, NEC)”; W39.6, “Closed reduction of dislocated total prosthetic replacement of hip joint”; W78.2, “Release of contracture of hip joint”; W95.4, “Attention to hybrid prosthetic replacement of hip joint using cement NEC”; X09.1, “Hindquarter amputation”;  X09.2, “Disarticulation of hip”; X09.3, “Amputation of leg above knee”.

Procedure type 2: W07.2, “Excision of periarticular ectopic bone”; W18.1, “Fenestration of cortex of bone”; W18.2, “Saucerisation of bone”; W18.3, “Sequestrectomy of bone”; W18.4, “Decompression of fourage of bone”; W18.5, “Insertion of drainage system into bone”; W33.6, “Debridement of bone NEC”; W33.7, “Lavage of bone”; W33.8, “Other specified other open operations on bone”; W33.9, “Unspecified other open operations on bone”; W45.4, “Attention to total prosthetic replacement of joint NEC”; W54.4, “Attention to prosthetic interposition arthroplasty of joint NEC”; W60.3, “Conversion to arthrodesis and extra-articular bone graft NEC”; W60.8, “Other specified fusion of other joint and extra-articular bone graft”; W60.9, “Unspecified fusion of other joint and extra-articular bone graft”; W61.3, “Conversion to arthrodesis and articular bone graft NEC”; W61.8, “Other specified fusion of other joint and other articular bone graft”; W61.9, “Unspecified fusion of other joint and other articular bone graft”; W63.1, “Revision of arthrodesis and internal fixation NEC”; W63.2, “Revision of arthrodesis and external fixation NEC”; W63.8, “Other specified revisional fusion of other joint”; W63.9, “Unspecified revisional fusion of other joint”; W64.0, “Conversion from previous arthrodesis NEC”; W64.1, “Conversion to arthrodesis and internal fixation NEC”; W64.2, “Conversion to arthrodesis and external fixation NEC”; W64.8, “Other specified conversion to fusion of other joint”; W64.9, “Unspecified conversion to fusion of other joint”; W69.1, “Total synovectomy”; W69.2,



For peer review only - http://bmjopen.bmj.com/site/about/guidelines.xhtml

“Subtotal synovectomy”; W69.3, “Partial synovectomy”; W69.4, “Open biopsy of synovial membrane of joint”; W69.5, “Open division of synovial plica”; W69.8, “Other specified open operations on synovial membrane of joint”; W69.9, “Unspecified open operations on synovial membrane of joint”; W71.2, “Open excision of intra-articular osteophyte”; W71.3, “Forage of joint”; W71.8, “Other specified other open operations on intra-articular structure”; W71.9, “Unspecified other open operations on intra-articular structure”; W77.1, “Repair of capsule of joint for stabilisation of joint NEC”; W77.2, “Transposition of muscle for stabilisation of joint”; W77.3, “Blocking operations on joint using prosthesis for stabilisation of joint”; W77.4, “Blocking operations on joint using bone for stabilisation of joint”; W77.5, “Periarticular osteotomy for stabilisation of joint”; W77.8, “Other specified stabilising operations on joint”; W77.9, “Unspecified stabilising operations on joint”; W78.4, “Limited release of contracture of capsule of joint”; W78.8, “Other specified release of contracture of joint”; W78.9, “Unspecified release of contracture of joint”; W80.1, “Open debridement and irrigation of joint”; W80.2, “Open debridement of joint NEC”; W80.3, “Open irrigation of joint NEC”; W80.8, “Other specified debridement and irrigation of joint”; W80.9, “Unspecified debridement and irrigation of joint”; W81.1, “Excision of lesion of joint NEC”; W81.2, “Open removal of loose body from joint”; W81.3, “Drainage of joint”; W81.4, “Incision of joint NEC”; W81.5, “Exploration of joint NEC”; W81.6, “Capsulorrhaphy of joint”; W81.7, “Insertion of therapeutic spacer into joint”; W81.8, “Other specified other open operations on joint”; W81.9, “Unspecified other open operations on joint”; W84.3, “Endoscopic division of synovial plica”; W84.4, “Endoscopic decompression of joint”; W84.6, “Endoscopic excision of synovial plica”; W84.8, “Other specified therapeutic endoscopic operations on other joint structure”; W84.9, “Unspecified therapeutic endoscopic operations on other joint structure”; W86.1, “Endoscopic removal of loose body from joint NEC”; W86.8, “Other specified therapeutic endoscopic operations on cavity of other joint”;

For peer review only

W86.9, “Unspecified therapeutic endoscopic operations on cavity of other joint”; W88.1, “Diagnostic endoscopic examination of joint and biopsy of lesion of joint NEC”; W88.8, “Other specified diagnostic endoscopic examination of other joint”; W88.9, “Unspecified diagnostic endoscopic examination of other joint”; W90.1, “Aspiration of joint”; W90.2, “Arthrography”; W90.3, “Injection of therapeutic substance into joint”; W90.4, “Injection into joint NEC”; W90.8, “Other specified puncture of joint”; W90.9, “Unspecified puncture of joint”; W91.1, “Manipulation of joint using traction NEC”; W91.3, “Manipulation of prosthetic joint NEC”; W91.8, “Other specified other manipulation of joint”; W91.9, “Unspecified other manipulation of joint”; W92.1, “Biopsy of lesion of joint NEC”; W92.2, “Distension of joint”; W92.3, “Examination of joint under image intensifier”; W92.4, “Examination of joint under anaesthetic”; W92.5, “Examination of joint NEC”; W92.8, “Other specified other operations on joint”; W92.9, “Unspecified other operations on joint”; O19.8, “Other specified other therapeutic endoscopic operations on other joint structure”; O27.1, “Extra-articular ligament reconstruction for stabilisation of joint”; O27.8, “Other specified other stabilising operations on joint”; O27.9, “Unspecified other stabilising operations on joint”; S57.1, “Debridement of skin NEC”; S57.2, “Removal of slough from skin NEC”; S57.3, “Toilet of skin NEC”; S57.4, “Dressing of skin NEC”; S57.5, “Attention to dressing of skin NEC”; S57.6, “Cleansing and sterilisation of skin NEC”; S57.7, “Dressing of skin using vacuum assisted closure device NEC”; S57.8, “Other specified exploration of other skin of other site”; S57.9, “Unspecified exploration of other skin of other site”; S58.2, “Larvae debridement therapy of skin NEC”; X09.8, “Other specified amputation of leg”; X09.9, “Unspecified amputation of leg”.

Site for revision: Z84.3, “Hip joint”; Z76.1, “Head of femur”; Z75.6, “Acetabulum”; Z84.9, “Joint of pelvis or upper leg NEC”.



For peer review only - http://bmjopen.bmj.com/site/about/guidelines.xhtml

Algorithm: One code from procedure type 1 or a combination of one code from procedure type 2 and site for revision were used to identify hip revision.

For peer review only



For peer review only - http://bmjopen.bmj.com/site/about/guidelines.xhtml

**Appendix 3.** Codes defined in the International Statistical Classification of Diseases and Related Health Problems 10th Revision (ICD-10) that we used to identify complications in the Hospital Episode Statistics (HES) registry.

<u>Stroke</u>: I60.X, "Subarachnoid haemorrhage"; I61.0, "Intracerebral haemorrhage in hemisphere, subcortical"; I61.1, "Intracerebral haemorrhage in hemisphere, cortical"; I61.2, "Intracerebral haemorrhage in hemisphere, unspecified"; I61.3, "Intracerebral haemorrhage in brain stem"; I61.4, "Intracerebral haemorrhage in cerebellum"; I61.5, "Intracerebral haemorrhage, intraventricular"; I61.6, "Intracerebral haemorrhage, multiple localized"; I61.8, "Other intracerebral haemorrhage"; I61.9, "Intracerebral haemorrhage, unspecified"; I63.0, "Cerebral infarction due to thrombosis of precerebral arteries"; I63.1, "Cerebral infarction due to embolism of precerebral arteries"; I63.2, "Cerebral infarction due to unspecified occlusion or stenosis of precerebral arteries"; I63.3, "Cerebral infarction due to thrombosis of cerebral arteries"; I63.4, "Cerebral infarction due to embolism of cerebral arteries"; I63.5, "Cerebral infarction due to unspecified occlusion or stenosis of cerebral arteries"; I63.6, "Cerebral infarction due to cerebral venous thrombosis, nonpyogenic"; I63.8, "Other cerebral infarction"; I63.9, "Cerebral infarction, unspecified"; and I64.X, "Stroke, not specified as haemorrhage or infarction".

<u>Respiratory infection</u>: J12.X, "Viral pneumonia, not elsewhere classified: bronchopneumonia due to viruses other than influenza viruses"; J13, "Pneumonia due to *Streptococcus pneumoniae*"; J14, "Pneumonia due to *Haemophilus influenzae*"; J15.X, "Bacterial pneumonia, not elsewhere classified: bronchopneumonia due to bacteria other than *S. pneumoniae* and *H. influenzae*"; J18.0, "Bronchopneumonia, unspecified. Excluding bronchiolitis"; J18.1, "Lobar pneumonia, unspecified"; J18.2, "Hypostatic pneumonia, unspecified"; J18.8, "Other pneumonia, organism unspecified"; J18.9, "Pneumonia, unspecified"; J22, "Unspecified acute lower respiratory infection"; J44.0, "Chronic

For peer review only



For peer review only - http://bmjopen.bmj.com/site/about/guidelines.xhtml

obstructive pulmonary disease with acute lower respiratory infection. Excluding with influenza"; J44.1, "Chronic obstructive pulmonary disease with acute exacerbation, unspecified"; J69.0, "Pneumonitis due to food and vomit. Excluding Mendelson syndrome"; J69.1, "Pneumonitis due to oils and essences"; J69.8, "Pneumonitis due to other solids and liquids. Pneumonitis due to aspiration of blood"; and J85.1, "Abscess of lung with pneumonia. Excluding with pneumonia due to specified organism".

Acute myocardial infarction: I21.0, "Acute transmural myocardial infarction of anterior wall"; I21.1, "Acute transmural myocardial infarction of inferior wall"; I21.2, "Acute transmural myocardial infarction of other sites"; I21.3, "Acute transmural myocardial infarction of unspecified site"; I21.4, "Acute subendocardial myocardial infarction"; and I21.9, "Acute myocardial infarction, unspecified".

Pulmonary embolism/deep vein thrombosis: I80.1, "Phlebitis and thrombophlebitis of superficial vessels of lower extremities"; I80.1, "Phlebitis and thrombophlebitis of femoral vein"; I80.3, "Phlebitis and thrombophlebitis of other deep vessels of lower extremities"; I26.0, "Pulmonary embolism with mention of acute cor pulmonale"; and I26.9, "Pulmonary embolism without mention of acute cor pulmonale".

Urinary tract infection: N30.0, "Acute cystitis. Excluding irradiation cystitis and trigonitis"; and N39.0, "Urinary tract infection, site not specified".

Wound disruption: T81.3, "Disruption of operation wound, not elsewhere classified".

Surgical site infection: T81.4, "Infection following a procedure, not elsewhere classified".

Fracture after implant: M96.6, "Fracture of bone following insertion of orthopaedic implant, joint prosthesis, or bone plate. Excluding complication of internal orthopaedic devices, implants or grafts".



For peer review only - http://bmjopen.bmj.com/site/about/guidelines.xhtml

Complication of prosthesis: T84.0, “Mechanical complication of internal joint prosthesis”.

Neurovascular injury: T81.2, “Accidental puncture and laceration during a procedure, not elsewhere classified. Accidental perforation of: blood vessel, nerve or organ by: catheter, endoscope, instrument or probe during a procedure”.

Acute renal failure: N17.0, “Acute renal failure with tubular necrosis”; N17.1, “Acute renal failure with acute cortical necrosis”; N17.2, “Acute renal failure with medullary necrosis”; N17.8, “Other acute renal failure”; and N17.9, “Acute renal failure, unspecified”.

For peer review only



For peer review only - http://bmjopen.bmj.com/site/about/guidelines.xhtml

**Appendix 4.** Operative procedure codes (OPCS 4.8) that we used to identify blood-transfusion complication in the Hospital Episode Statistics (HES) registry.

X33.2, "Intravenous blood transfusion of packed cells"; X33.3, "Intravenous blood transfusion of platelets"; X33.8, "Other specified other blood transfusion"; X33.9, "Unspecified other blood transfusion"; X33.1, "Intra-arterial blood transfusion"; X33.7, "Autologous transfusion of red blood cells"; X34.1, "Transfusion of coagulation factor"; X34.2, "Transfusion of plasma not elsewhere classified"; X34.3, "Transfusion of serum not elsewhere classified"; and X34.4, "Transfusion of blood expander".

For peer review only



For peer review only - http://bmjopen.bmj.com/site/about/guidelines.xhtml

**Appendix 5.** Codes defined in the International Statistical Classification of Diseases and Related Health Problems 10th Revision (ICD-10) that we used to identify comorbidities in the Hospital Episode Statistics (HES) registry.

Myocardial infarction: I21, “Acute myocardial infarction”; and I22, “Subsequent myocardial infarction”.

Congestive heart failure: I50.0, “Congestive heart failure”.

Peripheral vascular disease: I70, “Atherosclerosis”; I71, “Aortic aneurysm and dissection”; I72, “Other aneurysm and dissection”; and I73, “Other peripheral vascular diseases”.

Cerebrovascular disease: I60, “Subarachnoid haemorrhage”; I61, “Intracerebral haemorrhage”; I62, “Other nontraumatic intracranial haemorrhage”; I63, “Cerebral infarction”; I64, “Stroke, not specified as haemorrhage or infarction”; I65, “Occlusion and stenosis of precerebral arteries, not resulting in cerebral infarction”; I66, “Occlusion and stenosis of cerebral arteries, not resulting in cerebral infarction”; and I67, “Other cerebrovascular diseases”.

Dementia: F00, “Dementia in Alzheimer disease”; F01, “Vascular dementia”; F02, “Dementia in other diseases classified elsewhere”; and F03, “Unspecified dementia”.

Chronic obstructive pulmonary disease: J41, “Simple and mucopurulent chronic bronchitis”; J42, “Unspecified chronic bronchitis”; J43, “Emphysema”; J44, “Other chronic obstructive pulmonary disease”; J45, “Asthma”; J46, “Status asthmaticus”; and J47, “Bronchiectasis”.

Connective tissue disease: M05, “Seropositive rheumatoid arthritis”; M06, “Other rheumatoid arthritis”; M08, “Juvenile arthritis”; M15, “Polyarthrosis”; M16, “Coxarthrosis [arthrosis of hip]”; M17, “Gonarthrosis [arthrosis of knee]”; M18, “Arthrosis of first carpometacarpal joint”; M19, “Other arthrosis” ; M35, “Other systemic involvement of



For peer review only - http://bmjopen.bmj.com/site/about/guidelines.xhtml

connective tissue" ; and M36, "Systemic disorders of connective tissue in diseases classified elsewhere".

Peptic ulcer disease: K25, "Gastric ulcer"; K26, "Duodenal ulcer"; K27, "Peptic ulcer, site unspecified"; and K28, "Gastrojejunal ulcer".

Mild liver disease: K70.0, "Alcoholic fatty liver"; K76.0, "Fatty (change of) liver, not elsewhere classified"; and K76.1 "Chronic passive congestion of liver".

Mild diabetes (without end organ damage - include ketoacidosis and coma): E10.X, "Insulin-dependent diabetes mellitus"; E10.0, "Insulin-dependent diabetes mellitus with coma"; E10.1, "Insulin-dependent diabetes mellitus with ketoacidosis" ; E10.9, "Insulin-dependent diabetes mellitus without complications"; E11.X, "Non-insulin-dependent diabetes mellitus"; E11.0, "Non-insulin-dependent diabetes mellitus with coma"; E11.1, "Non-insulin-dependent diabetes mellitus with ketoacidosis" ; E11.9, "Non-insulin-dependent diabetes mellitus without complications"; E12.X, "Malnutrition-related diabetes mellitus"; E12.0, "Malnutrition-related diabetes mellitus with coma"; E12.1, "Malnutrition-related diabetes mellitus with ketoacidosis"; E12.9, "Malnutrition-related diabetes mellitus without complications"; E13.X, "Other specified diabetes mellitus"; E13.0, "Other specified diabetes mellitus with coma"; E13.1, "Other specified diabetes mellitus with ketoacidosis"; E13.9, "Other specified diabetes mellitus without complications"; E14.X, "Unspecified diabetes mellitus"; E14.0, "Unspecified diabetes mellitus with coma"; E14.1, "Unspecified diabetes mellitus with ketoacidosis"; and E14.9, "Unspecified diabetes mellitus without complications" .

Hemiplegia: G81, "Hemiplegia. Excluding congenital cerebral palsy"



For peer review only - http://bmjopen.bmj.com/site/about/guidelines.xhtml

Moderate/severe renal disease: N17, “Acute renal failure”; N18, “Chronic kidney disease”; and N19, “Unspecified kidney failure”.

Severe diabetes (i.e. with organ damage): E10, “Insulin-dependent diabetes mellitus”; E11, “Non-insulin-dependent diabetes mellitus”; E12, “Malnutrition-related diabetes mellitus”; E13, “Other specified diabetes mellitus”; or E14, “Unspecified diabetes mellitus”. Complicated with: .2, “With renal complications”; .3 “With ophthalmic complications”; .4, “With neurological complications”; .5, “With peripheral circulatory complications”; .6, “With other specified complications”; .7, “With multiple complications”; or .8, “With unspecified complications”. N083, “Glomerular disorders in diabetes mellitus”.

Tumour: C00-C75, “Malignant neoplasms, stated or presumed to be primary, of specified sites, except of lymphoid, haematopoietic and related tissue”; C76, “Malignant neoplasm of other and ill-defined sites”; C80, “Malignant neoplasm, without specification of site”; C88, “Malignant immunoproliferative diseases”; C90.0, “Multiple myeloma”; C90.2, “Extramedullary plasmacytoma”; C96, “Other and unspecified malignant neoplasms of lymphoid, haematopoietic and related tissue”; C97, “Malignant neoplasms of independent (primary) multiple sites”; D00-D09, “In situ neoplasms”; D10-D36, “Benign neoplasms”; and D37-D48, “Neoplasms of uncertain or unknown behaviour”.

Leukaemia: C90.1, “Plasma cell leukaemia”; C91, “Lymphoid leukaemia”; C92, “Myeloid leukaemia”; C93, “Monocytic leukaemia”; C94, “Other leukaemias of specified cell type”; and C95 “Leukaemia of unspecified cell type”.

Lymphoma: C81, “Hodgkin lymphoma”; C82, “Follicular lymphoma”; C83, “Non-follicular lymphoma”; C84, “Mature T/NK-cell lymphomas”; and C85, “Other and unspecified types of non-Hodgkin lymphoma”.



For peer review only - http://bmjopen.bmj.com/site/about/guidelines.xhtml

Moderate/severe liver disease: K70, "Alcoholic liver disease except"; K71, "Toxic liver disease"; K72, "Hepatic failure, not elsewhere classified"; K73, "Chronic hepatitis, not elsewhere classified"; K74, "Fibrosis and cirrhosis of liver"; K75, "Other inflammatory liver diseases"; and K76, "Other diseases of liver". Excluding codes for mild liver disease: K70.0, K76.0 and K76.1.

AIDS: B20, "HIV disease resulting in infectious and parasitic diseases"; B21, "HIV disease resulting in malignant neoplasms"; B22, "HIV disease resulting in other specified diseases"; and B23 "HIV disease resulting in other conditions".

Metastatic solid tumour: C77, "Secondary and unspecified malignant neoplasm of lymph nodes"; C78, "Secondary malignant neoplasm of respiratory and digestive organs"; and C79, "Secondary malignant neoplasm of other and unspecified sites".

For peer review only



For peer review only - http://bmjopen.bmj.com/site/about/guidelines.xhtml

STROBE Statement—Checklist of items that should be included in reports of ***cohort studies***

| | Item No | Checked and present | Recommendation |
|---|---|---|---|
| **Title and abstract** | 1 | Page 3 abstract | (*a*) Indicate the study's design with a commonly used term in the title or the abstract |
| | | Page 3 | (*b*) Provide in the abstract an informative and balanced summary of what was done and what was found |
| **Introduction** | | | |
| Background/rationale | 2 | Pages 5-6 | Explain the scientific background and rationale for the investigation being reported |
| Objectives | 3 | Page 6 | State specific objectives, including any prespecified hypotheses |
| **Methods** | | | |
| Study design | 4 | Pages 9-10 | Present key elements of study design early in the paper |
| Setting | 5 | Page 6 | Describe the setting, locations, and relevant dates, including periods of recruitment, exposure, follow-up, and data collection |
| Participants | 6 | Page 9 | (*a*) Give the eligibility criteria, and the sources and methods of selection of participants. Describe methods of follow-up |
| | | NA | (*b*) For matched studies, give matching criteria and number of exposed and unexposed |
| Variables | 7 | Pages 7-8 | Clearly define all outcomes, exposures, predictors, potential confounders, and effect modifiers. Give diagnostic criteria, if applicable |
| Data sources/ measurement | 8* | Pages 6-9 | For each variable of interest, give sources of data and details of methods of assessment (measurement). Describe comparability of assessment methods if there is more than one group |
| Bias | 9 | Pages 9 | Describe any efforts to address potential sources of bias |
| Study size | 10 | Page 9 | Explain how the study size was arrived at |
| Quantitative variables | 11 | Page 7-8 | Explain how quantitative variables were handled in the analyses. If applicable, describe which groupings were chosen and why |
| Statistical methods | 12 | Page 10-11 | (*a*) Describe all statistical methods, including those used to control for confounding |
| | | Page 10-11 | (*b*) Describe any methods used to examine subgroups and interactions |
| | | Page 9 | © Explain how missing data were addressed |
| | | NA | (*d*) If applicable, explain how loss to follow-up was addressed |
| | | NA | (*e*) Describe any sensitivity analyses |
| **Results** | | | |
| Participants | 13* | Page 12, figure 1 | (a) Report numbers of individuals at each stage of study—eg numbers potentially eligible, examined for eligibility, confirmed eligible, included in the study, completing follow-up, and analysed |
| | | Figure 1 | (b) Give reasons for non-participation at each stage |


For peer review only - http://bmjopen.bmj.com/site/about/guidelines.xhtml

| | | | |
|---|---|---|---|
| | | Figure 1 | © Consider use of a flow diagram |
| Descriptive data | 14* | Page 12 | (a) Give characteristics of study participants (eg demographic, clinical, social) and information on exposures and potential confounders |
| | | Page 13, Figure 1, Supplementary Tables S4-S5 | (b) Indicate number of participants with missing data for each variable of interest |
| | | Page 15 | © Summarise follow-up time (eg, average and total amount) |
| Outcome data | 15* | Pages 13-15 | Report numbers of outcome events or summary measures over time |
| Main results | 16 | Pages 13-15, Table 1 Supplementary Tables S1-S3 and S6-S7 | (*a*) Give unadjusted estimates and, if applicable, confounder-adjusted estimates and their precision (eg, 95% confidence interval). Make clear which confounders were adjusted for and why they were included |
| | | Pages 13-15, Table 1 Supplementary Tables S1-S3 and S6-S7 | (*b*) Report category boundaries when continuous variables were categorized |
| | | NA | © If relevant, consider translating estimates of relative risk into absolute risk for a meaningful time period |
| Other analyses | 17 | Figures 3 and 4 | Report other analyses done—eg analyses of subgroups and interactions, and sensitivity analyses |
| **Discussion** | | | |
| Key results | 18 | Page 15 | Summarise key results with reference to study objectives |
| Limitations | 19 | Pages 15-16 | Discuss limitations of the study, taking into account sources of potential bias or imprecision. Discuss both direction and magnitude of any potential bias |
| Interpretation | 20 | Pages 17-19 | Give a cautious overall interpretation of results considering objectives, limitations, multiplicity of analyses, results from similar studies, and other relevant evidence |
| Generalisability | 21 | Pages 17-19 | Discuss the generalisability (external validity) of the study results |
| **Other information** | | | |
| Funding | 22 | Page 20 | Give the source of funding and the role of the funders for the present study and, if applicable, for the original study on which the present article is based |

*Give information separately for exposed and unexposed groups.

**Note:** An Explanation and Elaboration article discusses each checklist item and gives methodological background and published examples of transparent reporting. The STROBE checklist is best used in conjunction with this article (freely available on the Web sites of PLoS Medicine at http://www.plosmedicine.org/, Annals of Internal Medicine at http://www.annals.org/, and Epidemiology at http://www.epidem.com/). Information on the STROBE Initiative is available at http://www.strobe-statement.org.

For peer review only
